# Discrete Wavelet Transform Analysis of the Electroretinogram in Autism Spectrum Disorder and Attention Deficit Hyperactivity Disorder

**DOI:** 10.3389/fnins.2022.890461

**Published:** 2022-06-06

**Authors:** Paul A. Constable, Fernando Marmolejo-Ramos, Mercedes Gauthier, Irene O. Lee, David H. Skuse, Dorothy A. Thompson

**Affiliations:** ^1^College of Nursing and Health Sciences, Caring Futures Institute, Flinders University, Adelaide, SA, Australia; ^2^Centre for Change and Complexity in Learning, The University of South Australia, Adelaide, SA, Australia; ^3^Department of Ophthalmology & Visual Sciences, Faculty of Medicine and Health Sciences, McGill University, Montréal, QC, Canada; ^4^Behavioural and Brain Sciences Unit, Population, Policy and Practice Programme, UCL Great Ormond Street Institute of Child Health, University College London, London, United Kingdom; ^5^The Tony Kriss Visual Electrophysiology Unit, Clinical and Academic Department of Ophthalmology, Great Ormond Street Hospital for Children NHS Trust, London, United Kingdom; ^6^UCL Great Ormond Street Institute of Child Health, University College London, London, United Kingdom

**Keywords:** discrete wavelet transform, electroretinogram, retina, neurodevelopment, autism, attention deficit hyperactivity disorder

## Abstract

**Background:**

To evaluate the electroretinogram waveform in autism spectrum disorder (ASD) and attention deficit hyperactivity disorder (ADHD) using a discrete wavelet transform (DWT) approach.

**Methods:**

A total of 55 ASD, 15 ADHD and 156 control individuals took part in this study. Full field light-adapted electroretinograms (ERGs) were recorded using a Troland protocol, accounting for pupil size, with five flash strengths ranging from –0.12 to 1.20 log photopic cd.s.m^–2^. A DWT analysis was performed using the Haar wavelet on the waveforms to examine the energy within the time windows of the a- and b-waves and the oscillatory potentials (OPs) which yielded six DWT coefficients related to these parameters. The central frequency bands were from 20–160 Hz relating to the a-wave, b-wave and OPs represented by the coefficients: a20, a40, b20, b40, op80, and op160, respectively. In addition, the b-wave amplitude and percentage energy contribution of the OPs (%OPs) in the total ERG broadband energy was evaluated.

**Results:**

There were significant group differences (*p* < 0.001) in the coefficients corresponding to energies in the b-wave (b20, b40) and OPs (op80 and op160) as well as the b-wave amplitude. Notable differences between the ADHD and control groups were found in the b20 and b40 coefficients. In contrast, the greatest differences between the ASD and control group were found in the op80 and op160 coefficients. The b-wave amplitude showed both ASD and ADHD significant group differences from the control participants, for flash strengths greater than 0.4 log photopic cd.s.m^–2^ (*p* < 0.001).

**Conclusion:**

This methodological approach may provide insights about neuronal activity in studies investigating group differences where retinal signaling may be altered through neurodevelopment or neurodegenerative conditions. However, further work will be required to determine if retinal signal analysis can offer a classification model for neurodevelopmental conditions in which there is a co-occurrence such as ASD and ADHD.

## Introduction

Altered retinal signaling in neurodevelopmental conditions such as autism spectrum disorder (ASD) (Ritvo et al., 1988; Constable et al., 2016, 2020b), schizophrenia and bipolar disorder (Hébert et al., 2010, 2015, 2020; Lavoie et al., 2014; Maziade et al., 2022) have been reported with differences between these groups and controls in time-domain parameters of the electroretinogram (ERG) waveform. Whilst this method is widely employed in cardiology (Addison, 2005; Meziani et al., 2013) and neurology (Bullmore et al., 2003; Faust et al., 2015), wavelet analysis has not been used previously to study time-frequency domain of the ERG in the neurodevelopmental disorders ASD and Attention Deficit Hyperactivity Disorder (ADHD). Discrete Wavelet Transform (DWT) analysis may provide biomarkers that facilitate stratification of these neurodevelopmental conditions (Molloy and Gallagher, 2021) which are known to share substantial genetic risk (Rommelse et al., 2010; Brainstorm Consortium et al., 2018, Cao et al., 2022).

Autism spectrum disorder and ADHD are the main neurodevelopmental disorders diagnosed in early childhood with a global prevalence estimated at approximately 1 and 3.4%, respectively (Polanczyk et al., 2015; Nevison et al., 2018). The co-occurrence of neurodevelopmental conditions is high with approximately 1:3 children with ASD also meeting diagnostic criteria for ADHD (Berenguer-Forner et al., 2015). ASD and ADHD often present as co-occurrence in children (Russell et al., 2014; Mansour et al., 2017) as well as other co-occurrences of conditions such as anxiety, epilepsy, and sleep disorders (Bougeard et al., 2021). Visual perception in ASD shows superiority in visual search (Constable et al., 2020a) and abnormal electrophysiological cortical differences in global motion perception (van der Hallen et al., 2019), motion onset (Constable et al., 2012), and coherence thresholds perception (Robertson et al., 2014). For reviews of sensory and visual perception in ASD see Dakin and Frith (2005) and Robertson and Baron-Cohen (2017). Individuals with ADHD also display differences in visual tasks and perception relating to visual attention (Zarka et al., 2021) and visual search (Mullane and Klein, 2008). Sensory processing problems are common in both ASD and ADHD children and a measure of sensory function using the ERG may help our understanding of the differences and similarities of these two groups (Dellapiazza et al., 2021).

The retina has three cell types connected in a vertical signaling pathway from the photoreceptors to the bipolar cells and then to the ganglion cells. Lateral neurons modify this path at two points: horizontal cells feedback to regulate the signal between photoreceptors and bipolar cells and amacrine cells link bipolar and ganglion cells (Masland, 2012). The retinal signal in response to brief flashes of light is captured as the ERG waveform. This is composed of an initial a-wave, a negative deflection originating from the hyperpolarization of the photoreceptors. The b-wave is a positive peak following the a-wave and is formed principally by the depolarization of bipolar cells. The cone and rod bipolar cells contribute to either the ON- or OFF- pathways within the retina (Kaneda, 2013). The ON-bipolar cells use slower metabotropic glutamate receptors and respond to an increase in retinal illumination, whilst- the OFF-bipolar cells utilize faster ionotropic glutamate receptors and respond when there is a decrease in retinal illumination (Severns and Johnson, 1993; Hanna and Calkins, 2006, 2007). The a-wave of the light-adapted ERG is shaped principally by the hyperpolarization of the cones but also has post receptoral contributions from bipolar cells (Bush and Sieving, 1994; Robson et al., 2003; Friedburg et al., 2004). Inhibitory pathways are formed by the horizontal and amacrine cells that utilize GABA and dopamine as the principal inhibitory neurotransmitters respectively (Diamond, 2017). The Oscillatory Potentials (OPs) are high frequency waves that appear on the b-wave and contribute to its amplitude and are initiated by the amacrine cells with some ganglion and bipolar cell modulation (Wachtmeister, 1980, 1981, 1998, 2001).

The DWT allows the energy to be determined within frequency bands at discrete time windows within the whole ERG waveform. The method was first applied to the ERG by Gauvin et al. (2014) who used a DWT derived from a continuous wavelet transform function to extract the energy associated within the frequency bands centered on: 20, 40, 80 and 160 Hz within the a- and b-wave time windows. The group then quantified the relative contributions of the DWT coefficients within each frequency band to specific sub-components of the ON- (20 Hz) and OFF- (40 Hz) pathway responses within the a- and b-waves (termed a20, a40, b20, b40) as well as characterizing the slow and fast OPs at 80 (op80) and 160 Hz (op160), respectively (Gauvin et al., 2014, 2015, 2016, 2017). This work demonstrated the fundamental strength of the DWT analysis in its power to quantify the discrete energies related to the ON- and OFF-pathways and the OPs. Our study aimed to explore the ability of the DWT analysis to identify features in the ERG waveform that could distinguish the neurodevelopmental conditions ASD and ADHD from a control group.

## Materials and Methods

### Participants

A total of 55 ASD, 15 ADHD, and 156 control individuals took part with mean age (years) ± SD of: ASD: 14.2 ± 4.9; range (6.0–27.3), ADHD: 15.8 ± 3.2; range (8.4–21.8) and control: 13.2 ± 5.0; range (3.1–26.7) Kruskal–Wallis test (*p* < 0.001) although age would not be a factor in this young population with ERG amplitudes remaining stable from ages 15 to 24 years before media opacities influence the amount of light reaching the retina (Birch and Anderson, 1992). Within the control group 22 participants (14%) were siblings of an ASD participant which provided a more representative sample of the general population. The sex balance for each group was: ADHD 8 male: 7 female, control 50 male, and 112 female and in the ASD group 40 male and 15 females [χ^2^(2), 28.1 *p* < 0.001].

All participants were recruited at two sites from existing databases or local autism groups and *via* social media. Electrophysiological testing typically occurred in the afternoon for the participants. All ASD participants met criteria for a diagnostic classification based on DSM-IV-TR (American Psychiatric Association [APA], 2000) or DSM-5 (American Psychiatric Association [APA], 2013) criteria. Clinical assessments were guided by a combination of standardized observation (Autism Diagnostic Observation Schedule; Lord et al., 1989 or ADOS-2, Gotham et al., 2007) and interview (Developmental, Dimensional and Diagnostic interview, Skuse et al., 2004). The clinical diagnosis of ADHD was based on ICD-10 Research Diagnostic Criteria, incorporating measures of Hyperactivity, Impulsivity and Inattention provided by parents/carers and schoolteachers. The diagnostic assessments were performed by pediatric psychiatrists or clinical psychologists in the social communication disorder clinics at Great Ormond Street Hospital for Children in the United Kingdom or local Child and Adolescent Mental Health clinics in South Australia.

Participants were excluded if there was a history of strabismus surgery, other syndromic or metabolic disorders, or if there was a history of brain injury. We excluded participants who had co-existing ASD and ADHD, ADD, or OCD. Cognitive abilities were measured by the age-appropriate Wechsler scales: ASD group (mean ± SD): 101 ± 20 [range 60–136 for *N* = 41 (measure available for 75% of the group)]; ADHD group: 88 ± 10 [range 72–105 for *N* = 7 (measure available for 47% of the group)].

Eight of seventy-five participants had taken a psychoactive medication on the day prior to testing; Seven (12%) in the ASD group and one participant had taken methylphenidate (6%) in the ADHD group (6%). One individual with ASD was on an antiepileptic medication but had been seizure free for 5 years. The study was approved by human research ethics committees at both sites and written informed consent was obtained from either the participant or parent/guardian/carer as required before testing.

### Electrophysiology

The ERG recording protocol has been reported previously in more detail (Constable et al., 2020b,^2021^) and followed the International Society for Clinical Electrophysiology of Vision guidelines (Robson et al., 2022). All recordings were taken under normal room luminance (350–450 Lux). Five white flash strengths at: –0.119, 0.398, 0.602, 0.949, and 1.204 log photopic cd.s.m^–2^ on a 40 cd.m^–2^ white background were randomly presented to the right and then to the left eye at 2 Hz with 60 averages per flash strength. Traces were rejected from the average if they fell above or below the 25th centile. Repeats of the recordings were made in each eye if required. The waveform data, iris color along with video and images of the electrode position below the eye were exported using the RFF extractor version 2.9.4.1 (LKC Technologies Inc., Gaithersburg, MD, United States). The iris color index is automatically reported by the RETeval as the ratio of the 25th centile gray scale between the iris and pupil at the midline. Measurements of the iris color allowed for this parameter to be accounted for given that individuals with darker irises have lower ERG amplitudes (Al Abdlseaed et al., 2010). In addition, the electrode height (Hobby et al., 2018) was accounted for from the photographic image and a scaled ruler for each eye at 5 levels from –2 to +2 representing the height of the electrode from the recommended position of 2 mm below the lid margin. A value of –1 represents the electrode placed 1 mm below the recommended position). If the electrode was positioned greater than 2 mm below the reference level, then the data were not included in the sample. The mean ± SD iris colors for the groups were: ASD 1.23 ± 0.11, ADHD 1.27 ± 0.12, and control 1.25 ± 0.12 [Kruskal–Wallis test (*p* < 0.001)]. Only waveforms with an a-wave amplitude >1 μV were included (see Supplementary Material for further details on methods).

### Discrete Wavelet Transform Analysis

The DWT function (Eq. 1) where the *DWT(j,k)* represents the wavelet coefficients at discrete frequencies (*j*) and discrete time windows (*k*), for the raw signal *x(t)* of the ERG waveform of amplitude vs. time, with ψ representing the Haar square wavelet function (Mallat, 2009).


(1) DWT(j,k)=∫-∞+∞x(t)2−j2ψ(2−jt−k)dt

The coefficients within each time window represent the energy (μV.s) within the signal which were extracted for statistical analysis. A scalogram presents the energies within each frequency band centered on (20, 40, 80, and 160 Hz) within time windows between –20–17.5 and 0–17.5 ms (a-wave), 17.5–55 ms (b-wave), and 8.125–55 ms and 8.125–55 ms (OPs) (Gauvin et al., 2015).

The DWT analysis gives results for the energy in the 20 Hz (ON-pathway) and 40 Hz (OFF-pathway) for the a- and b-wave components (Gauvin et al., 2015; Gauvin et al., 2017) as well as the 80 and 160 Hz components of the OPs that quantify the energy within the slow and fast OPs. The %OPs are measured as the percentage of the OPs energy (i.e., the 80 ops + 160 ops coefficients) to the overall ERG energy (i.e., the 20b + 40b + 80ops + 160ops coefficients), as described by Gauvin et al. (2016).

The DWT analysis was performed in MATLAB (Mathworks Inc.). See (Gauvin et al., 2014, 2015, 2016, 2017) for detailed descriptions of the DWT methodology applied to the ERG waveform. Code is available on request from Dr. Mercedes Gauthier.

### Statistical Analyses

Non-parametric pairwise comparisons were performed between groups (i.e., ASD, control, and ADHD) at each of the five flash strengths for each of the six DWT-related dependent variables, the b-wave amplitude and %OPs. The method proposed by Noguchi et al. (2020) was used *via* the “mctp” function in the “nparcomp” R package with its default settings (Tukey-type contrast, global pseudo-rank estimation method, Fisher asymptotic approximation method, and with 95% CIs). In addition to the “mctp” adjusting *p*-values for multiple comparisons, a stringent *p* < 0.005 was adopted as a cut-off of statistical significance (Benjamin et al., 2018). Confidence limits around each estimator for comparisons between groups are provided in the statistical output section of the Supplementary Material.

See Supplementary Material for statistical output and further details on the methods. The data set is available at Flinders FigShare Repository: https://doi.org/10.25451/flinders.17712347.v3

## Results

### Discrete Wavelet Transforms

**FIGURE 1:**
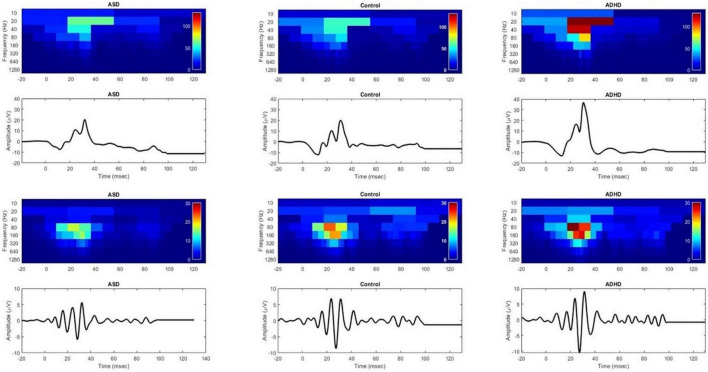
DWT scalograms of the ERG and OPs waveforms for ASD, control and ADHD normalized to the color scale of the ADHD participant using a Flash Strength of log 1.204 photopic cd.s.m^–2^. Red indicates greater energy levels and blue low energy levels. Upper panel shows the ERG waveforms and lower panels show the corresponding OP waveforms. In the upper panel the b20 and b40 bands of the ASD scalogram are not significantly different from that of the control. In contrast the ADHD participant’s recording shows increased energy within all frequency bands of the scalogram compared to the ASD and control. The lower panels, show a reduced op80 and op160 energy in the ASD participant compared to the control.

Scalogram plots of the DWT coefficients are shown in Figure 1 of the ERG and OPs waveforms normalized to the ADHD color scale for a representative individual in each group. Note the reduced energy in the OP waveform (op80 and op160) in the ASD group compared with the control but no difference in the energy levels in the ERG waveform in the b20 and b40 energies between the ASD and the control. In contrast, the energy in all the frequency bands representing the DWT coefficients is higher in the ADHD compared to the control and ASD participant in the ERG and OP waveforms. There were no significant group differences across flash strengths for the dependent variables (Mann–Whitney U test *p* > 0.09).

### Pairwise Comparisons

**FIGURE 2:**
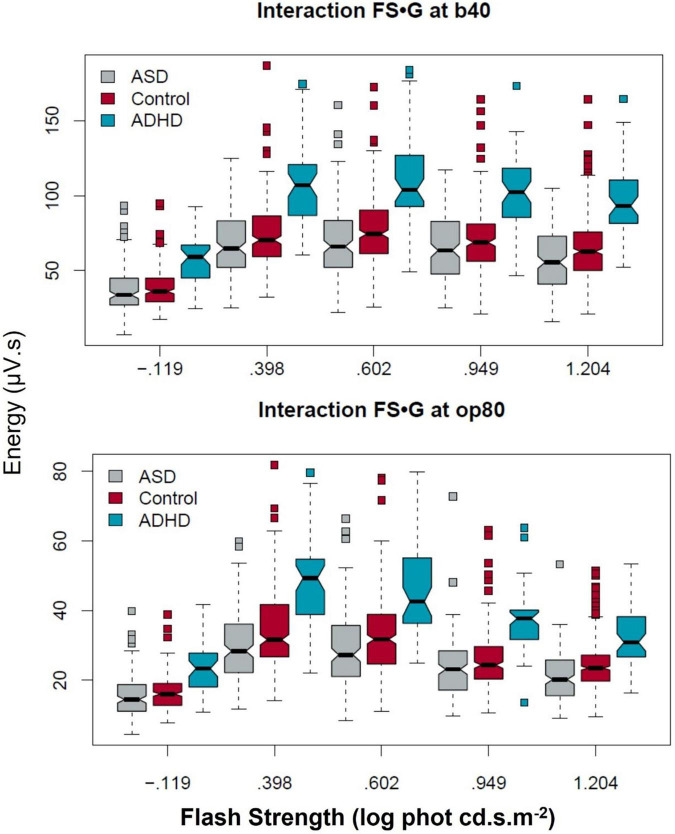
Group differences in the b40 and op80 DWT coefficients across the five flash strengths. The ADHD group exhibited higher b40 and op80 energy levels across the flash series. In contrast the difference between ASD and controls was more significantly reduced for the op80 energy compared to the b40 at the higher flash strengths. Boxplots display ∼95% CIs around the median values.

Figure 2 illustrates the group characteristics for the five flash strengths with the b40 and op80 coefficients. The boxplots display the distribution of the data with a notch at the median and the interquartile range (shown by the box itself) enables visualization of the dispersion in the data. The maverick observations are shown as the squares (see Supplementary Material Statistical Outputs for all data).

Table 1 reports the pairwise *p*-values comparison between the three groups for each of the DWT coefficients and the b-wave amplitude (b_amp) and %OPs for the two main flash strengths of 1.204 and 0.602 log photopic cd.s.m^–2^ (see Supplementary Material for all strengths). For comparison at the 1.204 log cd.s.m^–2^ the b20, b40, op80, and op160 coefficients were all significantly greater than control (*p* < 0.001) for the ADHD group. The respective energies (μV.s) for ADHD and controls at this flash strength were: Median (95% CI) ADHD group: b20 104.1 (96.0–112.6), b40 93.1 (87.0–101.6), op80 30.8 (28.7–35.9), and op160 17.9 (16.6–18.9). For the control group: b20 68.6 (65.6–71.4), b40 62.8 (61.0–65.6), op80 23.6 (23.1–24.2), and op160 13.6 (13.1–13.9). In contrast, for the ASD and control group differences at 1.204 log photopic cd.s.m^–2^ there were no significant differences for the b20 and b40 coefficients (*p* > 0.008) but there were significant differences (*p* < 0.001) for the op80 and op160 coefficients with the ASD coefficient values being: b20 65.2 (62.6–70.2), b40 55.5 (52.3–60.9), op80 20.2 (19.0–21.4), and op160 10.8 (9.9–11.7).

**TABLE 1:** Median (Mdn) and lower (L) and upper (U) 95% confidence intervals for the three groups (ASD) autism spectrum disorder, control, and attention deficit hyperactivity disorder (ADHD) at each flash strength (FS) in log photopic cd.s.m^–2^.

		ASD (a)			Control (c)			ADHD (A)			Contrast (*p*-value)		
					
FS	Parameter	Mdn	L	U	Mdn	L	U	Mdn	L	U	c v a	A v a	A v c
0.602	b wave amplitude	27.9	25.3	30.4	33.4	32.5	35.0	40.1	37.3	44.1	2.5e^–5^	< 10^–16^	1.9e^–10^
	a20 Energy	14.8	13.7	16.0	15.0	13.9	15.8	19.4	16.2	22.4	0.74	0.005	7.3e^–4^
	a40 Energy	17.2	15.7	18.6	19.6	19.0	20.5	23.4	21.0	27.8	9.7e^–5^	2.5e^–4^	0.11
	b20 Energy	63.7	60.5	68.6	65.2	63.1	68.9	95.3	89.3	100.3	0.15	< 10^–16^	<10^–16^
	b40 Energy	66.3	62.2	69.5	72.7	70.5	75.1	104.0	99.3	118.0	0.004	< 10^–16^	<10^–16^
	op80 Energy	27.3	24.9	29.8	29.4	28.0	30.4	42.6	39.7	46.5	8.3e^–4^	< 10^–16^	6.9e^–14^
	op160 Energy	13.5	12.6	14.3	16.8	16.1	17.3	19.4	18.1	21.7	1.2e^–5^	3.2e^–15^	2.4e^–7^
	%OPs	60.4	59.6	61.2	61.6	61.2	62.0	60.7	59.6	61.6	003	0.80	0.11
1.204	b wave amplitude	23.5	21.7	25.7	29.3	28.4	30.3	36.9	33.3	41.1	1.3e^–6^	1.4e^–12^	6.3e^–6^
	a20 Energy	21.4	20.2	23.8	22.5	21.2	24.0	29.5	27.2	35.3	0.65	1.3e^–6^	3.4e^–9^
	a40 Energy	21.9	19.7	23.3	26.9	25.9	27.5	27.3	22.0	31.1	6.7e^–7^	0.004	0.99
	b20 Energy	65.2	62.6	70.2	68.6	65.6	71.4	104.1	96.0	112.6	0.72	< 10^–16^	<10^–16^
	b40 Energy	55.5	52.3	60.9	62.8	61.0	65.6	93.1	87.0	101.6	0.008	< 10^–16^	<10^–16^
	op80 Energy	20.2	19.0	21.4	23.6	23.1	24.2	30.8	28.7	35.9	1.5e^–5^	< 10^–16^	4.9e^–12^
	op160 Energy	10.8	9.9	11.7	13.6	13.1	13.9	17.9	16.6	18.9	2.7e^–7^	9.0e^–13^	1.4e^–7^
	%OPs	54.3	53.7	55.2	57.1	56.4	57.5	55.3	53.8	55.5	2.4e^–9^	0.85	4.0e^–4^

*Results of non-parametric multiple comparisons between groups at each flash strength in each of the ERG-related measures at p < 0.005. c, control; a, ASD; A, ADHD. The b-wave amplitude (μV) (b_amp) and the DWT solutions for b20, b40, op80, op160 (μV.s), and %OPs (no units).*

The %OPs as a measure of the contribution of the OPs to the overall broadband ERG energy were significantly reduced for the ASD (*p* < 0.003) across several flash strengths but only at the highest flash strength of 1.204 log photopic cd.s.m^–2^ for ADHD (*p* < 0.001) with median (95% CI) values of: ASD 54.3 (53.7–55.2), control 57.1 (56.4–57.5), and ADHD 55.3 (53.8–55.5)%.

The a20 component representing the ON-response showed a significant increased energy for ADHD compared to the control group (*p* < 0.001) only for flash strengths from 0.398 to 1.204 log photopic cd.s.m^–2^ and at the maximal flash strength of 1.204 log photopic cd.s.m^–2^ the respective values for a20 for the ADHD and control groups were: [median (95% CI)]: 29.5 (27.2–35.3) μV.s and 22.5 (21.2–24.0) μV.s. The a40 component representing the OFF-pathway was also significantly lower (*p* < 0.001) for the ASD group compared to control at the 0.602 and 1.204 log photopic cd.s.m^–2^ flash strengths with significant group differences observed between the ADHD and control group only at the two weakest flash strengths of –0.119 and 0.398 log photopic cd.s.m^–2^ (*p* < 0.001).

As previously reported, the light-adapted b-wave amplitude was reduced for ASD compared to controls (Constable et al., 2016, 2020b) and increased for ADHD compared to controls (*p* < 0.001). For example, at the 1.204 cd.s.m^–2^ flash strength the b-wave amplitudes (μV) [median (95% CI)] were: ASD 23.5 (21.7–25.7), control 30.4 (28.4–31.3), and ADHD 37.2 (33.3–41.1). See Supplementary Table 2 for all values.

There were no significant correlations between groups for the dependent variables (*p* > 0.48) using robust percentage bend correlation method (Wilcox, 2012). A larger sample and consistent diagnostic metrics would be required before any conclusions regarding correlations between the DWT parameters and severity could be applied. See Supplementary Material for details of the correlation analysis.

## Discussion

Ours is the first study to apply a DWT analysis of the ERG in individuals with a neurodevelopmental disorder. Our aim was to test the hypothesis that there would be underlying differences between ADHD and ASD in the light adapted ERG waveforms that we have previously reported as abnormal in ASD (Constable et al., 2016, 2020b). We did find a pattern of difference between the ASD and control groups OPs. Whilst some differences in the shape of the second light-adapted OP peak have been described previously in a small cohort of adults with ASD (Constable et al., 2016), we can now provide the first quantification of this component of the ERG in children with ASD. We propose that the reduced b-wave amplitude we found in association with ASD could be explained by reduced energy and contribution of the neural generators of the OPs. The OPs originate in the amacrine cells, which utilize dopamine as their main neurotransmitter (Wachtmeister, 1998, 2001). Genetic studies have suggested dopamine regulation plays a role in ASD (Liu et al., 2021; Pavăl and Micluţia, 2021). The characteristics of the coefficients op80, op160 and %OPs we observed, in relation to the b-wave, could be related to the amacrine cells and dopamine signaling, transport or storage (Wachtmeister and Dowling, 1978; Wachtmeister, 2001, 1981). Supportive evidence is provided by the observation that reduced retinal dopamine levels in Parkinson’s disease are associated with lower b-wave amplitudes (Jackson et al., 2012; Nowacka et al., 2015).

Our findings in respect of ADHD concerned the b20 and b40 coefficients of the ERG, which relate to the ON- and OFF-pathways. These use glutamate as the principal neurotransmitter. There appears to be greater energy in those coefficients than in controls. The a20 coefficient was increased in ADHD compared with controls at flash strengths from 0.40 to 1.20 log cd.s.m^–2^ implying there is an ON-response difference between groups in this component. There is no direct involvement of the ON-pathway with the a-wave of the light-adapted ERG and the 20 Hz component is representative of the generalized on-response within the retina (Gauvin et al., 2015, 2017). In contrast, the a40 or OFF-pathway component to the a-wave did not differentiate these two groups at flash strengths greater than 0.60 log photopic cd.s.m^–2^ suggesting there is a relatively normal OFF-pathway response within the time window of the a-wave in children with ADHD. The post-receptor OFF-pathway normally contributes the amplitude of light-adapted a-wave (Bush and Sieving, 1994; Robson et al., 2003; Friedburg et al., 2004). The elevated energy in DWT coefficients that we observed may reflect alterations in bipolar cell functions that contribute to the b-wave. These implicate glutamate signaling and or transport/storage as the underlying cause, and glutamic gene function is associated with hyperactivity and impulsivity in ADHD (Naaijen et al., 2017). A polymorphism in metabotropic glutamate receptors predisposes to ADHD in the Chinese Han population (Zhang et al., 2021).

The %OPs were not significantly different between ADHD and control for flash strengths lower than 1.20 log photopic cd.s.m^–2^ (*p* > 0.11) with the main coefficients contributing to the elevated b-wave in ADHD being b20 and b40. In contrast, in keeping with the reduced op80 and op160 coefficients in ASD the %OPs were significantly lower for the ASD group compared to the control group for all flash strengths greater than 0.4 log photopic cd.s.m^–2^ (*p* ≥ 0.005) supporting the reduced contribution of the OPs to the overall b-wave amplitude in ASD.

Evidence from mouse models also supports the clinical findings here. A recent study using the BTBR inbred mouse strain as an ASD model showed reductions in the b-wave response under dark and light adapted conditions (Cheng et al., 2020) as well as. Studies of an ADHD mouse model in which novel DA transporter had been knocked out have indicated ERG b-wave amplitudes are increased under light-adapted conditions, which is consistent with our findings (Dai et al., 2017). Offspring of mice exposed to valproic acid as an ASD model also show reduced dark-adapted ERG responses (Guimarães-Souza et al., 2019) in support of previous findings of adults and children (Ritvo et al., 1988; Constable et al., 2016) and a deficit in ON-pathway retinal function. Currently no dark-adapted ERG studies have been performed in ADHD and this would be of interest to determine if the elevated ON-pathway response was also present under a different state of retinal adaptation.

The pathophysiology of ADHD and ASD remains a conundrum. There are no biomarkers for either condition, and we know multiple genetic and environmental factors contribute to the phenotypes (Geurts et al., 2013; Cooper et al., 2014; Kohls et al., 2014; Ronald et al., 2014; van Steijn et al., 2014; Naaijen et al., 2017). We provide some tentative evidence for neurophysiological changes that not only differentiate both conditions from typically developing children, but also evidence that they can be distinguished from each other based on ERG characteristics. Our findings suggest an elevation in the overall energy in the ERG within the b-wave and OPs in ADHD, which is consistent with reports of greater background retinal noise in this group (Bubl et al., 2015; Lee et al., 2022). In contrast, we report a reduction in the OPs contribution to the b-wave in ASD. We suggest this could account for the reduction in the b-wave amplitude previously reported in those with ASD, under dark and light adapted conditions (Ritvo et al., 1988; Constable et al., 2016, 2020b,^2021^; Lee et al., 2022).

Discrete wavelet transform analysis has been applied to other clinical conditions. For instance, the multifocal ERG and the pattern ERG reveal a positive association with primary open angle glaucoma (Brandao et al., 2017; Hassankarimi et al., 2019). In congenital stationary night blindness, DWT analysis of the ERG and multifocal ERG highlighted the known absence of the ON-pathway involvement in the waveforms (Dorfman et al., 2020). A DWT analysis of low contrast pattern reversal visual evoked potentials at different spatial frequencies found the DWT coefficients showed greater significant differences than time domain or fast Fourier transform parameters for describing the evoked potentials (Hassankarimi et al., 2020). In cardiac disease, wavelet analysis has been used extensively for analysis of the ECG and cardiac arrhythmia (Rahul and Sharma, 2021; Li et al., 2022). It has also been used to analyze EEG signals in epilepsy (Zarei and Asl, 2021) and Parkinson’s disease (Liu et al., 2017). The application of retinal signal analysis may provide a new marker for the classification of neurodevelopmental and neurodegenerative disorders given the broad heterogeneity and the lack of sufficient objective clinical and neuroscientific evidence within current classifications (Molloy and Gallagher, 2021). There is a need for a different approach that can support more reliable diagnoses, prognoses, and better targeted treatments. The DWT methodology has the potential to provide a “biotype” based on retinal signal analysis (Clementz et al., 2016; Cuthbert, 2020) and contribute to this field. Signal analysis of the ERG waveform may also provide more subtle functional insights into the structural changes within the retina which have been documented in longitudinal studies of Alzheimer’s or Parkinson’s disease using retinal imaging techniques (Kashani et al., 2021). It may also provide a sensitive method to monitor the effects of clinical trials of pharmacological and gene therapy to manage retinal and ophthalmic diseases (Yu-Wai-Man et al., 2020; Maguire et al., 2021; Gajendran et al., 2022).

Analysis of the ERG waveform using wavelet functions [as well as other signal analysis techniques such as Variable Frequency Complex Demodulation (VFCDM), (Chon et al., 2009), or Functional Data Analysis (Calle-Saldarriaga et al., 2021)] could provide better characterization of the heterogeneity inherent within the neurodevelopmental disorders that share common clinical traits (Thapar et al., 2017; Molloy and Gallagher, 2021). Of these, VFCDM can provide one of the highest time-frequency resolutions (Chon et al., 2009) and has been used to analyze other physiological signals (Wang et al., 2006; Siu et al., 2009, Posada-Quintero et al., 2016; Hossain et al., 2021). VFCDM uses a bank of low-pass filters to decompose the signal into a suite of band-limited signals. These can estimate instantaneous amplitude, frequency, and phase within each frequency band with higher resolution than DWT. VFCDM may offer an additional process to unlock the hidden signals within the ERG of neurological and retinal conditions.

This study is the first to apply a wavelet transform approach to analyzing clinical waveforms in neurodevelopmental conditions using the ERG. The method allows an objective description of the ON- and OFF- pathways as well as the contribution of the OPs to the overall ERG signal. A combination of time domain (b-wave amplitude) and time-frequency domain parameters (b20, b40, op80, op160) may assist with developing classification models of ADHD and ASD in the future, as well as providing a simple method to extract information on the underlying contributions of the ON- and OFF- pathways without the need for modeling luminance-response functions across several flash strengths (Hamilton et al., 2007; Hébert et al., 2017; Constable et al., 2020b). We speculate that the wavelet approach may in future aid in the study of retinal neurophysiology in schizophrenia and bipolar disorder (Hébert et al., 2015, 2020) as well as neurodegenerative conditions such as Parkinson’s disease (Garcia-Martin et al., 2014; Nowacka et al., 2015). Further work will be required to establish abnormalities in these retinal signals are specific to neurodevelopmental disorders and what effects may be observed in the case of co-occurrence of other conditions (Silverstein and Thompson, 2020).

## Data Availability Statement

The datasets presented in this study can be found in online repositories. The names of the repository/repositories and accession number(s) can be found below: https://doi.org/10.25451/flinders.17712347.v3.

## Ethics Statement

This study was reviewed and approved by the Flinders University Human Research Ethics Committee and the South East Scotland Research Ethics Committee in the United Kingdom. Written informed consent to participate in this study for those under 16 years of age was provided by the participants’ legal guardian/next of kin.

## Author Contributions

PC wrote the first draft and collected ERG recordings with IL and DT. FM-R conducted the statistical analysis. DS contributed to the main discussion and clinical assessments. PC and MG performed the DWT analysis. All authors contributed equally to the final manuscript.

## Conflict of Interest

The authors declare that the research was conducted in the absence of any commercial or financial relationships that could be construed as a potential conflict of interest.

## Publisher’s Note

All claims expressed in this article are solely those of the authors and do not necessarily represent those of their affiliated organizations, or those of the publisher, the editors and the reviewers. Any product that may be evaluated in this article, or claim that may be made by its manufacturer, is not guaranteed or endorsed by the publisher.
