# Supplementary material for: Discrete Wavelet Transform Analysis of the Electroretinogram in Autism Spectrum Disorder and Attention Deficit Hyperactivity Disorder
Source: Front Neurosci. 2022 Jun 6;16:890461. doi: 10.3389/fnins.2022.890461 (PMC9207322; doi:10.3389/fnins.2022.890461)
Supplement: Supplementary file 1 [file Data_Sheet_1.pdf]

Discrete Wavelet Transform analysis of the electroretinogram in autism spectrum disorder and attention deficit hyperactivity disorder

Contents

Methods 1
Iris Color 1
Electrode Height 3
Discrete Wavelet Transform Scalogram 4
Conversion Table for Flash Strength 5
References 6
Statistical Outputs 7

Methods

Iris Color

The iris color ratio or index is an automated procedure performed by the RETeval to estimate the pigmentation of the iris so that iris color can be accounted for in the statistical analyses (Al Abdlseaed et al., 2010). The calculation is based on the image acquired by the RETeval during the recording and is the ratio of the 25th centile grey scale values in 1mm segments at the 3 and 9 o'clock position relative to the 25th centile grey scale value of the pupil. The figure below illustrates the image and line segments used for the calculation. The iris color index has no units and typically varies from 1.10 for pale irises to 1.50 for dark irises. (See Figure 1).

The ratio is extracted from the video taken of the eye during a Troland protocol recording by the RETeval. The ratio is an estimate of the color of the iris, where darker irises appear brighter in the infrared and therefore have a larger iris/pupil color ratio. Because of the automatic gain control of the exposure in the RETeval, the absolute values for the iris and pupil color are not

meaningful.

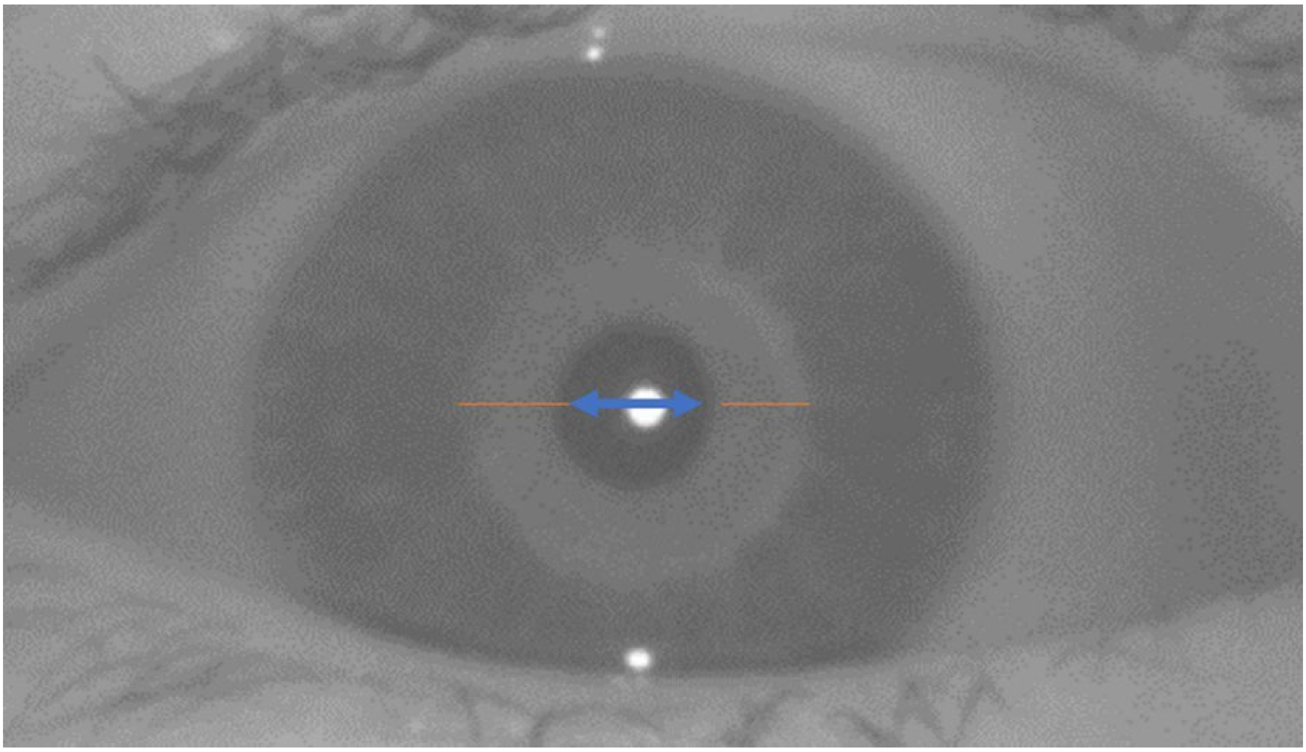

Figure 1 The iris color is the 25th percentile of the grey values found in the two 1 mm long horizontal line segments starting at the left and the right edges of the pupil cantered vertically. The pupil color is the 25th percentile of the grey values across the horizontal diameter of the pupil, excluding saturated values. Orange bars represent the 2 x 1mm line segments and blue arrow representing the diameter of the pupil. The Image is in the infrared and extracted from the video recorded during each recording.

The distribution of the iris color index is shown in figure 2 below for the participants in this study. The ASD group has slightly lighter irises- with iris color index of: 1.20 ± 0.10 compared to 1.24 ± 0.12 for the control group and 1.25 ± 0.12 for the ADHD group.

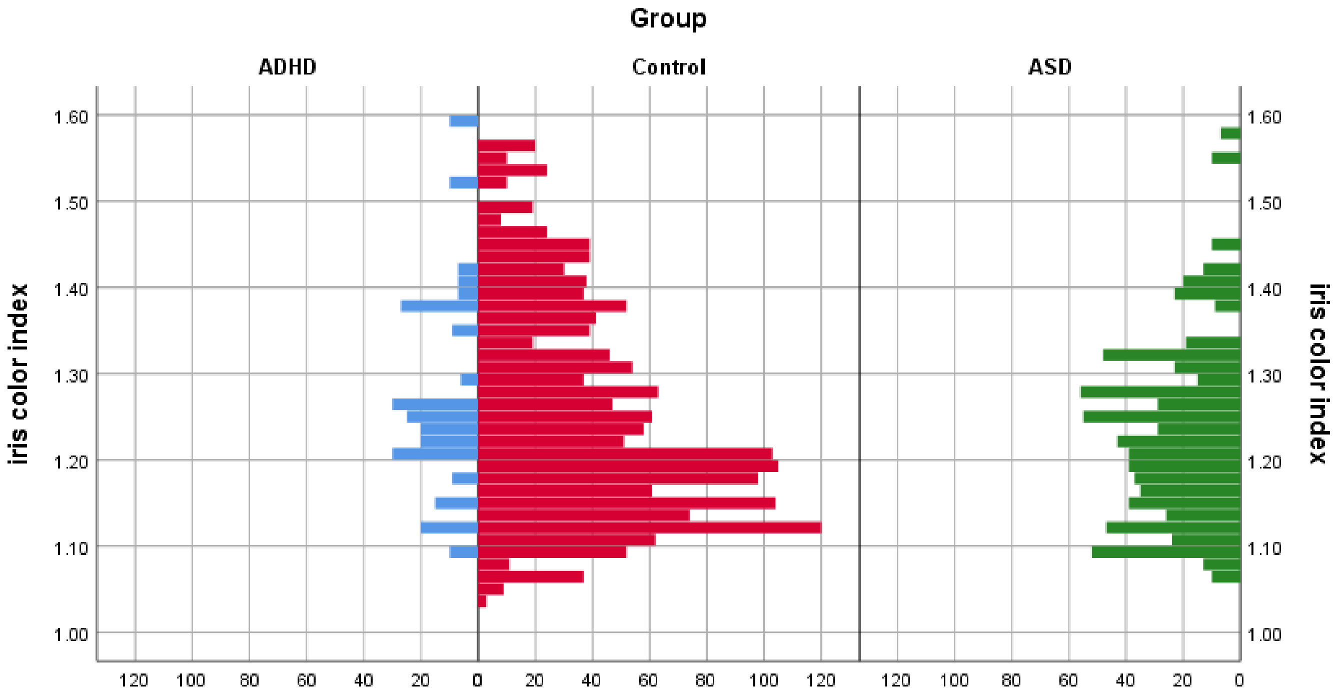

Figure 2 Distribution of iris color index for the ASD, Control Group and ADHD.

Electrode Height

The electrode position with respect to the lower lid margin was determined using the photographic images of the eye taken by the RETeval at the time of recording, A graticule, that was created by scaling the millimeter grid to the actual size of the electrode was overlaid across the photograph to determine the vertical height of the upper edge of the electrode to the lower lid margin. If the height was more than 4mm the recordings for that eye were discarded. A nominal scale of 1-4 was used to adjust the amplitudes recorded with the height of the electrode with a reference level of 2 set to zero as 2mm is the recommended height for the electrode placement. A series of images and the scaled graticule are shown below in figure 3.

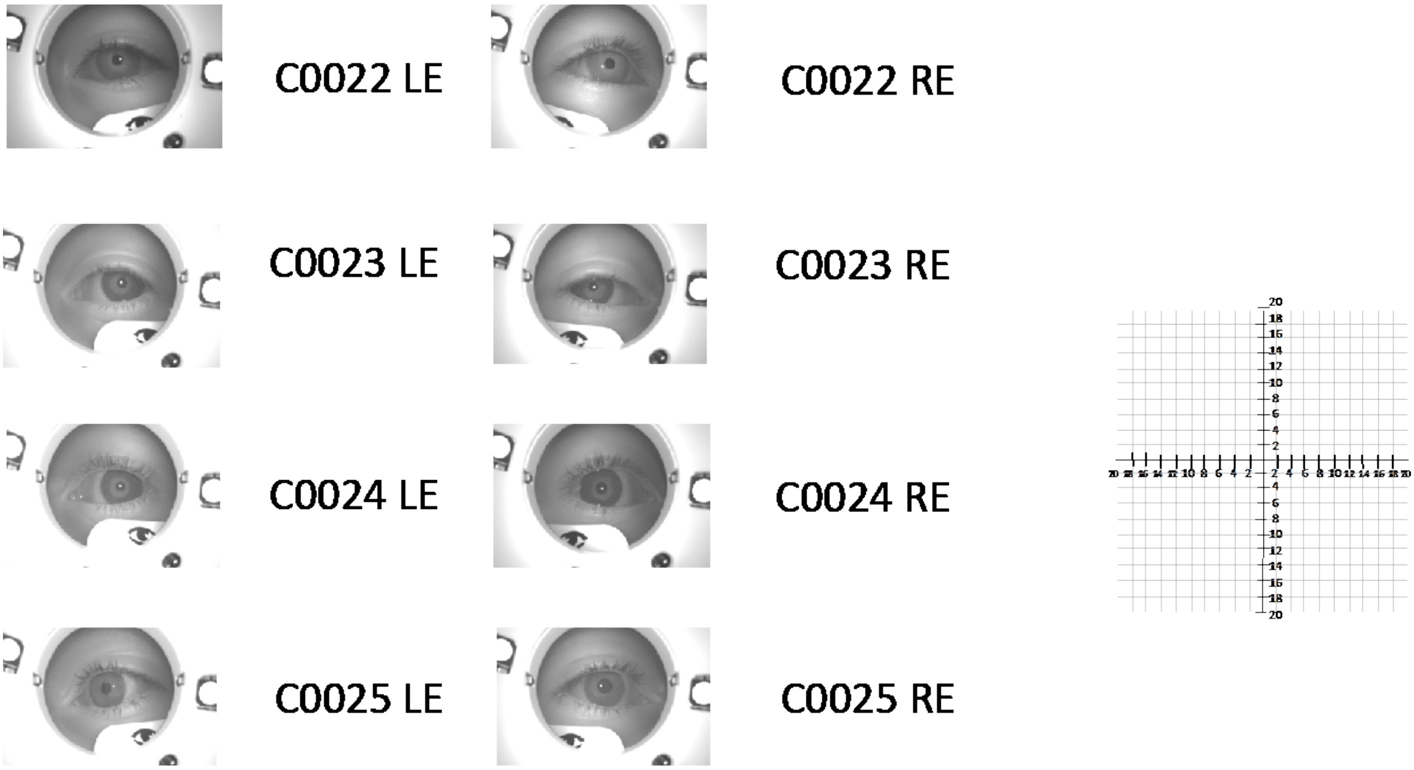

Figure 3 Electrode height was assessed using the graticule scaled to the electrode's dimensions.

Discrete Wavelet Transform Scalogram

The DWT analysis produces the coefficients corresponding to the energy (uV.s) within frequency bands of 20-160 Hz at specified time windows. The magnitude of the coefficient is then plotted as a color scale for ach frequency and within each time window. Figure 2 shows a scalogram plot of the ERG waveform, with the color scales corresponding to the energy within the time windows. From the figure there are main energies that correspond to the a- wave (a20 and a40) and the b-wave (b20 and b40). (Gauvin et al., 2017) has shown that the 20Hz relates to the ON-pathway and the 40 Hz frequency band the OFF-pathway.

When the Oscillatory potential waveform is analyzed the 80Hz related to the slow and the 160Hz relating to the fast OPs. Again, the energy is signified by the color in the scalogram plot.

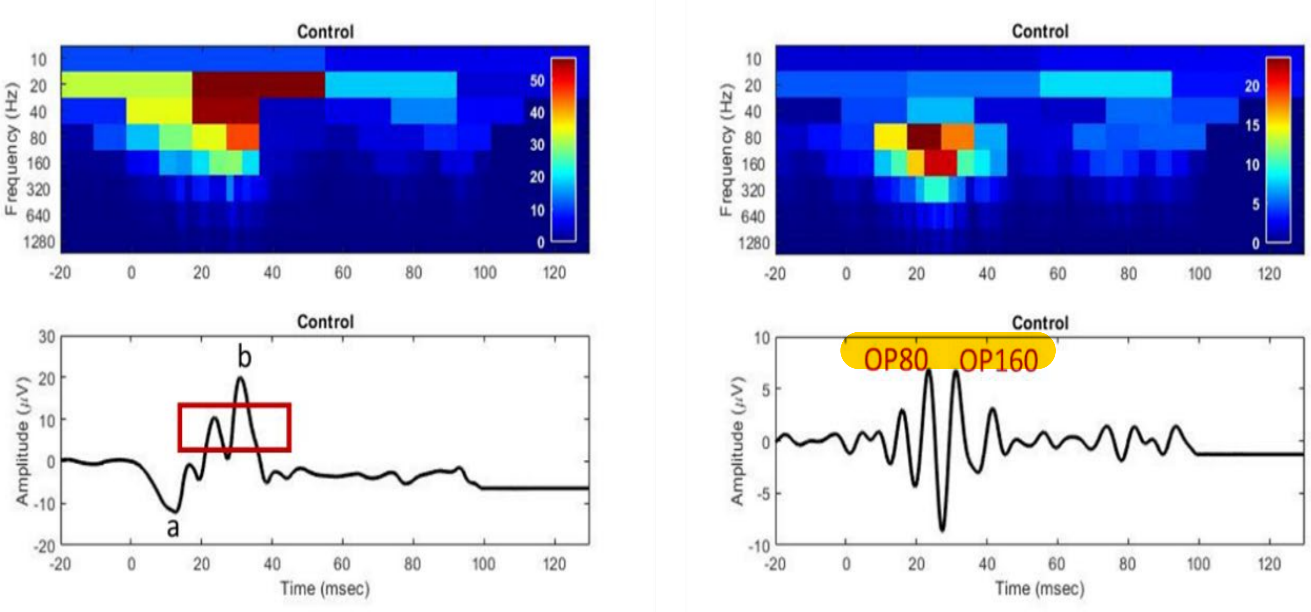

Raw electroretinogram and oscillatory potential traces from a control subject at flash strength

1.204 log photopic cd.s.m^{-2} below the colored scalogram DWT plots. In the lower panel the a- wave is a negative deflection occurring at approximately 18 msec and is related to photoreceptor hyperpolarization. The b-wave is the following positive peak and is driven mainly by bipolar cells with a peak at approximately 30 ms. The higher frequency oscillatory potentials are typically extracted using digital filtering of the raw electroretinogram trace. The red box on the ERG waveform describes the time window from which the OPs are extracted and analyzed by the DWT method. The figure on the right slows the extracted OPs from this time interval and their energies associated with the 80Hz frequency domain OP80 (slow) and the 160 Hz frequency domain OP160 (fast) highlighted. Above each raw trace is a color map 'scalogram' of the DWT coefficients that represent the energy within the distinct time windows associated with the a- and b- waves and the oscillatory potentials.

Conversion Table for Flash Strength

Table 1 shows the conversion of the Td.s flash strengths used by the RETeval into log

photopic cd.s.m^{-2} and photopic cd.s.m^{-2} assuming a 6mm diameter pupil. (Table 1).

Flash Strength		
Td.s	log photopic cd.s.m^{-2}	photopic cd.s.m^{-2}
21.48	-0.119	0.76
70.65	0.398	2.50
113.04	0.602	4.00
251.51	0.949	8.90
446.00	1.204	16.00

Table 1 Conversion table for flash strengths used in this study using the Troland based RETeval protocol assuming a 6mm diameter pupil.

References

Al Abdlseaed, A., McTaggart, Y., Ramage, T., Hamilton, R., and McCulloch, D.L. (2010). Light- and dark-adapted electroretinograms (ERGs) and ocular pigmentation: comparison of brown- and blue-eyed cohorts. Documenta ophthalmologica.Advances in ophthalmology 121(2), 135-146. doi: 10.1007/s10633-010-9240-3 [doi].

Gauvin, M., Sustar, M., Little, J.M., Brecelj, J., Lina, J.M., and Lachapelle, P. (2017). Quantifying the ON and OFF Contributions to the Flash ERG with the Discrete Wavelet Transform. Transl Vis Sci Technol 6(1), 3. doi: 10.1167/tvst.6.1.3.

Statistical Outputs

Table 2 gives the median (Mdn) and lower (L) and upper (U) 95% CI for the main outcome measures for the elctroretinogram: b-wave amplitude and DWT coefficients for a20, a40, b20, b40, op80, op160 and %OPs.

		ASD (a)			Control (c)			ADHD (A)			Contrast (*p*-value)		
FS	Parameter	Mdn	L	U	Mdn	L	U	Mdn	L	U	c v a	A v a	A v c
-.119	b wave amplitude	14.5	13.6	15.4	16.4	15.8	17.1	21.6	19.7	23.0	.050	$2.4e^{-8}$	$1.6e^{-6}$
	a20 Energy	8.1	7.0	9.1	9.7	9.0	10.3	11.7	9.2	13.4	.160	.003	.063
	a40 Energy	11.2	10.3	12.1	12.1	11.3	12.7	15.1	13.7	16.7	.284	$3.3e^{-6}$	$5.0e^{-5}$
	b20 Energy	33.7	30.9	35.5	34.9	33.2	35.6	47.7	42.2	53.1	.900	$5.4e^{-15}$	$4.4e^{-16}$
	b40 Energy	33.7	31.9	36.4	35.9	34.8	38.3	54.3	48.5	62.7	.552	$4.7e^{-14}$	$1.1e^{-13}$
	op80 Energy	14.4	13.2	15.7	16.0	15.2	16.6	21.6	19.1	26.2	.190	$1.3e^{-10}$	$1.1e^{-9}$
	op160 Energy	7.5	7.1	8.0	8.2	7.9	8.7	11.0	9.6	13.4	.126	$5.3e^{-8}$	$5.5e^{-6}$
	%OPs	59.4	58.8	60.1	60.3	59.4	60.9	60.4	59.1	61.9	.427	.311	.748
.398	b wave amplitude	28.2	27.3	30.9	32.6	31.1	34.5	45.3	40.4	47.2	$2.0e^{-4}$	$1.1e^{-15}$	$1.2e^{-9}$
	a20 Energy	12.3	10.8	13.2	13.4	12.8	14.1	16.9	14.4	20.1	.377	$8.5e^{-5}$	$7.7e^{-4}$
	a40 Energy	15.5	14.2	17.0	16.8	16.2	17.5	20.8	19.0	22.7	.098	$2.5e^{-7}$	$3.3e^{-5}$
	b20 Energy	60.2	54.6	62.7	60.5	57.2	62.9	90.6	85.0	99.8	.992	$<10^{-16}$	$<10^{-16}$
	b40 Energy	64.9	61.2	69.6	70.8	68.1	74.0	107.2	98.2	112.1	.027	$<10^{-16}$	$<10^{-16}$
	op80 Energy	28.2	26.1	30.1	31.7	30.4	34.1	49.2	44.4	52.5	$2.4e^{-4}$	$<10^{-16}$	$1.3e^{-13}$
	op160 Energy	14.4	13.6	15.4	16.3	15.7	17.4	23.6	21.0	25.0	$6.6e^{-4}$	$<10^{-16}$	$1.7e^{-11}$
	%OPs	63.4	62.5	64.2	64.4	64.1	64.9	64.5	63.6	65.3	$5.3e^{-4}$	.014	.999
.602	b wave amplitude	27.9	25.3	30.4	33.4	32.5	35.0	40.1	37.3	44.1	$2.5e^{-5}$	$<10^{-16}$	$1.9e^{-10}$
	a20 Energy	14.8	13.7	16.0	15.0	13.9	15.8	19.4	16.2	22.4	.742	.005	$7.3e^{-4}$
	a40 Energy	17.2	15.7	18.6	19.6	19.0	20.5	23.4	21.0	27.8	$9.7e^{-5}$	$2.5e^{-4}$	.111
	b20 Energy	63.7	60.5	68.6	65.2	63.1	68.9	95.3	89.3	100.3	.150	$<10^{-16}$	$<10^{-16}$
	b40 Energy	66.3	62.2	69.5	72.7	70.5	75.1	104.0	99.3	118.0	.004	$<10^{-16}$	$<10^{-16}$
	op80 Energy	27.3	24.9	29.8	29.4	28.0	30.4	42.6	39.7	46.5	$8.3e^{-4}$	$<10^{-16}$	$6.9e^{-14}$
	op160 Energy	13.5	12.6	14.3	16.8	16.1	17.3	19.4	18.1	21.7	$1.2e^{-5}$	$3.2e^{-15}$	$2.4e^{-7}$
	%OPs	60.4	59.6	61.2	61.6	61.2	62.0	60.7	59.6	61.6	003	.801	.111
.949	b wave amplitude	27.9	24.7	29.6	29.9	28.4	31.3	38.4	36.0	41.8	.048	$2.1e^{-14}$	$4.1e^{-11}$
	a20 Energy	18.2	16.6	20.2	21.6	20.7	22.8	25.9	23.3	30.6	.015	$1.6e^{-6}$	.001
	a40 Energy	19.5	17.5	21.4	22.9	21.4	23.8	25.8	21.5	30.2	.007	.001	.160
	b20 Energy	68.8	63.6	76.1	72.9	69.5	77.4	102.9	96.6	114.6	.935	$<10^{-16}$	$<10^{-16}$
	b40 Energy	63.3	59.2	67.9	68.8	66.3	71.0	101.5	95.2	108.6	.505	$<10^{-16}$	$<10^{-16}$
	op80 Energy	23.0	21.1	24.5	24.3	23.6	25.7	37.6	35.2	38.4	.037	$<10^{-16}$	$<10^{-16}$
	op160 Energy	12.3	11.6	13.7	13.3	12.6	14.0	19.5	17.8	20.5	.268	$3.2e^{-15}$	$5.9e^{-14}$
	%OPs	55.6	55.1	56.2	56.9	56.5	57.4	56.6	55.4	57.8	.005	.374	.758
1.204	b wave amplitude	23.5	21.7	25.7	29.3	28.4	30.3	36.9	33.3	41.1	$1.3e^{-6}$	$1.4e^{-12}$	$6.3e^{-6}$
	a20 Energy	21.4	20.2	23.8	22.5	21.2	24.0	29.5	27.2	35.3	.651	$1.3e^{-6}$	$3.4e^{-9}$
	a40 Energy	21.9	19.7	23.3	26.9	25.9	27.5	27.3	22.0	31.1	$6.7e^{-7}$	.004	.997
	b20 Energy	65.2	62.6	70.2	68.6	65.6	71.4	104.1	96.0	112.6	.717	$<10^{-16}$	$<10^{-16}$
	b40 Energy	55.5	52.3	60.9	62.8	61.0	65.6	93.1	87.0	101.6	.008	$<10^{-16}$	$<10^{-16}$
	op80 Energy	20.2	19.0	21.4	23.6	23.1	24.2	30.8	28.7	35.9	$1.5e^{-5}$	$<10^{-16}$	$4.9e^{-12}$
	op160 Energy	10.8	9.9	11.7	13.6	13.1	13.9	17.9	16.6	18.9	$2.7e^{-7}$	$9.0e^{-13}$	$1.4e^{-7}$
	%OPs	54.3	53.7	55.2	57.1	56.4	57.5	55.3	53.8	55.5	$2.4e^{-9}$	.849	$4.0e^{-4}$

Table 2 Median (Mdn) and lower (L) and upper (U) 95% confidence intervals for the three groups (ASD) autism spectrum disorder, control, and attention deficit hyperactivity disorder (ADHD) at each flash strength (FS) in log photopic cd.s.m^{-2}. Results of nonparametric multiple comparisons between groups at each flash strength in each of the ERG-related measures at p<.005. c = control; a = ASD; A = ADHD. The b-wave amplitude (µV) (b_amp) and the discrete wavelet transform solutions for a20, a40, b20, b40, op80, op160 (µV.s) and %OPs (no units).

Supplementary analyses DWT dataset

Fernando Marmolejo-Ramos

2021-10-13

Contents

Introduction **3**

Statistical analyses **7**

Plots 7

Statistical modelling 15

Examining the interaction $FS \cdot G$ **20**

a20 20

a40 21

op80 22

op160 23

b20 24

b40 25

b_amp 26

OP_ratio 28

Nonparametric multiple comparisons **29**

a20 29

flash -0.119 30

flash 0.398 30

flash 0.602 31

flash 0.949 32

flash 1.204 33

a40 34

flash -0.119 34

flash 0.398 35

flash 0.602 35

flash 0.949 36

flash 1.204 37
op80 38
flash -0.119 38
flash 0.398 39
flash 0.602 39
flash 0.949 40
flash 1.204 41
op160 42
flash -0.119 42
flash 0.398 43
flash 0.602 43
flash 0.949 44
flash 1.204 45
b20 46
flash -0.119 46
flash 0.398 47
flash 0.602 47
flash 0.949 48
flash 1.204 49
b40 50
flash -0.119 50
flash 0.398 51
flash 0.602 51
flash 0.949 52
flash 1.204 53
b_amp 54
flash -0.119 54
flash 0.398 55
flash 0.602 55
flash 0.949 56
flash 1.204 57
OP_ratio 58
flash -0.119 58
flash 0.398 59
flash 0.602 59
flash 0.949 60
flash 1.204 61

Correlations between groups at different flash strengths and voltages 62
Groups' sample sizes . . . 63
ADHD vs control at flash .602 and b20 . . . 64
ADHD vs control at flash 1.20 and b20 . . . 64
ADHD vs control at flash .602 and b40 . . . 65
ADHD vs control at flash 1.20 and b40 . . . 66
ASD vs control at flash .602 and b20 . . . 66
ASD vs control at flash 1.20 and b20 . . . 67
ASD vs control at flash .602 and b40 . . . 68
ASD vs control at flash 1.20 and b40 . . . 68
ADHD vs ASD at flash .602 and b20 . . . 69
ADHD vs ASD at flash 1.20 and b20 . . . 70
ADHD vs ASD at flash .602 and b40 . . . 70
ADHD vs ASD at flash 1.20 and b40 . . . 71

Introduction

The following analysis has the goal of examining the effect of several covariates on each of the ERG-related dependent variables; i.e. `b_amp`, `b20`, `b40`, `op80`, `op160`, `a20`, and `a40`.

The independent variables are (no order implied) flash strength (`strength`, FS), vertical location (`VERT`, V), iris (`iris`, I), group (`group`, G), ethnicity (`ethnicity`, E) and interaction between flash strenght and group ($FS \cdot G$). Variables not listed here, aren't considered later on.

The models to consider will thus be:

$DV \sim FS + V + I + G + E + FS \cdot G$; where DV is each of the ERG-related variables.

The (explanatory) model is analysed via robust (fixed-effects) linear regression. The ultimate goal is to see which dependent variable enables to differentiate among the three groups (i.e. ASD, control and ADHD). Thus, the pairwise differences between groups at each flash strength will be examined for each of the six ERG-related dependent variables.

First off, the data examination performed in other analyses is reproduced here.

The following sections present the analyses applied to the DWT dataset.

```
######### reading the data
library(readxl)
library(dplyr)

data <- read_excel(file.choose(), sheet = "DWT_final")

# checking missing values
library(naniar)
gg_miss_upset(data)
```

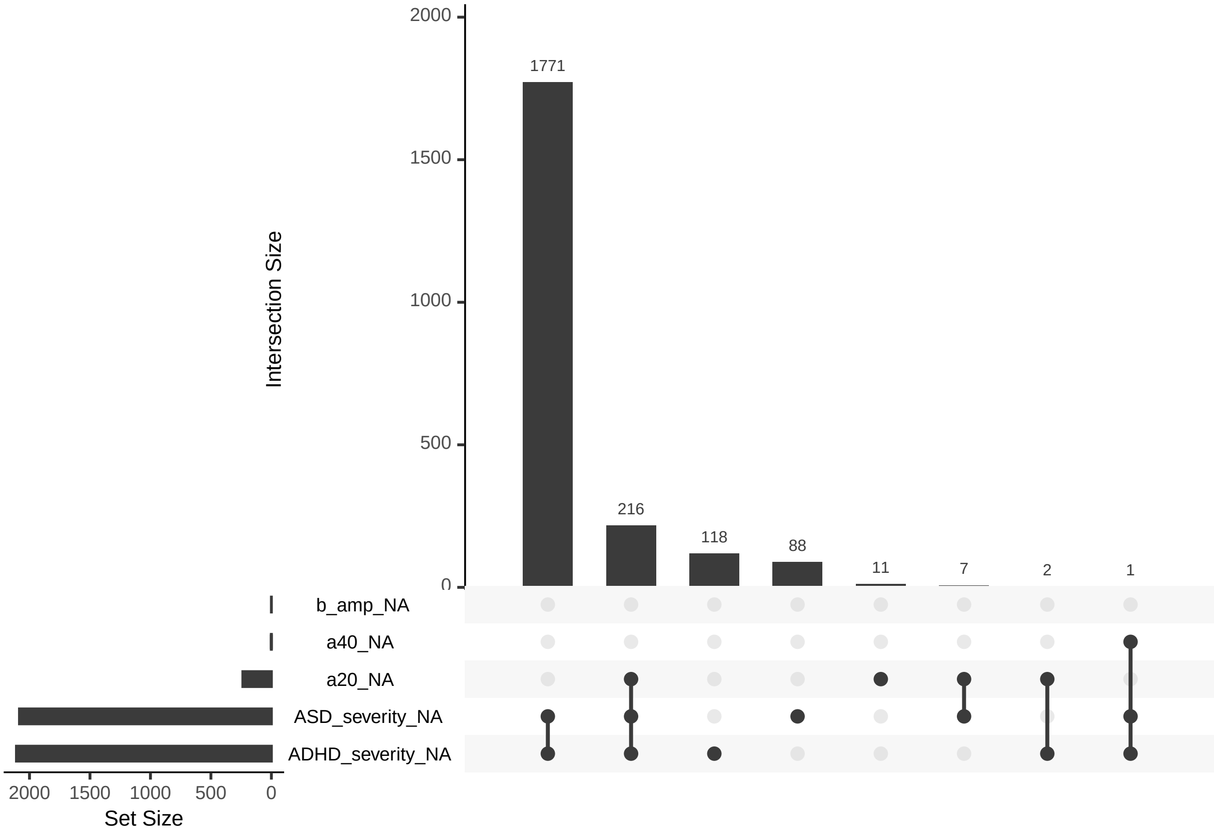

The results indicate ASD_Severity and ADHDseverity have a large number of missing values so they'll be removed from further analyses.

```
# retaining the columns of interest
# variables were suggested by the leading researcher (paul constable)
data1 <- subset(data, select=c(
  participant, gender,  group,  VERT,   Ethnicity, Sibling_of_ASD,
  CNS_MED,  eye,    iris,   strength,

  # numeric
  a20,  a40,    b20,    b40,    op80,   op160,  OPratio, b_amp))

data1 <- data1 %>%
  dplyr::rename(perc_op_ratio = OPratio)
```

Let's amend the data...

```
# first fixing the data...
str(data1)
```

```
## tibble [2,737 x 18] (S3: tbl_df/tbl/data.frame)
##  $ participant   : chr [1:2737] "a10" "a10" "a10" "a10" ...
##  $ gender        : num [1:2737] 0 0 0 0 0 0 0 0 0 0 ...
##  $ group         : num [1:2737] 0 0 0 0 0 0 0 0 0 0 ...
##  $ VERT          : num [1:2737] -2 -2 -2 -2 -2 0 0 0 0 0 ...
##  $ Ethnicity     : num [1:2737] 1 1 1 1 1 1 1 1 1 1 ...
```

```
##  $ Sibling_of_ASD: num [1:2737] 0 0 0 0 0 0 0 0 0 0 ...
##  $ CNS_MED       : num [1:2737] 0 0 0 0 0 0 0 0 0 0 ...
##  $ eye           : num [1:2737] 0 0 0 0 0 1 1 1 1 1 ...
##  $ iris          : num [1:2737] 1.12 1.12 1.12 1.12 1.12 ...
##  $ strength      : num [1:2737] -0.119 0.398 0.602 0.949 1.204 ...
##  $ a20           : num [1:2737] 3.19 5.28 7.24 20.88 26.62 ...
##  $ a40           : num [1:2737] 5 14.64 5.89 17.27 30.69 ...
##  $ b20           : num [1:2737] 38.5 38.3 24.1 45.2 56.8 ...
##  $ b40           : num [1:2737] 32.5 47.5 54.1 44.3 38.4 ...
##  $ op80          : num [1:2737] 13.1 21.6 27.7 15.7 15.7 ...
##  $ op160         : num [1:2737] 6.43 12.05 12.77 7.79 9.19 ...
##  $ perc_op_ratio : num [1:2737] 57.9 66.2 65 56.7 56.6 ...
##  $ b_amp         : num [1:2737] 8.4 22.1 27 16.5 16.2 ...
```

```
# fixing the variables of interest
# variables not listed are therefore in the right format
data.fixed <- data1 %>%
  dplyr::mutate(
    participant = as.factor(participant),
    gender = as.factor(gender),
    group = as.factor(group),
    Ethnicity = as.factor(Ethnicity),
    VERT = as.factor(VERT),
    Sibling_of_ASD = as.factor(Sibling_of_ASD),
    CNS_MED = as.factor(CNS_MED),
    eye = as.factor(eye),
    iris = as.numeric(iris),
    strength = as.factor(strength)
)

# releveling VERT
data.fixed$VERT <- relevel(data.fixed$VERT, ref = "0")
# with 0 being baseline height

# retaining complete cases
dim(data.fixed)
```

```
## [1] 2737   18
```

```
# Note: We'll stick to all cases for now
data.fixed <- data.fixed[complete.cases(data.fixed), ]
dim(data.fixed)
```

```
## [1] 2500   18
```

```
str(data.fixed)
```

```
## tibble [2,500 x 18] (S3: tbl_df/tbl/data.frame)
##  $ participant   : Factor w/ 228 levels "a10","a100","a20",..: 1 1 1 1 1 1 1 1 1 1 ...
##  $ gender        : Factor w/ 2 levels "0","1": 1 1 1 1 1 1 1 1 1 1 ...
##  $ group         : Factor w/ 3 levels "0","1","3": 1 1 1 1 1 1 1 1 1 1 ...
##  $ VERT          : Factor w/ 5 levels "0","-2","-1",..: 2 2 2 2 2 1 1 1 1 1 ...
```

```
##  $ Ethnicity     : Factor w/ 5 levels "1","2","3","4",..: 1 1 1 1 1 1 1 1 1 1 ...
##  $ Sibling_of_ASD: Factor w/ 2 levels "0","1": 1 1 1 1 1 1 1 1 1 1 ...
##  $ CNS_MED       : Factor w/ 2 levels "0","1": 1 1 1 1 1 1 1 1 1 1 ...
##  $ eye           : Factor w/ 2 levels "0","1": 1 1 1 1 1 2 2 2 2 2 ...
##  $ iris          : num [1:2500] 1.12 1.12 1.12 1.12 1.12 ...
##  $ strength      : Factor w/ 5 levels "-0.119","0.398",..: 1 2 3 4 5 1 2 3 4 5 ...
##  $ a20           : num [1:2500] 3.19 5.28 7.24 20.88 26.62 ...
##  $ a40           : num [1:2500] 5 14.64 5.89 17.27 30.69 ...
##  $ b20           : num [1:2500] 38.5 38.3 24.1 45.2 56.8 ...
##  $ b40           : num [1:2500] 32.5 47.5 54.1 44.3 38.4 ...
##  $ op80          : num [1:2500] 13.1 21.6 27.7 15.7 15.7 ...
##  $ op160         : num [1:2500] 6.43 12.05 12.77 7.79 9.19 ...
##  $ perc_op_ratio : num [1:2500] 57.9 66.2 65 56.7 56.6 ...
##  $ b_amp         : num [1:2500] 8.4 22.1 27 16.5 16.2 ...
```

Let's clean up the column names with `janitor`:

```
data.fixed <- janitor::clean_names(data.fixed)
```

```
# relabeling some of the variables' levels
library(plyr)
data.fixed$gender <-
mapvalues(data.fixed$gender,
from = c('0','1'),
to = c('Male','Female'))

data.fixed$group <-
mapvalues(data.fixed$group,
from = c('0','1', '3'),
to = c('ASD','Control','ADHD'))

data.fixed$ethnicity <-
mapvalues(data.fixed$ethnicity,
from = c('1','2','3','4','5'),
to = c('Caucasian','Asian','Afro-Caribbean','Latino','Mixed'))


data.fixed$eye <-
mapvalues(data.fixed$eye,
from = c('0','1'),
to = c('Right','Left'))

data.fixed$cns_med <-
mapvalues(data.fixed$cns_med,
from = c('0','1'),
to = c('not taken','taken'))

data.fixed$sibling_of_asd <-
mapvalues(data.fixed$sibling_of_asd,
from = c('0','1'),
to = c('No','Yes'))
```

Statistical analyses

First off, the distribution of microvolts in each of the six ERG-related measures in each of the three groups is graphed.

Plots

```
# a library to get nice colour combinations
library(paletteer)
# checking the colour codes from a selected package and colour range
# all colours are here: https://github.com/EmilHvitfeldt/r-color-palettes
paletteer_d("ggsci::lanonc_lancet")
```

```
## <colors>
## #00468BFF #ED0000FF #42B540FF #0099B4FF #925E9FFF #FDAF91FF #AD002AFF #ADB6B6FF #1B1919FF
```

```
picked.colours <-c('#ADB6B6FF','#AD002AFF','#0099B4FF')



boxplot(a20 ~ group,
main='a20',
notch=T, xlab='Group',
ylab=expression(paste('Amplitudes ( ',mu,'V)')),
col=picked.colours,lwd=1, pch=22, data = data.fixed)
```

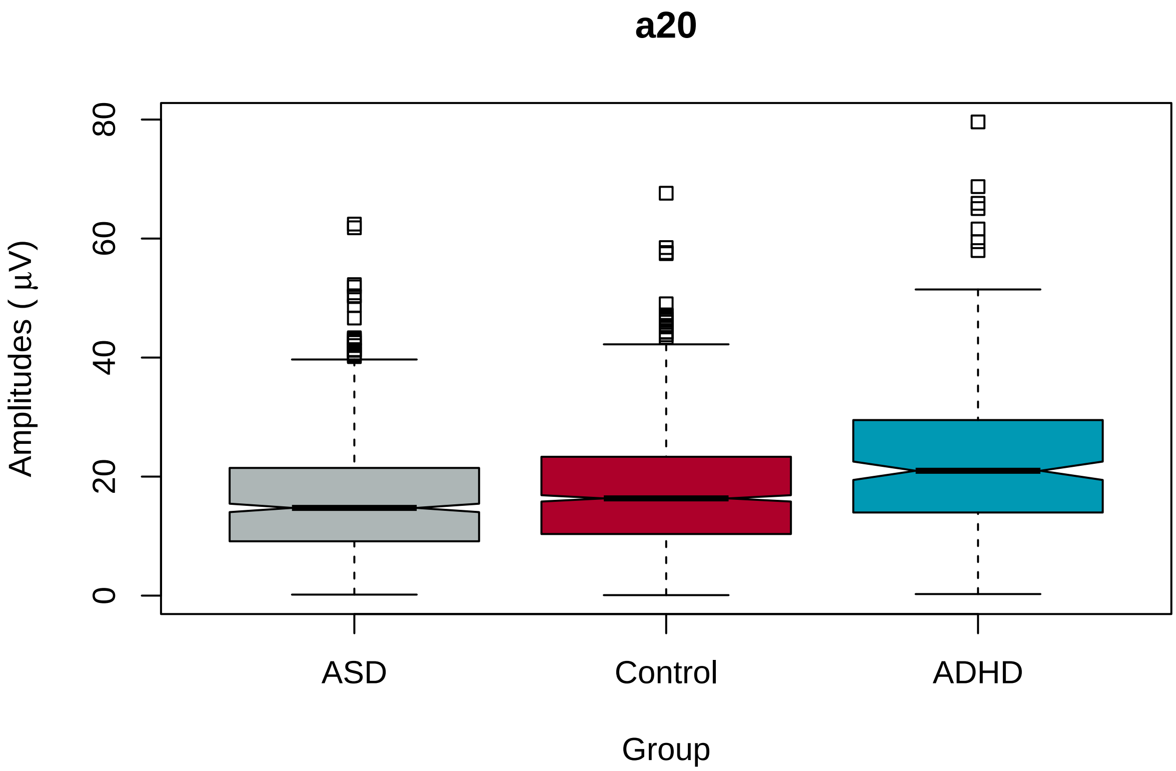


```
boxplot(a40 ~ group,
main='a40',
notch=T, xlab='Group',
ylab=expression(paste('Amplitudes ( ',mu,'V)')),
col=picked.colours,lwd=1, pch=22, data = data.fixed)
```

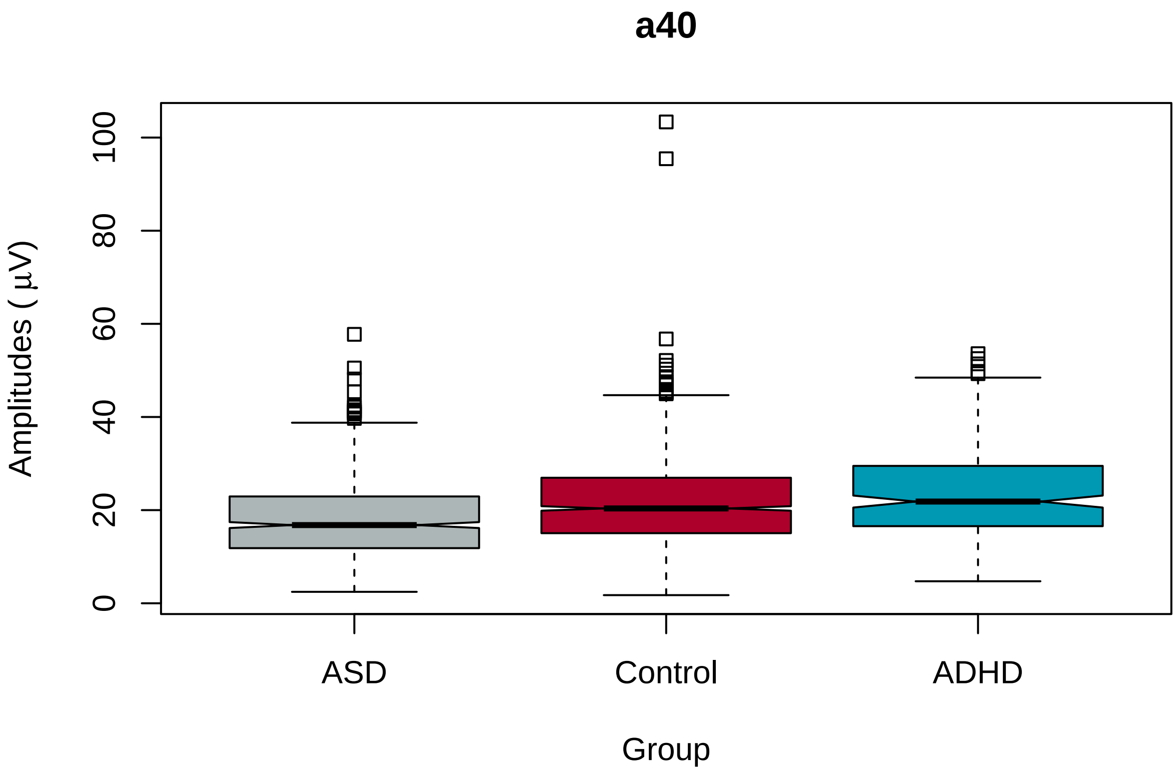


```
boxplot(op80 ~ group,
main='op80',
notch=T, xlab='Group',
ylab=expression(paste('Amplitudes ( ',mu,'V)')),
col=picked.colours,lwd=1, pch=22, data = data.fixed)
```

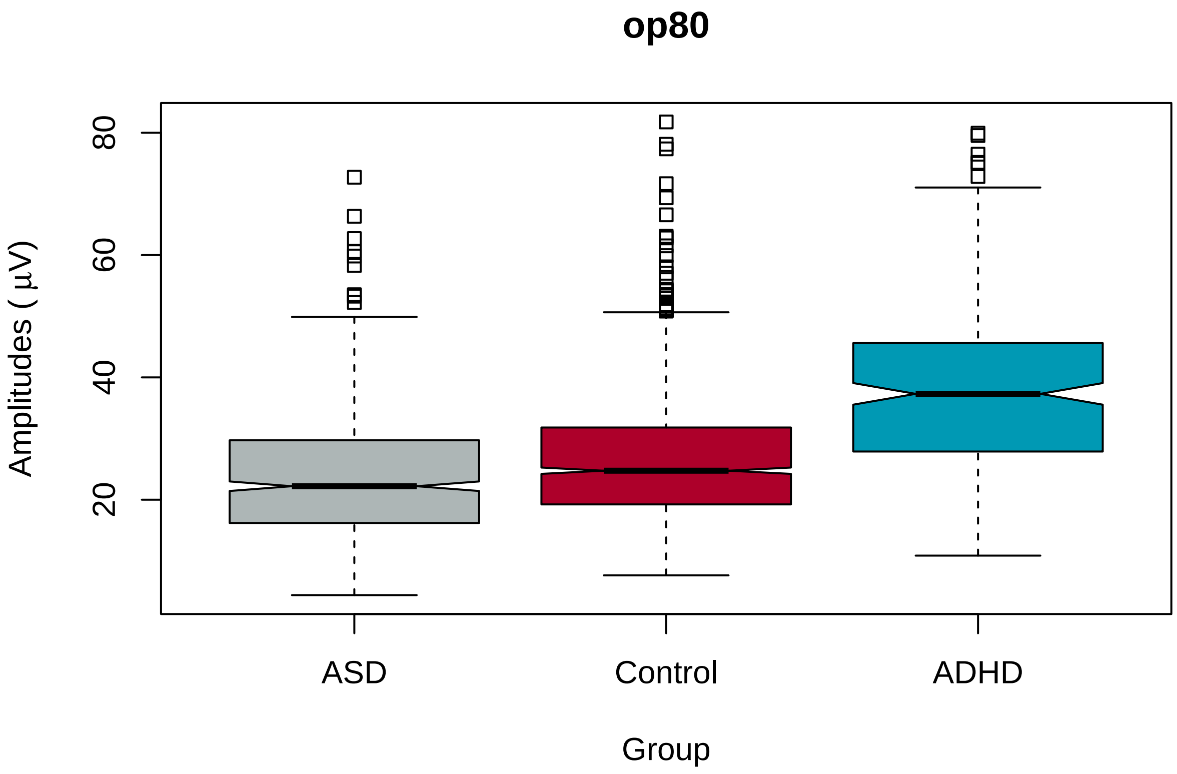


```
boxplot(op160 ~ group,
main='op160',
notch=T, xlab='Group',
ylab=expression(paste('Amplitudes ( ',mu,'V)')),
col=picked.colours,lwd=1, pch=22, data = data.fixed)
```

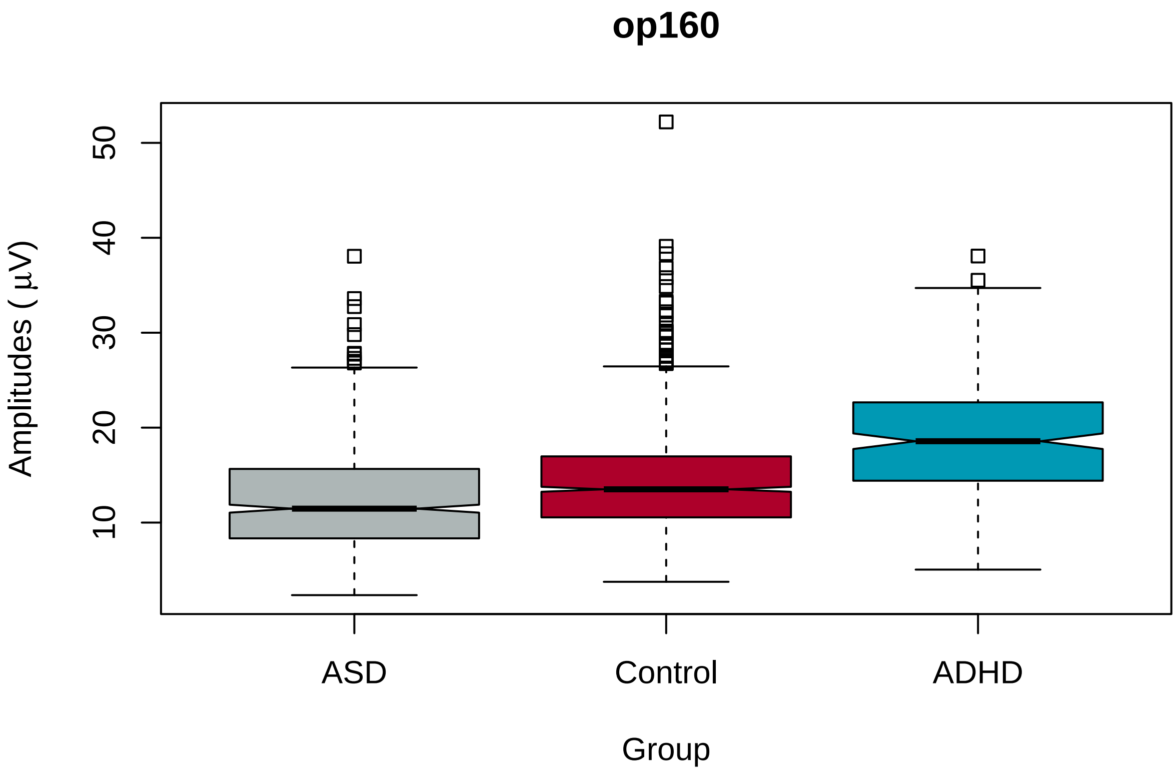


```
boxplot(b20 ~ group,
main='b20',
notch=T, xlab='Group',
ylab=expression(paste('Amplitudes ( ',mu,'V)')),
col=picked.colours,lwd=1, pch=22, data = data.fixed)
```

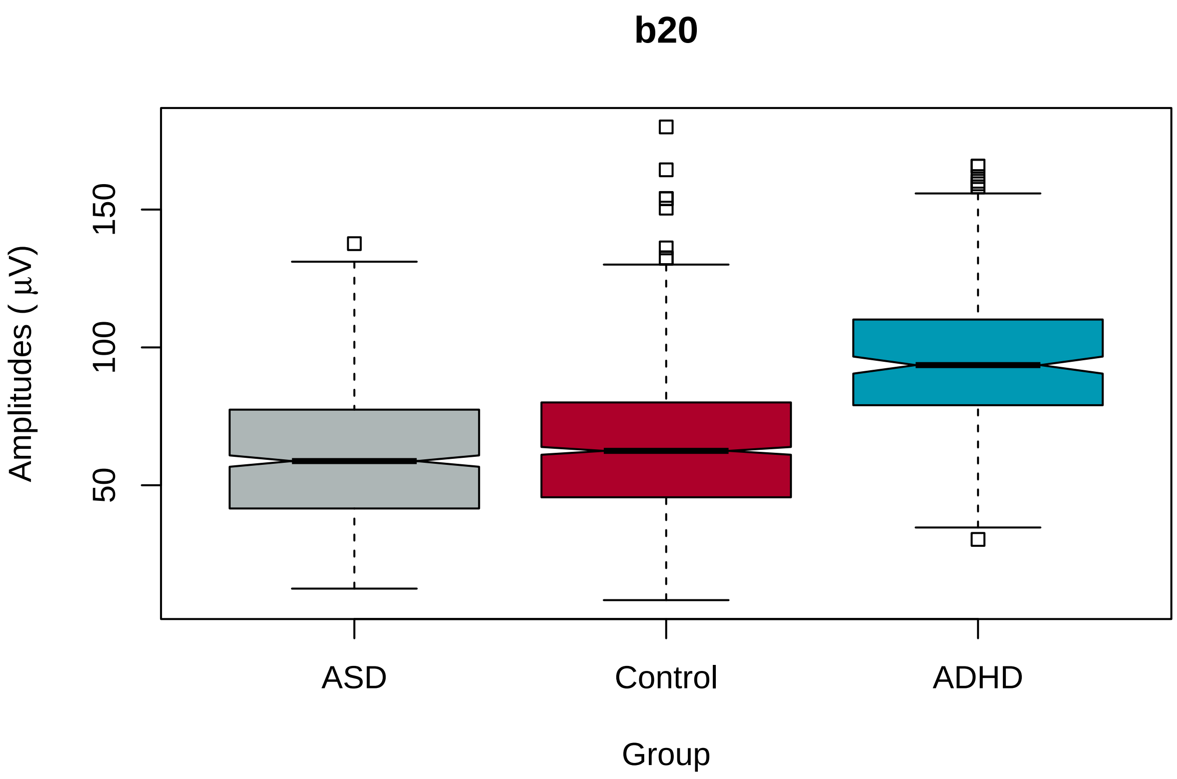


```
boxplot(b40 ~ group,
main='b40',
notch=T, xlab='Group',
ylab=expression(paste('Amplitudes ( ',mu,'V)')),
col=picked.colours,lwd=1, pch=22, data = data.fixed)
```

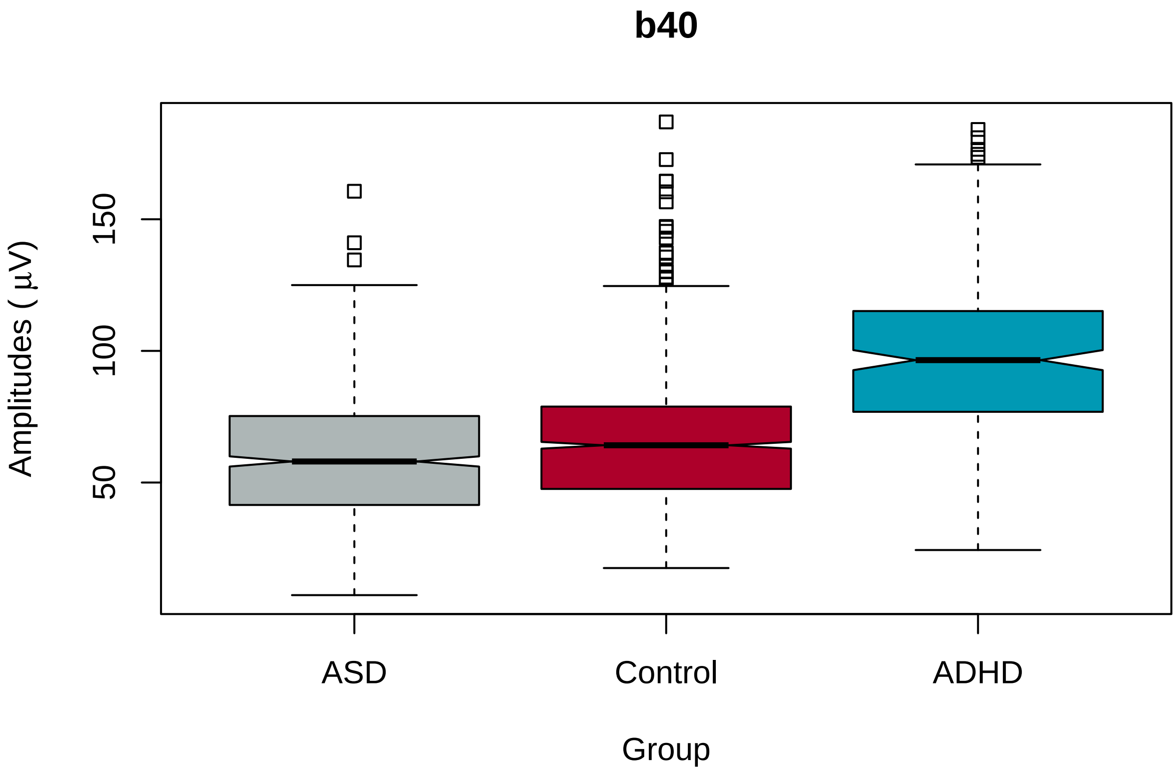


```
boxplot(b_amp ~ group,
main='b_amp',
notch=T, xlab='Group',
ylab=expression(paste('Amplitudes ( ',mu,'V)')),
col=picked.colours,lwd=1, pch=22, data = data.fixed)
```

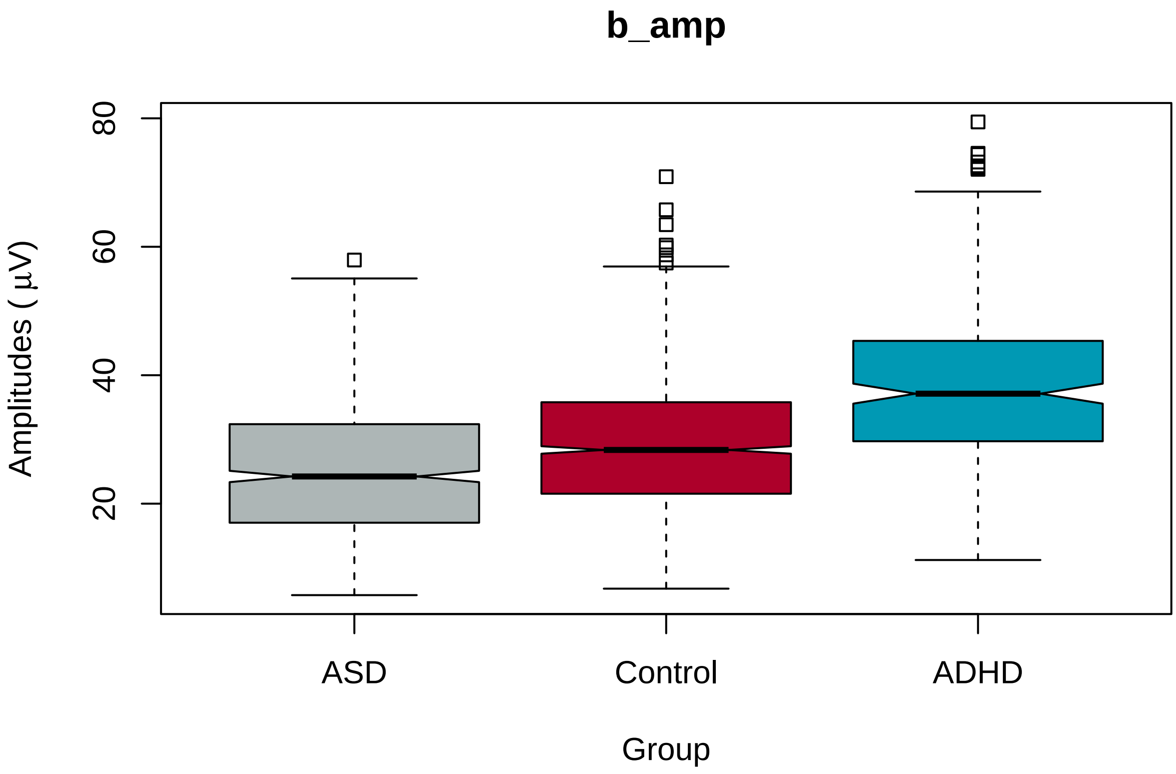


```
boxplot(perc_op_ratio ~ group,
main='OP ratio',
notch=T, xlab='Group',
ylab=expression(paste('Amplitudes ( ',mu,'V)')),
col=picked.colours,lwd=1, pch=22, data = data.fixed)
```

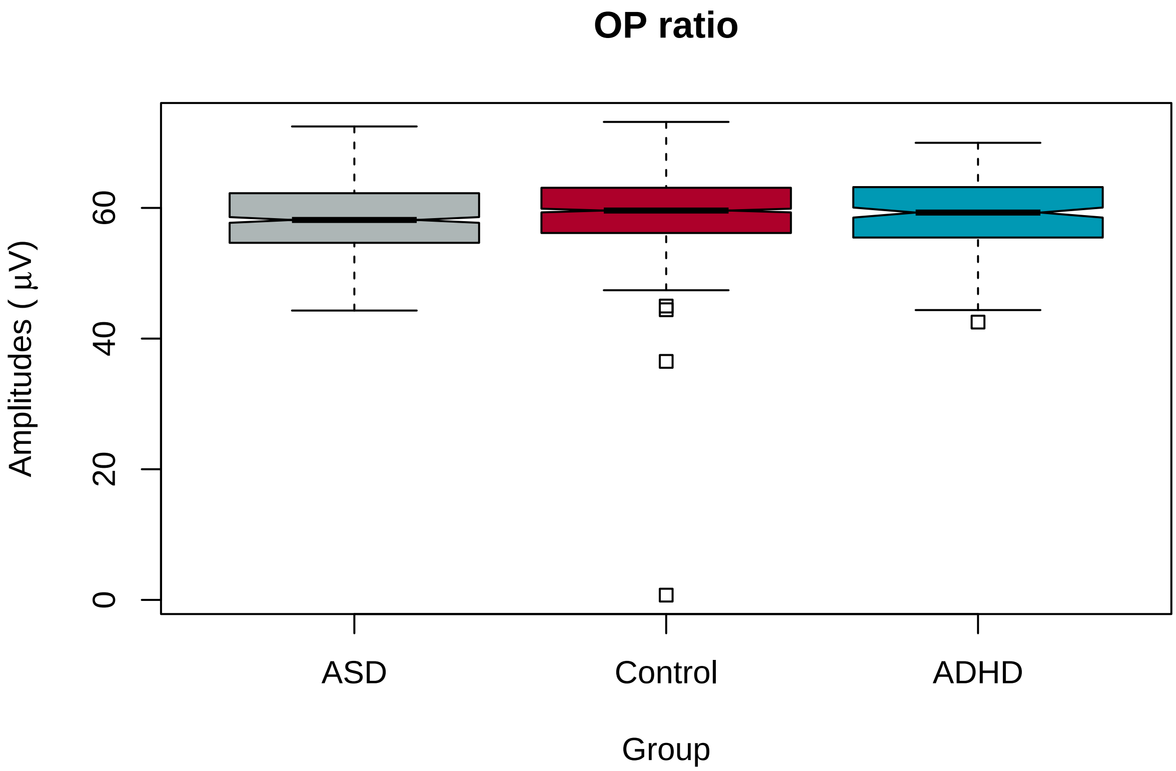

The notches, i.e. approximate $95\% CIs$, indicate large non-overlap between ADHD and control participants in all ERG-related metrics. But the largest differences seems to be for the b20 and b40 metrics (i.e. the 'notches' are farther apart and the Q1-Q3 boxes have less overlap).

Statistical modelling

First off, let's examine the shape of the dependent variables.

```
par(mfrow=c(3,3))
plot(density(data.fixed$a20), main='a20', xlab='microvolts', lwd=2, col='red')
plot(density(data.fixed$a40), main='a40', xlab='microvolts', lwd=2, col='red')
plot(density(data.fixed$op80), main='a80', xlab='microvolts', lwd=2, col='red')
plot(density(data.fixed$op160), main='op160', xlab='microvolts', lwd=2, col='red')
plot(density(data.fixed$b20), main='b20', xlab='microvolts', lwd=2, col='red')
plot(density(data.fixed$b40), main='b40', xlab='microvolts', lwd=2, col='red')
plot(density(data.fixed$b_amp), main='b_amp', xlab='microvolts', lwd=2, col='red')
plot(density(data.fixed$perc_op_ratio), main='OP ratio', xlab='microvolts', lwd=2, col='red')
par(mfrow=c(1,1))
```

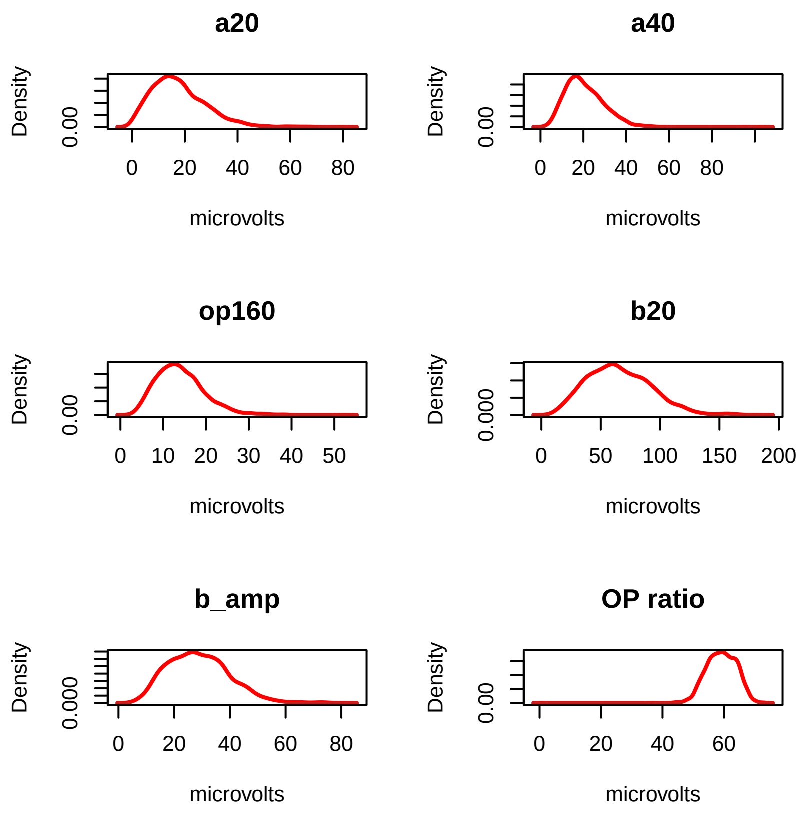

The results indicate a traditional linear regression will give biased results hence reinforcing the idea of a robust approach [1]

We'll use robust (fixed-effects linear regression) for each of the dependent variables.

```
library(robust)
library(knitr)

# a20

a20.model <- lmRob(a20 ~ strength + vert + iris + group + ethnicity
                   + sibling_of_asd
                   + strength:group, data = data.fixed)

anova.a20 <- anova.lmRob(a20.model)

anova.a20 %>% kable()
```

	Chisq Df	RobustF	Pr(F)
(Intercept)	1	NA	NA
strength	4	809.10136	0.0000000
vert	4	97.90334	0.0000000
iris	1	16.99562	0.0000266
group	2	17.75554	0.0000176

[1] Bear in mind that a robust regression is just one of the many possible regression models to use on the data. For example, quantile regression could've been another good option.

	Chisq Df	RobustF	Pr(F)
ethnicity	4	17.21818	0.0000235
sibling_of_asd	1	12.82356	0.0002631
strength:group	8	25.52786	0.0000003

```
# a40

a40.model <- lmRob(a40 ~ strength + vert + iris + group + ethnicity
                   + sibling_of_asd
                   + strength:group, data = data.fixed)

anova.a40 <- anova.lmRob(a40.model)

anova.a40 %>% kable()
```

	Chisq Df	RobustF	Pr(F)
(Intercept)	1	NA	NA
strength	4	907.5899889	0.0000000
vert	4	84.3575568	0.0000000
iris	1	1.6204589	0.1945635
group	2	94.3606550	0.0000000
ethnicity	4	12.5416920	0.0003076
sibling_of_asd	1	0.8826449	0.3383805
strength:group	8	22.7856440	0.0000011

```
# op80

op80.model <- lmRob(op80 ~ strength + vert + iris + group + ethnicity
                   + sibling_of_asd
                   + strength:group, data = data.fixed)

anova.op80 <- anova.lmRob(op80.model)

anova.op80 %>% kable()
```

	Chisq Df	RobustF	Pr(F)
(Intercept)	1	NA	NA
strength	4	813.5519418	0.0000000
vert	4	110.9544888	0.0000000
iris	1	0.1930137	0.6543743
group	2	302.4283727	0.0000000
ethnicity	4	109.7751626	0.0000000
sibling_of_asd	1	22.0924259	0.0000017
strength:group	8	36.0906427	0.0000000

```
# op160

op160.model <- lmRob(op160 ~ strength + vert + iris + group + ethnicity
```

```
                   + sibling_of_asd
                 + strength:group, data = data.fixed)

anova.op160 <- anova.lmRob(op160.model)

anova.op160 %>% kable()
```

	Chisq Df	RobustF	Pr(F)
(Intercept)	1	NA	NA
strength	4	690.594585	0.0000000
vert	4	92.883534	0.0000000
iris	1	3.164952	0.0698495
group	2	298.208674	0.0000000
ethnicity	4	125.734191	0.0000000
sibling_of_asd	1	12.436565	0.0003261
strength:group	8	34.528109	0.0000000

```
# b20

b20.model <- lmRob(b20 ~ strength + vert + iris + group + ethnicity
                   + sibling_of_asd
                   + strength:group, data = data.fixed)

anova.b20 <- anova.lmRob(b20.model)

anova.b20 %>% kable()
```

	Chisq Df	RobustF	Pr(F)
(Intercept)	1	NA	NA
strength	4	893.610595	0.0000000
vert	4	193.563599	0.0000000
iris	1	5.538023	0.0164811
group	2	257.201761	0.0000000
ethnicity	4	29.353102	0.0000000
sibling_of_asd	1	29.229679	0.0000000
strength:group	8	41.450217	0.0000000

```
# b40

b40.model <- lmRob(b40 ~ strength + vert + iris + group + ethnicity
                   + sibling_of_asd
                   + strength:group, data = data.fixed)

anova.b40 <- anova.lmRob(b40.model)

anova.b40 %>% kable()
```

	Chisq Df	RobustF	Pr(F)
(Intercept)	1	NA	NA
strength	4	786.821315	0.0000000
vert	4	141.492142	0.0000000
iris	1	4.249111	0.0356793
group	2	332.011282	0.0000000
ethnicity	4	77.803540	0.0000000
sibling_of_asd	1	24.359045	0.0000005
strength:group	8	29.729267	0.0000000

```
# b_amp

b_amp.model <- lmRob(b_amp ~ strength + vert + iris + group + ethnicity
                      + sibling_of_asd
                    + strength:group, data = data.fixed)

anova.b_amp <- anova.lmRob(b_amp.model)

anova.b_amp %>% kable()
```

	Chisq Df	RobustF	Pr(F)
(Intercept)	1	NA	NA
strength	4	919.0347114	0.0000000
vert	4	133.1917600	0.0000000
iris	1	0.0274722	0.8658741
group	2	269.4054419	0.0000000
ethnicity	4	102.9881576	0.0000000
sibling_of_asd	1	12.5825236	0.0003007
strength:group	8	25.1768145	0.0000003

```
# OPratio

opratio.model <- lmRob(perc_op_ratio ~ strength + vert + iris + group + ethnicity
                        + sibling_of_asd
                    + strength:group, data = data.fixed)

anova.opratio <- anova.lmRob(opratio.model)

anova.opratio %>% kable()
```

	Chisq Df	RobustF	Pr(F)
(Intercept)	1	NA	NA
strength	4	1240.331122	0.0000000
vert	4	24.077025	0.0000006
iris	1	19.659562	0.0000062
group	2	73.138104	0.0000000
ethnicity	4	104.800919	0.0000000
sibling_of_asd	1	1.078053	0.2900318
strength:group	8	21.483117	0.0000023

In all cases, all covariates were significant.

Examining the interaction $FS \cdot G$

Let's commence with some plots

a20

```
boxplot(a20 ~ interaction(group,strength),
        main='Interaction FS•G at a20',
        notch=T, xlab='Flash strength',
        ylab=expression(paste('Microvolts ( ',mu,'V)')),
        xaxt='n',
        col=picked.colours,lwd=1, pch=22, bg=picked.colours,
        data=data.fixed)
# Changing x axis so that the labels for the flash strenght are shown
# underneath the second group for every flash strenght
xtick<-seq(from=2, to=  ,
           by=3,length.out = 5)
axis(side=1, at=xtick, labels = FALSE)
text(x=xtick,  y=-5, # tick location on the Y axis
     labels = c('-.119','.398','.602','.949','1.204'),
# colour for flashes at which pairwise differences existed
# see code below for the actual statistical analyses
     col=c('black'),
     srt = 0, pos = 1, xpd = TRUE)

# a legend
legend('topleft', border='black', bty='n',
       legend=c('ASD','Control', 'ADHD'),
       fill=picked.colours,
       cex=1)
```

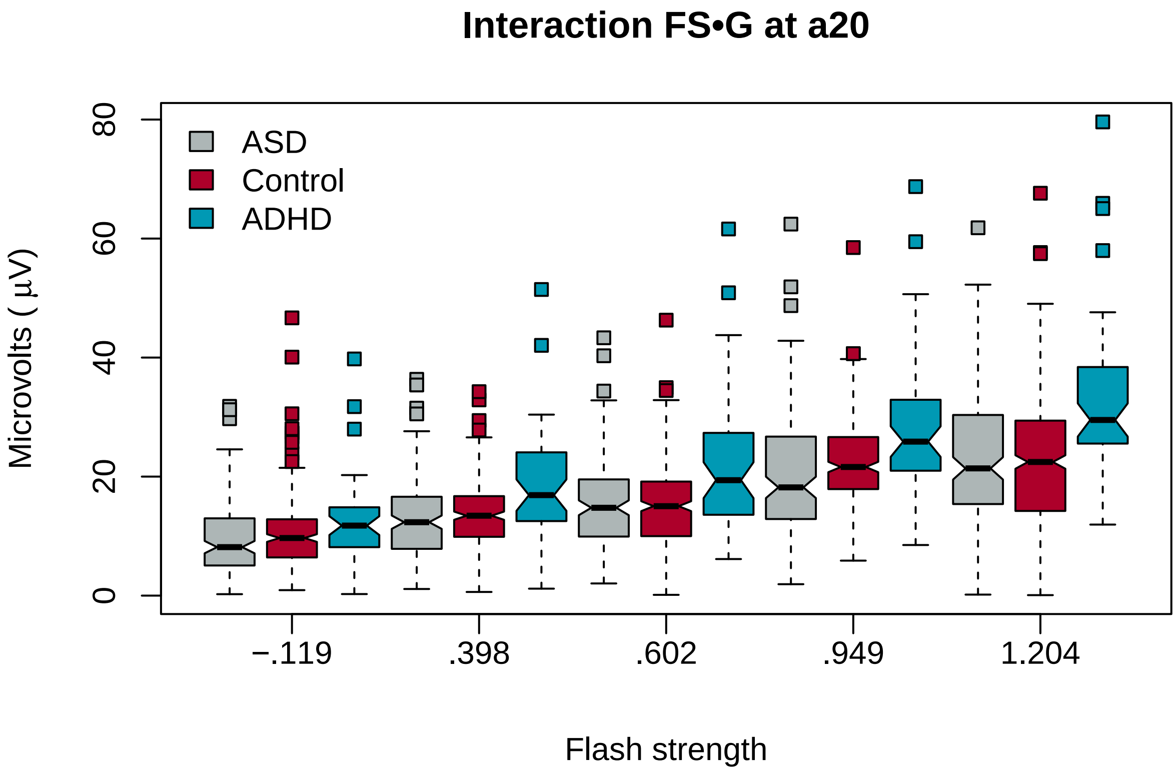

a40

```
boxplot(a40 ~ interaction(group,strength),
        main='Interaction FS•G at a40',
        notch=T, xlab='Flash strength',
        ylab=expression(paste('Microvolts ( ',mu,'V)')),
        xaxt='n',
        col=picked.colours,lwd=1, pch=22, bg=picked.colours,
        data=data.fixed)
# Changing x axis so that the labels for the flash strenght are shown
# underneath the second group for every flash strenght
xtick<-seq(from=2, to=  ,
           by=3,length.out = 5)
axis(side=1, at=xtick, labels = FALSE)
text(x=xtick,  y=-5, # tick location on the Y axis
     labels = c('-.119','.398','.602','.949','1.204'),
# colour for flashes at which pairwise differences existed
# see code below for the actual statistical analyses
     col=c('black'),
     srt = 0, pos = 1, xpd = TRUE)

# a legend
legend('topleft', border='black', bty='n',
       legend=c('ASD','Control', 'ADHD'),
```

```
        fill=picked.colours,
        cex=1)
```

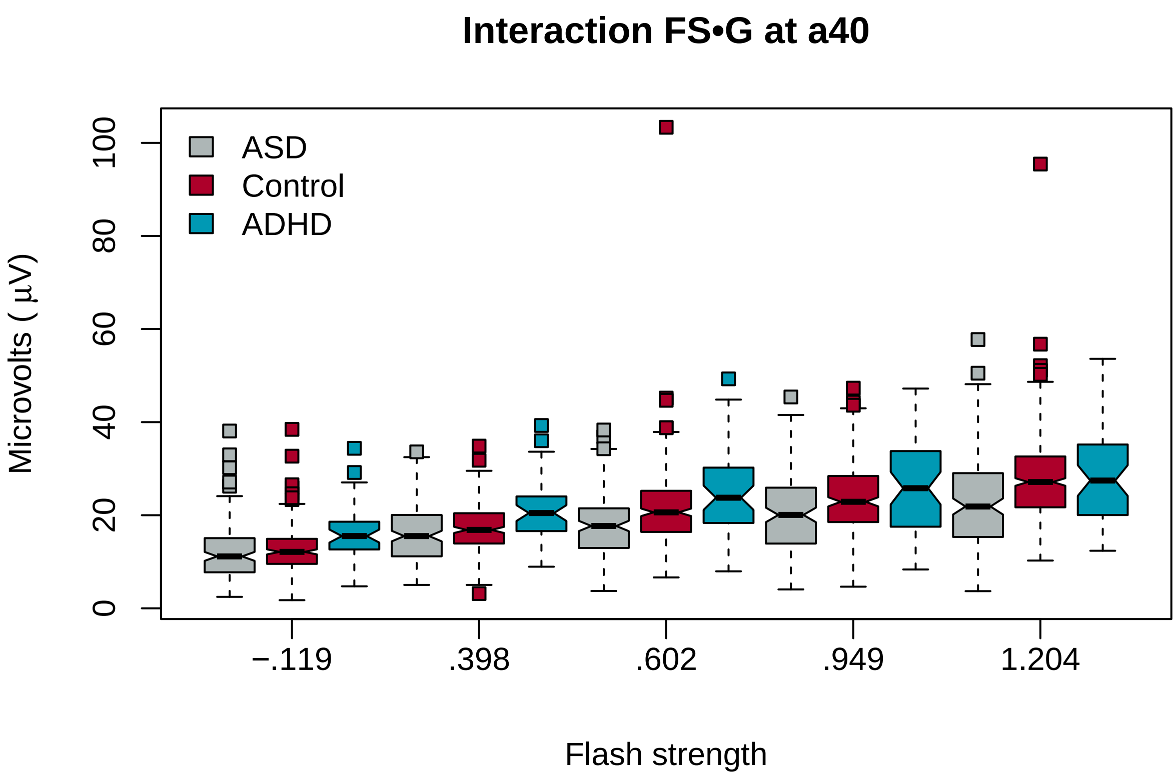

op80

```
boxplot(op80 ~ interaction(group,strength),
        main='Interaction FS•G at op80',
        notch=T, xlab='Flash strength',
        ylab=expression(paste('Microvolts ( ',mu,'V)')),
        xaxt='n',
        col=picked.colours,lwd=1, pch=22, bg=picked.colours,
        data=data.fixed)
# Changing x axis so that the labels for the flash strenght are shown
# underneath the second group for every flash strenght
xtick<-seq(from=2, to=  ,
           by=3,length.out = 5)
axis(side=1, at=xtick, labels = FALSE)
text(x=xtick,  y=-5, # tick location on the Y axis
     labels = c('-.119','.398','.602','.949','1.204'),
# colour for flashes at which pairwise differences existed
# see code below for the actual statistical analyses
     col=c('black'),
     srt = 0, pos = 1, xpd = TRUE)
```

```
# a legend
legend('topleft', border='black', bty='n',
       legend=c('ASD','Control', 'ADHD'),
       fill=picked.colours,
       cex=1)
```

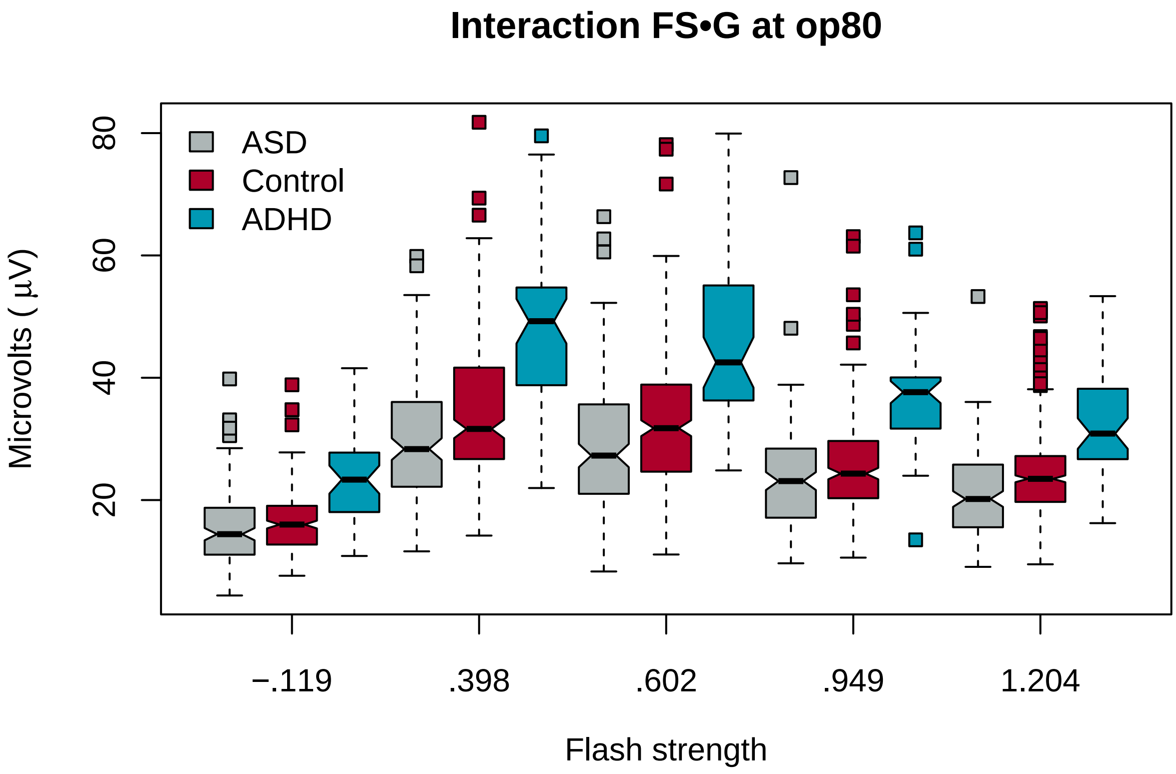

op160

```
boxplot(op160 ~ interaction(group,strength),
        main='Interaction FS•G at op160',
        notch=T, xlab='Flash strength',
        ylab=expression(paste('Microvolts ( ',mu,'V)')),
        xaxt='n',
        col=picked.colours,lwd=1, pch=22, bg=picked.colours,
        data=data.fixed)
# Changing x axis so that the labels for the flash strenght are shown
# underneath the second group for every flash strenght
xtick<-seq(from=2, to=   ,
           by=3,length.out = 5)
axis(side=1, at=xtick, labels = FALSE)
text(x=xtick,  y=-5, # tick location on the Y axis
     labels = c('-.119','.398','.602','.949','1.204'),
# colour for flashes at which pairwise differences existed
```

```
# see code below for the actual statistical analyses
     col=c('black'),
     srt = 0, pos = 1, xpd = TRUE)

# a legend
legend('topleft', border='black', bty='n',
       legend=c('ASD','Control', 'ADHD'),
       fill=picked.colours,
       cex=1)
```

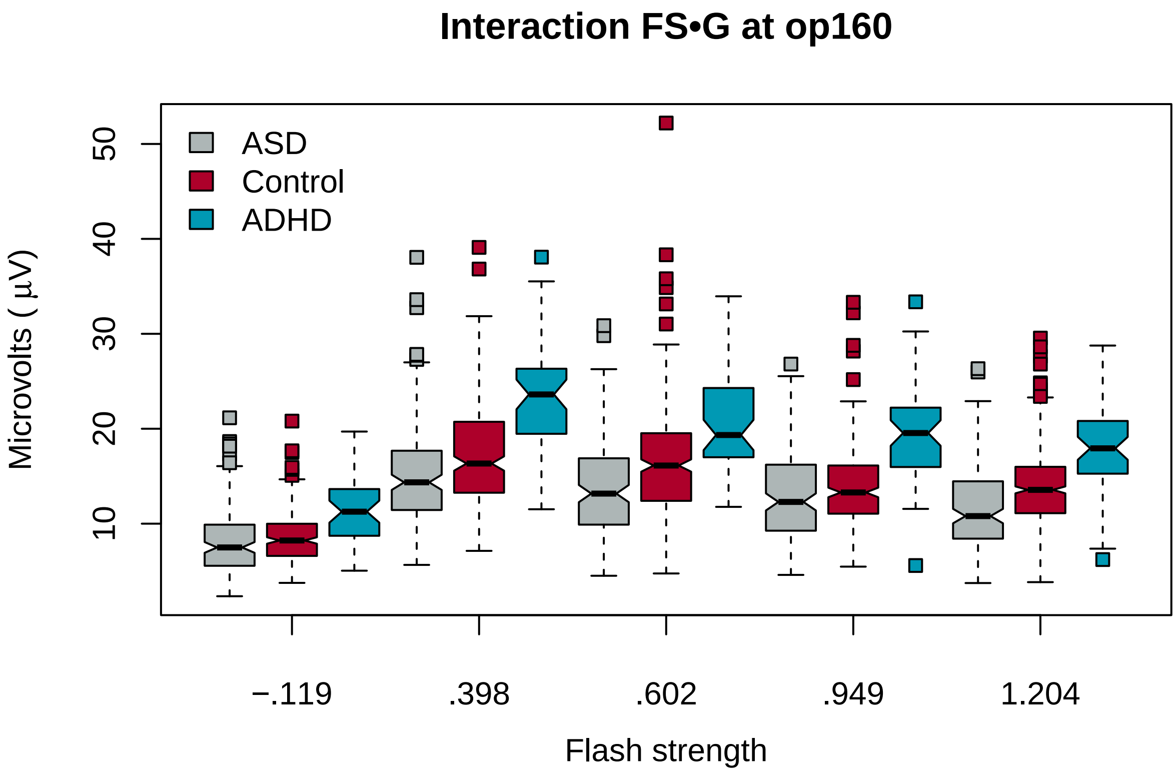

b20

```
boxplot(b20 ~ interaction(group,strength),
        main='Interaction FS•G at b20',
        notch=T, xlab='Flash strength',
        ylab=expression(paste('Microvolts ( ',mu,'V)')),
        xaxt='n',
        col=picked.colours,lwd=1, pch=22, bg=picked.colours,
        data=data.fixed)
# Changing x axis so that the labels for the flash strenght are shown
# underneath the second group for every flash strenght
xtick<-seq(from=2, to=   ,
           by=3,length.out = 5)
```

```
axis(side=1, at=xtick, labels = FALSE)
text(x=xtick,  y=-5, # tick location on the Y axis
     labels = c('-.119','.398','.602','.949','1.204'),
# colour for flashes at which pairwise differences existed
# see code below for the actual statistical analyses
     col=c('black'),
     srt = 0, pos = 1, xpd = TRUE)

# a legend
legend('topleft', border='black', bty='n',
       legend=c('ASD','Control', 'ADHD'),
       fill=picked.colours,
       cex=1)
```

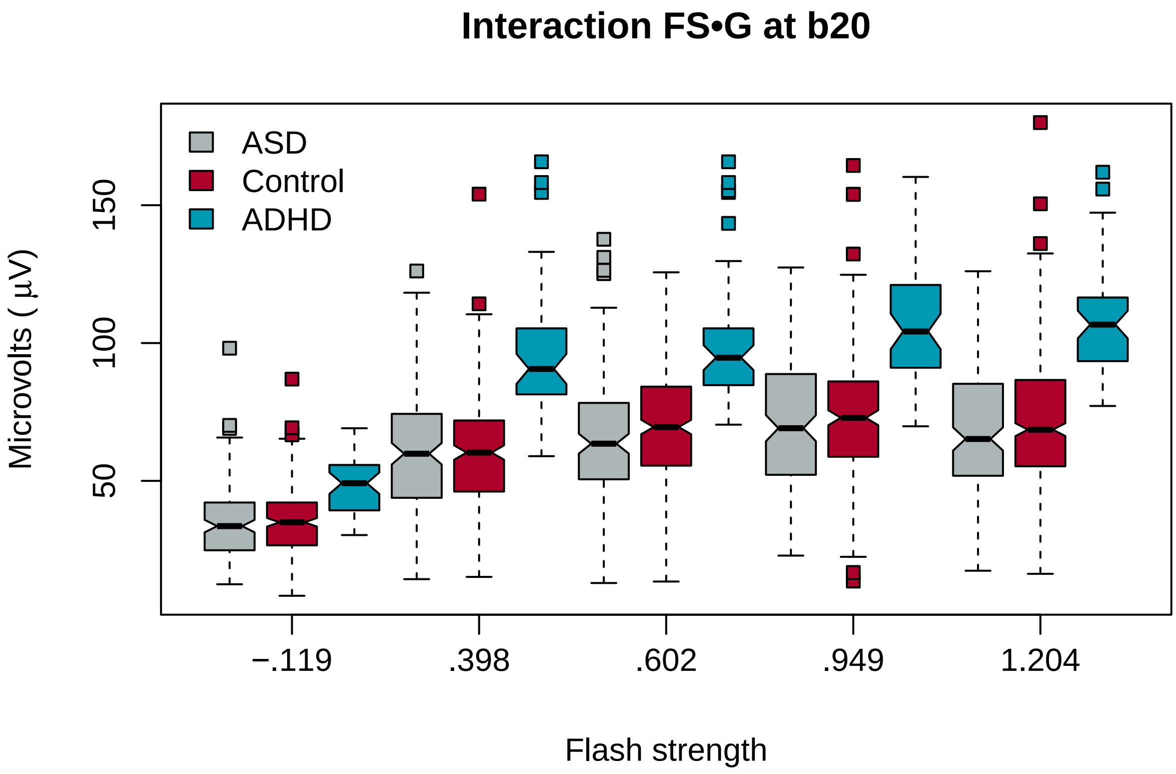

b40

```
boxplot(b40 ~ interaction(group,strength),
        main='Interaction FS•G at b40',
        notch=T, xlab='Flash strength',
        ylab=expression(paste('Microvolts ( ',mu,'V)')),
        xaxt='n',
        col=picked.colours,lwd=1, pch=22, bg=picked.colours,
        data=data.fixed)
```

```
# Changing x axis so that the labels for the flash strenght are shown
# underneath the second group for every flash strenght
xtick<-seq(from=2, to=  ,
           by=3,length.out = 5)
axis(side=1, at=xtick, labels = FALSE)
text(x=xtick,  y=-5, # tick location on the Y axis
     labels = c('-.119','.398','.602','.949','1.204'),
# colour for flashes at which pairwise differences existed
# see code below for the actual statistical analyses
     col=c('black'),
     srt = 0, pos = 1, xpd = TRUE)

# a legend
legend('topleft', border='black', bty='n',
       legend=c('ASD','Control', 'ADHD'),
       fill=picked.colours,
       cex=1)
```

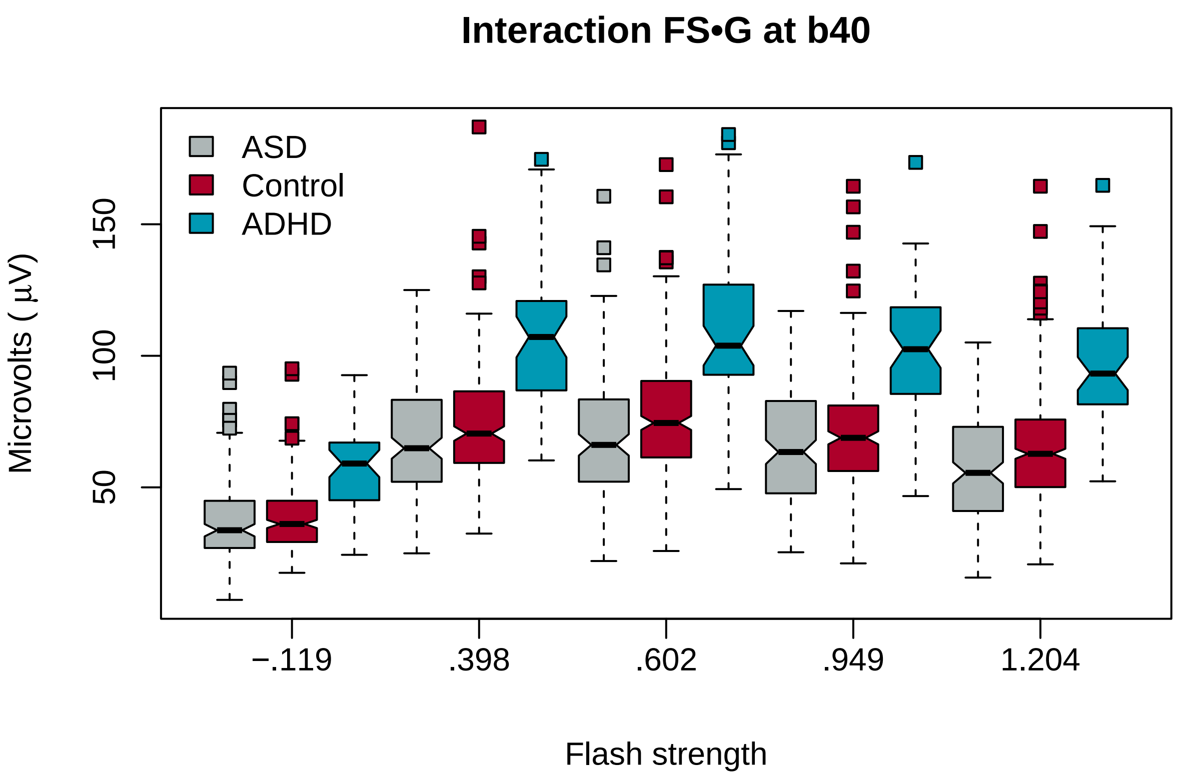

b_amp

```
boxplot(b_amp ~ interaction(group,strength),
        main='Interaction FS•G at b_amp',
        notch=T, xlab='Flash strength',
```

```r
        ylab=expression(paste('Microvolts ( ',mu,'V)')),
        xaxt='n',
        col=picked.colours,lwd=1, pch=22, bg=picked.colours,
        data=data.fixed)
# Changing x axis so that the labels for the flash strenght are shown
# underneath the second group for every flash strenght
xtick<-seq(from=2, to=  ,
           by=3,length.out = 5)
axis(side=1, at=xtick, labels = FALSE)
text(x=xtick,  y=-5, # tick location on the Y axis
     labels = c('-.119','.398','.602','.949','1.204'),
# colour for flashes at which pairwise differences existed
# see code below for the actual statistical analyses
     col=c('black'),
     srt = 0, pos = 1, xpd = TRUE)

# a legend
legend('topleft', border='black', bty='n',
       legend=c('ASD','Control', 'ADHD'),
       fill=picked.colours,
       cex=1)
```

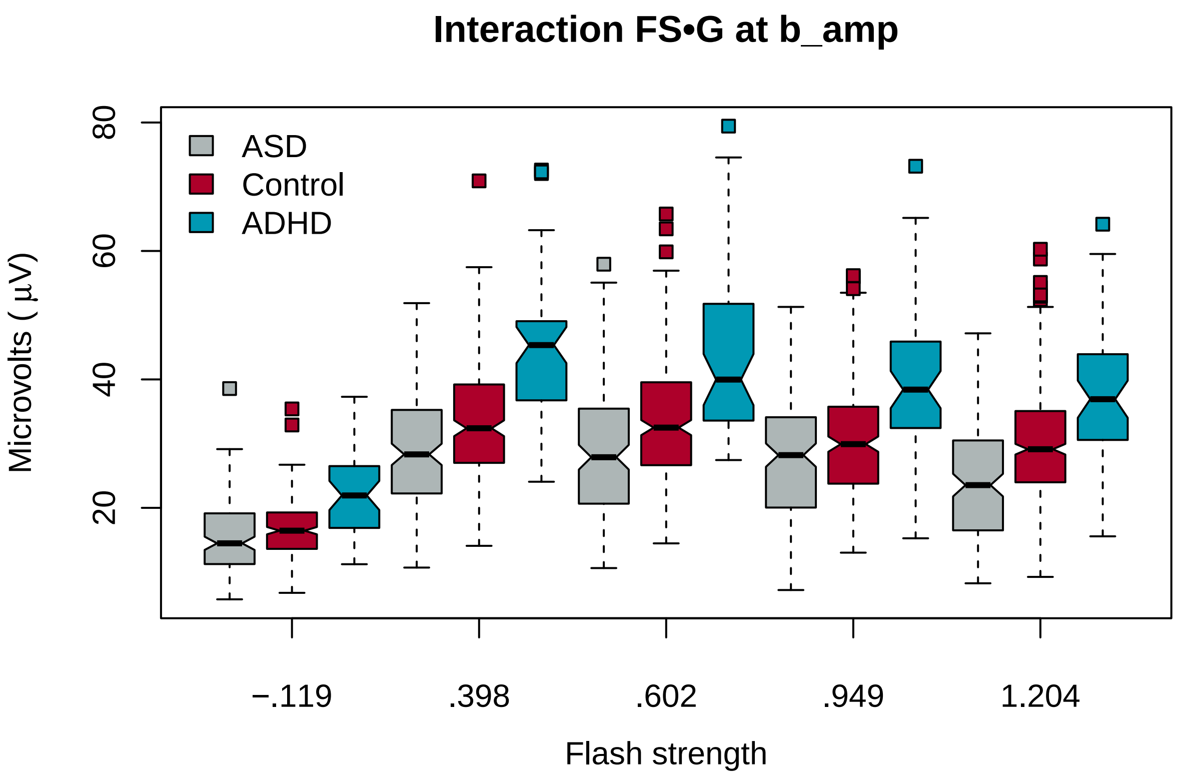

OP_ratio

```r
boxplot(perc_op_ratio ~ interaction(group,strength),
        main='Interaction FS•G at OP ratio',
        notch=T, xlab='Flash strength',
        ylab=expression(paste('Microvolts ( ',mu,'V)')),
        xaxt='n',
        col=picked.colours,lwd=1, pch=22, bg=picked.colours,
        data=data.fixed)
# Changing x axis so that the labels for the flash strenght are shown
# underneath the second group for every flash strenght
xtick<-seq(from=2, to=  ,
           by=3,length.out = 5)
axis(side=1, at=xtick, labels = FALSE)
text(x=xtick,  y=-5, # tick location on the Y axis
     labels = c('-.119','.398','.602','.949','1.204'),
# colour for flashes at which pairwise differences existed
# see code below for the actual statistical analyses
     col=c('black'),
     srt = 0, pos = 1, xpd = TRUE)

# a legend
legend('bottomleft', border='black', bty='n',
       legend=c('ASD','Control', 'ADHD'),
       fill=picked.colours,
       cex=1)
```

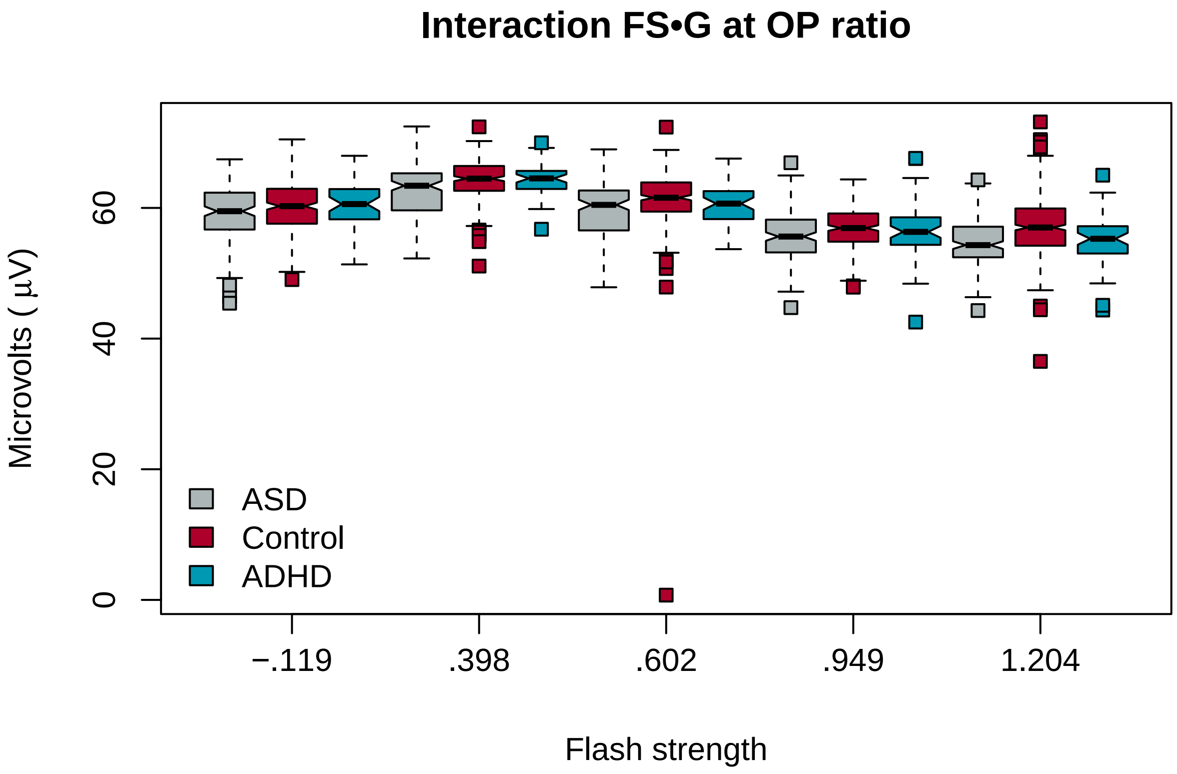

Nonparametric multiple comparisons

In the following section we'll perform pairwise comparisons between the three groups at each flash strength for each ERG-related measure. We'll use the method proposed by Noguchi et al. (2020) [2]. Because we're comparing independent groups, the `mctp` function is used (with the default parameters; i.e. Tukey-type contrast, global pseudo-rank estimation method, Fisher asymptotic approximation method, and with 95% CIs).

a20

```
library(nparcomp)

# creating subsets for each flash strength

flash2 <- subset(data.fixed, strength==-0.119)
flash4 <- subset(data.fixed, strength==0.398)
flash6 <- subset(data.fixed, strength==0.602)
flash8 <- subset(data.fixed, strength==0.949)
flash10 <- subset(data.fixed, strength==1.204)
```

[2] Noguchi, K., Abel, R., Marmolejo-Ramos, F., & Konietschke, F. (2020). Nonparametric multiple comparisons. *Behavior Research Methods, 15* (2), 489-502.

```r
# the tests...
f2 <- mctp(a20 ~ group , info=F, data=flash2)
f4<- mctp(a20 ~ group , info=F, data=flash4)
f6 <- mctp(a20 ~ group , info=F, data=flash6)
f8 <- mctp(a20 ~ group , info=F, data=flash8)
f10 <- mctp(a20 ~ group ,info=F,  data=flash10)
```

flash -0.119

```r
summary(f2)
```

```
##
##  #----------------Nonparametric Multiple Comparisons for relative effects---------------#
##
##  - Alternative Hypothesis:  True differences of relative effects are not equal to 0
##  - Estimation Method: Global Pseudo ranks
##  - Type of Contrast : Tukey
##  - Confidence Level: 95 %
##  - Method = Fisher with 61 DF
##
##  #--------------------------------------------------------------------------------------#
##
##  #----Data Info-------------------------------------------------------------------------#
##    Sample Size    Effect     Lower     Upper
## 1     ASD  146 0.4278430 0.3924215 0.4640199
## 2 Control  247 0.4856381 0.4525847 0.5188176
## 3    ADHD   45 0.5865189 0.5397528 0.6317748
##
##  #----Contrast--------------------------------------------------------------------------#
##        1  2 3
## 2 - 1 -1  1 0
## 3 - 1 -1  0 1
## 3 - 2  0 -1 1
##
##  #----Analysis--------------------------------------------------------------------------#
##       Estimator  Lower Upper Statistic     p.Value
## 2 - 1     0.058 -0.017 0.132     1.845 0.159887924
## 3 - 1     0.159  0.047 0.266     3.390 0.003389865
## 3 - 2     0.101 -0.004 0.204     2.292 0.062782364
##
##  #----Overall---------------------------------------------------------------------------#
##   Quantile     p.Value
## 1 2.389536 0.003389865
##
##  #--------------------------------------------------------------------------------------#
```

flash 0.398

```
summary(f4)
```

```
##
##  #----------------Nonparametric Multiple Comparisons for relative effects---------------#
##
##  - Alternative Hypothesis:  True differences of relative effects are not equal to 0
##  - Estimation Method: Global Pseudo ranks
##  - Type of Contrast : Tukey
##  - Confidence Level: 95 %
##  - Method = Fisher with 62 DF
##
##  #--------------------------------------------------------------------------------------#
##
##  #----Data Info-------------------------------------------------------------------------#
##    Sample Size    Effect     Lower     Upper
## 1     ASD  153 0.4182672 0.3846669 0.4526434
## 2 Control  244 0.4576486 0.4257153 0.4899337
## 3    ADHD   48 0.6240842 0.5781629 0.6678782
##
##  #----Contrast--------------------------------------------------------------------------#
##        1  2 3
## 2 - 1 -1  1 0
## 3 - 1 -1  0 1
## 3 - 2  0 -1 1
##
##  #----Analysis--------------------------------------------------------------------------#
##       Estimator  Lower Upper Statistic      p.Value
## 2 - 1     0.039 -0.031 0.110     1.331 0.3774244023
## 3 - 1     0.206  0.098 0.309     4.506 0.0000857622
## 3 - 2     0.166  0.063 0.266     3.817 0.0007780794
##
##  #----Overall---------------------------------------------------------------------------#
##   Quantile     p.Value
## 1 2.387424 8.57622e-05
##
##  #--------------------------------------------------------------------------------------#
```

flash 0.602

```
summary(f6)
```

```
##
##  #----------------Nonparametric Multiple Comparisons for relative effects---------------#
##
##  - Alternative Hypothesis:  True differences of relative effects are not equal to 0
##  - Estimation Method: Global Pseudo ranks
##  - Type of Contrast : Tukey
##  - Confidence Level: 95 %
##  - Method = Fisher with 66 DF
##
```

```
## #-------------------------------------------------------------------------------------#
##
## #----Data Info-----------------------------------------------------------------------#
##   Sample Size    Effect     Lower     Upper
## 1     ASD  149 0.4565198 0.4225836 0.4908645
## 2 Control  301 0.4365185 0.4061242 0.4673971
## 3    ADHD   52 0.6069618 0.5595096 0.6524771
##
## #----Contrast------------------------------------------------------------------------#
##        1  2 3
## 2 - 1 -1  1 0
## 3 - 1 -1  0 1
## 3 - 2  0 -1 1
##
## #----Analysis------------------------------------------------------------------------#
##       Estimator  Lower Upper Statistic      p.Value
## 2 - 1     -0.02 -0.085 0.045    -0.727 0.7423343362
## 3 - 1      0.15  0.040 0.258     3.216 0.0053952538
## 3 - 2      0.17  0.066 0.271     3.865 0.0007325863
##
## #----Overall-------------------------------------------------------------------------#
##   Quantile      p.Value
## 1 2.376068 0.0007325863
##
## #-------------------------------------------------------------------------------------#
```

flash 0.949

```
summary(f8)
```

```
##
## #----------------Nonparametric Multiple Comparisons for relative effects---------------#
##
##  - Alternative Hypothesis:  True differences of relative effects are not equal to 0
##  - Estimation Method: Global Pseudo ranks
##  - Type of Contrast : Tukey
##  - Confidence Level: 95 %
##  - Method = Fisher with 73 DF
##
## #-------------------------------------------------------------------------------------#
##
## #----Data Info-----------------------------------------------------------------------#
##   Sample Size    Effect     Lower     Upper
## 1     ASD  149 0.3926382 0.3593122 0.4269950
## 2 Control  252 0.4813836 0.4505119 0.5123980
## 3    ADHD   54 0.6259783 0.5848786 0.6653371
##
## #----Contrast------------------------------------------------------------------------#
##        1  2 3
## 2 - 1 -1  1 0
## 3 - 1 -1  0 1
## 3 - 2  0 -1 1
```

```
##
##  #----Analysis--------------------------------------------------------------------------#
##       Estimator Lower Upper Statistic      p.Value
## 2 - 1     0.089 0.015 0.162     2.852 1.502325e-02
## 3 - 1     0.233 0.133 0.329     5.459 1.561463e-06
## 3 - 2     0.145 0.052 0.235     3.698 1.103677e-03
##
##  #----Overall---------------------------------------------------------------------------#
##   Quantile      p.Value
## 1 2.384717 1.561463e-06
##
##  #--------------------------------------------------------------------------------------#
```

flash 1.204

```
summary(f10)
```

```
##
##  #----------------Nonparametric Multiple Comparisons for relative effects---------------#
##
##  - Alternative Hypothesis:  True differences of relative effects are not equal to 0
##  - Estimation Method: Global Pseudo ranks
##  - Type of Contrast : Tukey
##  - Confidence Level: 95 %
##  - Method = Fisher with 71 DF
##
##  #--------------------------------------------------------------------------------------#
##
##  #----Data Info-------------------------------------------------------------------------#
##    Sample Size    Effect     Lower     Upper
## 1     ASD  158 0.4354056 0.4050584 0.4662445
## 2 Control  449 0.4127141 0.3874845 0.4384105
## 3    ADHD   53 0.6518804 0.6141718 0.6877771
##
##  #----Contrast--------------------------------------------------------------------------#
##        1  2 3
## 2 - 1 -1  1 0
## 3 - 1 -1  0 1
## 3 - 2  0 -1 1
##
##  #----Analysis--------------------------------------------------------------------------#
##       Estimator  Lower Upper Statistic      p.Value
## 2 - 1    -0.023 -0.084 0.039    -0.875 6.516903e-01
## 3 - 1     0.216  0.124 0.305     5.491 1.277795e-06
## 3 - 2     0.239  0.157 0.318     6.817 3.421707e-09
##
##  #----Overall---------------------------------------------------------------------------#
##   Quantile      p.Value
## 1  2.37991 3.421707e-09
##
##  #--------------------------------------------------------------------------------------#
```

a40

```r
# the tests...
f2.a40 <- mctp(a40 ~ group , info=F, data=flash2)
f4.a40 <- mctp(a40 ~ group , info=F, data=flash4)
f6.a40 <- mctp(a40 ~ group , info=F, data=flash6)
f8.a40 <- mctp(a40 ~ group , info=F, data=flash8)
f10.a40 <- mctp(a40 ~ group ,info=F,  data=flash10)
```

flash -0.119

```r
summary(f2.a40)
```

```
##
##  #----------------Nonparametric Multiple Comparisons for relative effects---------------#
##
##  - Alternative Hypothesis:  True differences of relative effects are not equal to 0
##  - Estimation Method: Global Pseudo ranks
##  - Type of Contrast : Tukey
##  - Confidence Level: 95 %
##  - Method = Fisher with 64 DF
##
##  #---------------------------------------------------------------------------------------#
##
##  #----Data Info--------------------------------------------------------------------------#
##    Sample Size    Effect     Lower     Upper
## 1     ASD  146 0.4056611 0.3720634 0.4401661
## 2 Control  247 0.4520746 0.4210891 0.4834362
## 3    ADHD   45 0.6422642 0.5992861 0.6830705
##
##  #----Contrast---------------------------------------------------------------------------#
##        1  2 3
## 2 - 1 -1  1 0
## 3 - 1 -1  0 1
## 3 - 2  0 -1 1
##
##  #----Analysis---------------------------------------------------------------------------#
##       Estimator  Lower Upper Statistic      p.Value
## 2 - 1     0.046 -0.027 0.119     1.521 2.839664e-01
## 3 - 1     0.237  0.133 0.335     5.363 3.278778e-06
## 3 - 2     0.190  0.093 0.284     4.649 4.975968e-05
##
##  #----Overall----------------------------------------------------------------------------#
##   Quantile      p.Value
## 1 2.390247 3.278778e-06
##
##  #---------------------------------------------------------------------------------------#
```

flash 0.398

```
summary(f4.a40)
```

```
##
## #----------------Nonparametric Multiple Comparisons for relative effects---------------#
##
## - Alternative Hypothesis: True differences of relative effects are not equal to 0
## - Estimation Method: Global Pseudo ranks
## - Type of Contrast : Tukey
## - Confidence Level: 95 %
## - Method = Fisher with 72 DF
##
## #--------------------------------------------------------------------------------------#
##
## #----Data Info-------------------------------------------------------------------------#
##    Sample Size    Effect     Lower     Upper
## 1     ASD  153 0.3979934 0.3656386 0.4312648
## 2 Control  244 0.4612185 0.4310053 0.4917193
## 3    ADHD   48 0.6407881 0.6007601 0.6789476
##
## #----Contrast--------------------------------------------------------------------------#
##        1  2 3
## 2 - 1 -1  1 0
## 3 - 1 -1  0 1
## 3 - 2  0 -1 1
##
## #----Analysis--------------------------------------------------------------------------#
##       Estimator  Lower Upper Statistic      p.Value
## 2 - 1     0.063 -0.009 0.135     2.086 9.838434e-02
## 3 - 1     0.243  0.146 0.335     5.846 2.450416e-07
## 3 - 2     0.180  0.088 0.268     4.666 3.285298e-05
##
## #----Overall---------------------------------------------------------------------------#
##   Quantile      p.Value
## 1 2.386704 2.450416e-07
##
## #--------------------------------------------------------------------------------------#
```

flash 0.602

```
summary(f6.a40)
```

```
##
## #----------------Nonparametric Multiple Comparisons for relative effects---------------#
##
## - Alternative Hypothesis: True differences of relative effects are not equal to 0
## - Estimation Method: Global Pseudo ranks
## - Type of Contrast : Tukey
## - Confidence Level: 95 %
```

```
##  - Method = Fisher with 61 DF
##
##  #--------------------------------------------------------------------------------------#
##
##  #----Data Info-------------------------------------------------------------------------#
##    Sample Size    Effect     Lower     Upper
## 1     ASD  149 0.3865511 0.3534508 0.4207341
## 2 Control  301 0.5092468 0.4767281 0.5416875
## 3    ADHD   52 0.6042021 0.5536176 0.6526508
##
##  #----Contrast--------------------------------------------------------------------------#
##        1  2 3
## 2 - 1 -1  1 0
## 3 - 1 -1  0 1
## 3 - 2  0 -1 1
##
##  #----Analysis--------------------------------------------------------------------------#
##       Estimator  Lower Upper Statistic      p.Value
## 2 - 1     0.123  0.059 0.185     4.561 9.793291e-05
## 3 - 1     0.218  0.102 0.328     4.421 2.542132e-04
## 3 - 2     0.095 -0.017 0.205     2.019 1.109637e-01
##
##  #----Overall---------------------------------------------------------------------------#
##   Quantile      p.Value
## 1 2.377564 9.793291e-05
##
##  #--------------------------------------------------------------------------------------#
```

flash 0.949

```
summary(f8.a40)
```

```
##
##  #----------------Nonparametric Multiple Comparisons for relative effects---------------#
##
##  - Alternative Hypothesis:  True differences of relative effects are not equal to 0
##  - Estimation Method: Global Pseudo ranks
##  - Type of Contrast : Tukey
##  - Confidence Level: 95 %
##  - Method = Fisher with 67 DF
##
##  #--------------------------------------------------------------------------------------#
##
##  #----Data Info-------------------------------------------------------------------------#
##    Sample Size    Effect     Lower     Upper
## 1     ASD  149 0.4101298 0.3761529 0.4449867
## 2 Control  252 0.5036669 0.4706048 0.5366970
## 3    ADHD   54 0.5862032 0.5386974 0.6321574
##
##  #----Contrast--------------------------------------------------------------------------#
##        1  2 3
## 2 - 1 -1  1 0
```

```
## 3 - 1 -1  0 1
## 3 - 2  0 -1 1
##
##  #----Analysis--------------------------------------------------------------------------#
##       Estimator  Lower Upper Statistic     p.Value
## 2 - 1     0.094  0.023 0.163     3.141 0.006811877
## 3 - 1     0.176  0.065 0.283     3.765 0.001058879
## 3 - 2     0.083 -0.024 0.187     1.840 0.159921362
##
##  #----Overall---------------------------------------------------------------------------#
##   Quantile     p.Value
## 1 2.380195 0.001058879
##
##  #--------------------------------------------------------------------------------------#
```

flash 1.204

```
summary(f10.a40)
```

```
##
##  #----------------Nonparametric Multiple Comparisons for relative effects---------------#
##
##  - Alternative Hypothesis:  True differences of relative effects are not equal to 0
##  - Estimation Method: Global Pseudo ranks
##  - Type of Contrast : Tukey
##  - Confidence Level: 95 %
##  - Method = Fisher with 60 DF
##
##  #--------------------------------------------------------------------------------------#
##
##  #----Data Info-------------------------------------------------------------------------#
##    Sample Size    Effect     Lower     Upper
## 1     ASD  158 0.3973282 0.3648745 0.4307110
## 2 Control  449 0.5496609 0.5184623 0.5804738
## 3    ADHD   53 0.5530109 0.5055178 0.5995558
##
##  #----Contrast--------------------------------------------------------------------------#
##        1  2 3
## 2 - 1 -1  1 0
## 3 - 1 -1  0 1
## 3 - 2  0 -1 1
##
##  #----Analysis--------------------------------------------------------------------------#
##       Estimator  Lower Upper Statistic      p.Value
## 2 - 1     0.152  0.089 0.214     5.693 6.713166e-07
## 3 - 1     0.156  0.045 0.262     3.349 3.552408e-03
## 3 - 2     0.003 -0.102 0.108     0.076 9.967392e-01
##
##  #----Overall---------------------------------------------------------------------------#
##   Quantile      p.Value
## 1 2.381975 6.713166e-07
##
```

```
## #-----------------------------------------------------------------------------------------#
```

op80

```
# the tests...
f2.op80 <- mctp(op80 ~ group , info=F, data=flash2)
f4.op80 <- mctp(op80 ~ group , info=F, data=flash4)
f6.op80 <- mctp(op80 ~ group , info=F, data=flash6)
f8.op80 <- mctp(op80 ~ group , info=F, data=flash8)
f10.op80 <- mctp(op80 ~ group ,info=F,  data=flash10)
```

flash -0.119

```
summary(f2.op80)
```

```
##
## #----------------Nonparametric Multiple Comparisons for relative effects---------------#
##
## - Alternative Hypothesis:  True differences of relative effects are not equal to 0
## - Estimation Method: Global Pseudo ranks
## - Type of Contrast : Tukey
## - Confidence Level: 95 %
## - Method = Fisher with 60 DF
##
## #-----------------------------------------------------------------------------------------#
##
## #----Data Info----------------------------------------------------------------------------#
##   Sample Size    Effect     Lower     Upper
## 1     ASD  146 0.3753899 0.3446279 0.4071918
## 2 Control  247 0.4260173 0.3972638 0.4552800
## 3    ADHD   45 0.6985928 0.6597141 0.7348150
##
## #----Contrast-----------------------------------------------------------------------------#
##        1  2 3
## 2 - 1 -1  1 0
## 3 - 1 -1  0 1
## 3 - 2  0 -1 1
##
## #----Analysis-----------------------------------------------------------------------------#
##       Estimator  Lower Upper Statistic      p.Value
## 2 - 1     0.051 -0.018 0.119     1.759 1.898136e-01
## 3 - 1     0.323  0.229 0.411     7.860 1.293549e-10
## 3 - 2     0.273  0.184 0.357     7.163 1.065613e-09
##
## #----Overall------------------------------------------------------------------------------#
##  Quantile      p.Value
## 1 2.395579 1.293549e-10
##
## #-----------------------------------------------------------------------------------------#
```

flash 0.398

```
summary(f4.op80)
```

```
##
##  #----------------Nonparametric Multiple Comparisons for relative effects---------------#
##
##  - Alternative Hypothesis:  True differences of relative effects are not equal to 0
##  - Estimation Method: Global Pseudo ranks
##  - Type of Contrast : Tukey
##  - Confidence Level: 95 %
##  - Method = Fisher with 74 DF
##
##  #---------------------------------------------------------------------------------------#
##
##  #----Data Info--------------------------------------------------------------------------#
##    Sample Size    Effect     Lower     Upper
## 1     ASD  153 0.3308749 0.3059031 0.3568372
## 2 Control  244 0.4362767 0.4100088 0.4629071
## 3    ADHD   48 0.7328483 0.7002333 0.7631153
##
##  #----Contrast---------------------------------------------------------------------------#
##        1  2 3
## 2 - 1 -1  1 0
## 3 - 1 -1  0 1
## 3 - 2  0 -1 1
##
##  #----Analysis---------------------------------------------------------------------------#
##       Estimator Lower Upper Statistic      p.Value
## 2 - 1     0.105 0.045 0.165     4.161 2.206405e-04
## 3 - 1     0.402 0.326 0.473    11.570 0.000000e+00
## 3 - 2     0.297 0.219 0.370     8.775 1.297851e-13
##
##  #----Overall----------------------------------------------------------------------------#
##   Quantile p.Value
## 1 2.386912       0
##
##  #---------------------------------------------------------------------------------------#
```

flash 0.602

```
summary(f6.op80)
```

```
##
##  #----------------Nonparametric Multiple Comparisons for relative effects---------------#
##
##  - Alternative Hypothesis:  True differences of relative effects are not equal to 0
##  - Estimation Method: Global Pseudo ranks
##  - Type of Contrast : Tukey
##  - Confidence Level: 95 %
```

```
##  - Method = Fisher with 86 DF
##
##  #---------------------------------------------------------------------------------------#
##
##  #----Data Info--------------------------------------------------------------------------#
##    Sample Size    Effect     Lower     Upper
## 1     ASD  149 0.3435211 0.3175079 0.3705085
## 2 Control  301 0.4424622 0.4170605 0.4681685
## 3    ADHD   52 0.7140167 0.6835203 0.7426807
##
##  #----Contrast---------------------------------------------------------------------------#
##        1  2 3
## 2 - 1 -1  1 0
## 3 - 1 -1  0 1
## 3 - 2  0 -1 1
##
##  #----Analysis---------------------------------------------------------------------------#
##       Estimator Lower Upper Statistic      p.Value
## 2 - 1     0.099 0.037 0.161     3.770 8.235002e-04
## 3 - 1     0.370 0.296 0.441    11.028 0.000000e+00
## 3 - 2     0.272 0.200 0.340     8.740 6.916689e-14
##
##  #----Overall----------------------------------------------------------------------------#
##   Quantile p.Value
## 1 2.383019       0
##
##  #---------------------------------------------------------------------------------------#
```

flash 0.949

```
summary(f8.op80)
```

```
##
##  #----------------Nonparametric Multiple Comparisons for relative effects---------------#
##
##  - Alternative Hypothesis:  True differences of relative effects are not equal to 0
##  - Estimation Method: Global Pseudo ranks
##  - Type of Contrast : Tukey
##  - Confidence Level: 95 %
##  - Method = Fisher with 88 DF
##
##  #---------------------------------------------------------------------------------------#
##
##  #----Data Info--------------------------------------------------------------------------#
##    Sample Size    Effect     Lower     Upper
## 1     ASD  149 0.3427110 0.3177750 0.3685466
## 2 Control  252 0.4049605 0.3808015 0.4295889
## 3    ADHD   54 0.7523285 0.7227190 0.7797399
##
##  #----Contrast---------------------------------------------------------------------------#
##        1  2 3
## 2 - 1 -1  1 0
```

```
## 3 - 1 -1  0 1
## 3 - 2  0 -1 1
##
##  #----Analysis--------------------------------------------------------------------------#
##       Estimator Lower Upper Statistic    p.Value
## 2 - 1     0.062 0.003 0.121     2.500 0.03730857
## 3 - 1     0.410 0.338 0.477    12.361 0.00000000
## 3 - 2     0.347 0.278 0.413    11.244 0.00000000
##
##  #----Overall---------------------------------------------------------------------------#
##   Quantile p.Value
## 1 2.380536       0
##
##  #--------------------------------------------------------------------------------------#
```

flash 1.204

```
summary(f10.op80)
```

```
##
##  #----------------Nonparametric Multiple Comparisons for relative effects---------------#
##
##  - Alternative Hypothesis:  True differences of relative effects are not equal to 0
##  - Estimation Method: Global Pseudo ranks
##  - Type of Contrast : Tukey
##  - Confidence Level: 95 %
##  - Method = Fisher with 67 DF
##
##  #--------------------------------------------------------------------------------------#
##
##  #----Data Info-------------------------------------------------------------------------#
##    Sample Size    Effect     Lower     Upper
## 1     ASD  158 0.3320123 0.3069633 0.3580493
## 2 Control  449 0.4480195 0.4231946 0.4731063
## 3    ADHD   53 0.7199682 0.6863990 0.7512462
##
##  #----Contrast--------------------------------------------------------------------------#
##        1  2 3
## 2 - 1 -1  1 0
## 3 - 1 -1  0 1
## 3 - 2  0 -1 1
##
##  #----Analysis--------------------------------------------------------------------------#
##       Estimator Lower Upper Statistic      p.Value
## 2 - 1     0.116 0.059 0.172     4.880 1.480998e-05
## 3 - 1     0.388 0.309 0.462    10.831 0.000000e+00
## 3 - 2     0.272 0.195 0.346     8.177 4.899303e-12
##
##  #----Overall---------------------------------------------------------------------------#
##   Quantile p.Value
## 1 2.386956       0
##
```

```
## #----------------------------------------------------------------------------------------#
```

op160

```
# the tests...
f2.op160 <- mctp(op160 ~ group , info=F, data=flash2)
f4.op160 <- mctp(op160 ~ group , info=F, data=flash4)
f6.op160 <- mctp(op160 ~ group , info=F, data=flash6)
f8.op160 <- mctp(op160 ~ group , info=F, data=flash8)
f10.op160 <- mctp(op160 ~ group ,info=F,  data=flash10)
```

flash -0.119

```
summary(f2.op160)
```

```
##
## #----------------Nonparametric Multiple Comparisons for relative effects---------------#
##
## - Alternative Hypothesis:  True differences of relative effects are not equal to 0
## - Estimation Method: Global Pseudo ranks
## - Type of Contrast : Tukey
## - Confidence Level: 95 %
## - Method = Fisher with 59 DF
##
## #----------------------------------------------------------------------------------------#
##
## #----Data Info-------------------------------------------------------------------------#
##   Sample Size    Effect     Lower     Upper
## 1     ASD  146 0.3883796 0.3563064 0.4214496
## 2 Control  247 0.4457193 0.4150303 0.4768278
## 3    ADHD   45 0.6659011 0.6227662 0.7064288
##
## #----Contrast--------------------------------------------------------------------------#
##        1  2 3
## 2 - 1 -1  1 0
## 3 - 1 -1  0 1
## 3 - 2  0 -1 1
##
## #----Analysis--------------------------------------------------------------------------#
##       Estimator  Lower Upper Statistic      p.Value
## 2 - 1     0.057 -0.012 0.126     1.969 1.261065e-01
## 3 - 1     0.278  0.175 0.374     6.322 5.321652e-08
## 3 - 2     0.220  0.122 0.314     5.303 5.482302e-06
##
## #----Overall---------------------------------------------------------------------------#
##   Quantile      p.Value
## 1 2.392292 5.321652e-08
##
## #----------------------------------------------------------------------------------------#
```

flash 0.398

```
summary(f4.op160)
```

```
##
##  #----------------Nonparametric Multiple Comparisons for relative effects---------------#
##
##  - Alternative Hypothesis:  True differences of relative effects are not equal to 0
##  - Estimation Method: Global Pseudo ranks
##  - Type of Contrast : Tukey
##  - Confidence Level: 95 %
##  - Method = Fisher with 78 DF
##
##  #---------------------------------------------------------------------------------------#
##
##  #----Data Info--------------------------------------------------------------------------#
##    Sample Size    Effect     Lower     Upper
## 1     ASD  153 0.3404549 0.3138127 0.3681454
## 2 Control  244 0.4447865 0.4177816 0.4721215
## 3    ADHD   48 0.7147586 0.6816272 0.7457284
##
##  #----Contrast---------------------------------------------------------------------------#
##        1  2 3
## 2 - 1 -1  1 0
## 3 - 1 -1  0 1
## 3 - 2  0 -1 1
##
##  #----Analysis---------------------------------------------------------------------------#
##       Estimator Lower Upper Statistic      p.Value
## 2 - 1     0.104 0.040 0.168     3.879 6.626515e-04
## 3 - 1     0.374 0.295 0.448    10.521 0.000000e+00
## 3 - 2     0.270 0.192 0.345     7.981 1.676925e-11
##
##  #----Overall----------------------------------------------------------------------------#
##   Quantile p.Value
## 1 2.385587       0
##
##  #---------------------------------------------------------------------------------------#
```

flash 0.602

```
summary(f6.op160)
```

```
##
##  #----------------Nonparametric Multiple Comparisons for relative effects---------------#
##
##  - Alternative Hypothesis:  True differences of relative effects are not equal to 0
##  - Estimation Method: Global Pseudo ranks
##  - Type of Contrast : Tukey
##  - Confidence Level: 95 %
```

```
##  - Method = Fisher with 79 DF
##
##  #--------------------------------------------------------------------------------------#
##
##  #----Data Info-------------------------------------------------------------------------#
##    Sample Size    Effect     Lower     Upper
## 1     ASD  149 0.3411797 0.3138834 0.3695712
## 2 Control  301 0.4768457 0.4495076 0.5043232
## 3    ADHD   52 0.6819746 0.6474372 0.7146200
##
##  #----Contrast--------------------------------------------------------------------------#
##        1  2 3
## 2 - 1 -1  1 0
## 3 - 1 -1  0 1
## 3 - 2  0 -1 1
##
##  #----Analysis--------------------------------------------------------------------------#
##       Estimator Lower Upper Statistic      p.Value
## 2 - 1     0.136 0.072 0.199     5.020 1.155943e-05
## 3 - 1     0.341 0.258 0.418     9.325 3.219647e-15
## 3 - 2     0.205 0.125 0.283     5.995 2.409036e-07
##
##  #----Overall---------------------------------------------------------------------------#
##   Quantile      p.Value
## 1 2.383531 3.219647e-15
##
##  #--------------------------------------------------------------------------------------#
```

flash 0.949

```
summary(f8.op160)
```

```
##
##  #----------------Nonparametric Multiple Comparisons for relative effects---------------#
##
##  - Alternative Hypothesis:  True differences of relative effects are not equal to 0
##  - Estimation Method: Global Pseudo ranks
##  - Type of Contrast : Tukey
##  - Confidence Level: 95 %
##  - Method = Fisher with 79 DF
##
##  #--------------------------------------------------------------------------------------#
##
##  #----Data Info-------------------------------------------------------------------------#
##    Sample Size    Effect     Lower     Upper
## 1     ASD  149 0.3675429 0.3393531 0.3966684
## 2 Control  252 0.4093672 0.3833254 0.4359275
## 3    ADHD   54 0.7230899 0.6889720 0.7547973
##
##  #----Contrast--------------------------------------------------------------------------#
##        1  2 3
## 2 - 1 -1  1 0
```

```
## 3 - 1 -1  0 1
## 3 - 2  0 -1 1
##
##  #----Analysis--------------------------------------------------------------------------#
##       Estimator  Lower Upper Statistic      p.Value
## 2 - 1     0.042 -0.022 0.106     1.557 2.679289e-01
## 3 - 1     0.356  0.272 0.434     9.559 3.219647e-15
## 3 - 2     0.314  0.236 0.387     9.209 5.939693e-14
##
##  #----Overall---------------------------------------------------------------------------#
##   Quantile      p.Value
## 1 2.383723 3.219647e-15
##
##  #--------------------------------------------------------------------------------------#
```

flash 1.204

```
summary(f10.op160)
```

```
##
##  #----------------Nonparametric Multiple Comparisons for relative effects---------------#
##
##  - Alternative Hypothesis:  True differences of relative effects are not equal to 0
##  - Estimation Method: Global Pseudo ranks
##  - Type of Contrast : Tukey
##  - Confidence Level: 95 %
##  - Method = Fisher with 62 DF
##
##  #--------------------------------------------------------------------------------------#
##
##  #----Data Info-------------------------------------------------------------------------#
##    Sample Size    Effect     Lower     Upper
## 1     ASD  158 0.3277221 0.3007688 0.3558614
## 2 Control  449 0.4705170 0.4437864 0.4974178
## 3    ADHD   53 0.7017610 0.6623709 0.7383707
##
##  #----Contrast--------------------------------------------------------------------------#
##        1  2 3
## 2 - 1 -1  1 0
## 3 - 1 -1  0 1
## 3 - 2  0 -1 1
##
##  #----Analysis--------------------------------------------------------------------------#
##       Estimator Lower Upper Statistic      p.Value
## 2 - 1     0.143 0.086 0.199     5.956 2.650677e-07
## 3 - 1     0.374 0.283 0.459     9.148 9.030554e-13
## 3 - 2     0.231 0.143 0.316     6.131 1.371665e-07
##
##  #----Overall---------------------------------------------------------------------------#
##   Quantile      p.Value
## 1 2.386771 9.030554e-13
##
```

```
## #------------------------------------------------------------------------------------------#
```

b20

```r
# the tests...
f2.b20 <- mctp(b20 ~ group , info=F, data=flash2)
f4.b20 <- mctp(b20 ~ group , info=F, data=flash4)
f6.b20 <- mctp(b20 ~ group , info=F, data=flash6)
f8.b20 <- mctp(b20 ~ group , info=F, data=flash8)
f10.b20 <- mctp(b20 ~ group ,info=F,  data=flash10)
```

flash -0.119

```r
summary(f2.b20)
```

```
##
## #----------------Nonparametric Multiple Comparisons for relative effects---------------#
##
## - Alternative Hypothesis:  True differences of relative effects are not equal to 0
## - Estimation Method: Global Pseudo ranks
## - Type of Contrast : Tukey
## - Confidence Level: 95 %
## - Method = Fisher with 93 DF
##
## #------------------------------------------------------------------------------------------#
##
## #----Data Info--------------------------------------------------------------------------#
##   Sample Size    Effect     Lower     Upper
## 1     ASD  146 0.3898767 0.3600662 0.4205334
## 2 Control  247 0.4030554 0.3773204 0.4293355
## 3    ADHD   45 0.7070679 0.6771702 0.7352790
##
## #----Contrast---------------------------------------------------------------------------#
##        1  2 3
## 2 - 1 -1  1 0
## 3 - 1 -1  0 1
## 3 - 2  0 -1 1
##
## #----Analysis---------------------------------------------------------------------------#
##       Estimator  Lower Upper Statistic      p.Value
## 2 - 1     0.013 -0.057 0.083     0.448 8.950551e-01
## 3 - 1     0.317  0.238 0.392     9.110 5.440093e-15
## 3 - 2     0.304  0.236 0.369    10.155 4.440892e-16
##
## #----Overall----------------------------------------------------------------------------#
##   Quantile      p.Value
## 1  2.37953 4.440892e-16
##
## #------------------------------------------------------------------------------------------#
```

flash 0.398

```
summary(f4.b20)
```

```
##
##  #----------------Nonparametric Multiple Comparisons for relative effects---------------#
##
##  - Alternative Hypothesis:  True differences of relative effects are not equal to 0
##  - Estimation Method: Global Pseudo ranks
##  - Type of Contrast : Tukey
##  - Confidence Level: 95 %
##  - Method = Fisher with 121 DF
##
##  #--------------------------------------------------------------------------------------#
##
##  #----Data Info-------------------------------------------------------------------------#
##    Sample Size    Effect     Lower     Upper
## 1     ASD  153 0.3682498 0.3436966 0.3935053
## 2 Control  244 0.3713023 0.3490283 0.3941372
## 3    ADHD   48 0.7604479 0.7375853 0.7819076
##
##  #----Contrast--------------------------------------------------------------------------#
##        1  2 3
## 2 - 1 -1  1 0
## 3 - 1 -1  0 1
## 3 - 2  0 -1 1
##
##  #----Analysis--------------------------------------------------------------------------#
##       Estimator  Lower Upper Statistic  p.Value
## 2 - 1     0.003 -0.058 0.064     0.119 0.992157
## 3 - 1     0.392  0.331 0.450    13.925 0.000000
## 3 - 2     0.389  0.334 0.441    15.434 0.000000
##
##  #----Overall---------------------------------------------------------------------------#
##   Quantile p.Value
## 1 2.371145       0
##
##  #--------------------------------------------------------------------------------------#
```

flash 0.602

```
summary(f6.b20)
```

```
##
##  #----------------Nonparametric Multiple Comparisons for relative effects---------------#
##
##  - Alternative Hypothesis:  True differences of relative effects are not equal to 0
##  - Estimation Method: Global Pseudo ranks
##  - Type of Contrast : Tukey
##  - Confidence Level: 95 %
```

```
##  - Method = Fisher with 173 DF
##
##  #----------------------------------------------------------------------------------------#
##
##  #----Data Info---------------------------------------------------------------------------#
##    Sample Size    Effect     Lower     Upper
## 1     ASD  149 0.3537387 0.3286766 0.3796312
## 2 Control  301 0.4027403 0.3807356 0.4251437
## 3    ADHD   52 0.7435210 0.7223285 0.7636253
##
##  #----Contrast----------------------------------------------------------------------------#
##        1  2 3
## 2 - 1 -1  1 0
## 3 - 1 -1  0 1
## 3 - 2  0 -1 1
##
##  #----Analysis----------------------------------------------------------------------------#
##       Estimator  Lower Upper Statistic   p.Value
## 2 - 1     0.049 -0.013 0.111     1.865 0.1504496
## 3 - 1     0.390  0.330 0.447    14.091 0.0000000
## 3 - 2     0.341  0.290 0.389    14.958 0.0000000
##
##  #----Overall-----------------------------------------------------------------------------#
##   Quantile p.Value
## 1 2.359127       0
##
##  #----------------------------------------------------------------------------------------#
```

flash 0.949

```
summary(f8.b20)
```

```
##
##  #----------------Nonparametric Multiple Comparisons for relative effects---------------#
##
##  - Alternative Hypothesis:  True differences of relative effects are not equal to 0
##  - Estimation Method: Global Pseudo ranks
##  - Type of Contrast : Tukey
##  - Confidence Level: 95 %
##  - Method = Fisher with 127 DF
##
##  #----------------------------------------------------------------------------------------#
##
##  #----Data Info---------------------------------------------------------------------------#
##    Sample Size    Effect     Lower     Upper
## 1     ASD  149 0.3736699 0.3469363 0.4011979
## 2 Control  252 0.3831481 0.3601834 0.4066464
## 3    ADHD   54 0.7431821 0.7188855 0.7660621
##
##  #----Contrast----------------------------------------------------------------------------#
##        1  2 3
## 2 - 1 -1  1 0
```

```
## 3 - 1 -1  0 1
## 3 - 2  0 -1 1
##
##  #----Analysis--------------------------------------------------------------------------#
##       Estimator  Lower Upper Statistic   p.Value
## 2 - 1     0.009 -0.055 0.074     0.349 0.9349251
## 3 - 1     0.370  0.303 0.433    12.172 0.0000000
## 3 - 2     0.360  0.304 0.414    14.137 0.0000000
##
##  #----Overall---------------------------------------------------------------------------#
##   Quantile p.Value
## 1 2.368231       0
##
##  #--------------------------------------------------------------------------------------#
```

flash 1.204

```
summary(f10.b20)
```

```
##
##  #----------------Nonparametric Multiple Comparisons for relative effects---------------#
##
##  - Alternative Hypothesis:  True differences of relative effects are not equal to 0
##  - Estimation Method: Global Pseudo ranks
##  - Type of Contrast : Tukey
##  - Confidence Level: 95 %
##  - Method = Fisher with 161 DF
##
##  #--------------------------------------------------------------------------------------#
##
##  #----Data Info-------------------------------------------------------------------------#
##    Sample Size    Effect     Lower     Upper
## 1     ASD  158 0.3613592 0.3384722 0.3848934
## 2 Control  449 0.3794958 0.3605616 0.3988042
## 3    ADHD   53 0.7591450 0.7406055 0.7767589
##
##  #----Contrast--------------------------------------------------------------------------#
##        1  2 3
## 2 - 1 -1  1 0
## 3 - 1 -1  0 1
## 3 - 2  0 -1 1
##
##  #----Analysis--------------------------------------------------------------------------#
##       Estimator  Lower Upper Statistic   p.Value
## 2 - 1     0.018 -0.037 0.073     0.774 0.7169015
## 3 - 1     0.398  0.344 0.449    15.768 0.0000000
## 3 - 2     0.380  0.337 0.421    19.341 0.0000000
##
##  #----Overall---------------------------------------------------------------------------#
##   Quantile p.Value
## 1 2.357847       0
##
```

```
## #----------------------------------------------------------------------------------------#
```

b40

```
# the tests...
f2.b40 <- mctp(b40 ~ group , info=F, data=flash2)
f4.b40 <- mctp(b40 ~ group , info=F, data=flash4)
f6.b40 <- mctp(b40 ~ group , info=F, data=flash6)
f8.b40 <- mctp(b40 ~ group , info=F, data=flash8)
f10.b40 <- mctp(b40 ~ group ,info=F,  data=flash10)
```

flash -0.119

```
summary(f2.b40)
```

```
##
## #----------------Nonparametric Multiple Comparisons for relative effects---------------#
##
## - Alternative Hypothesis:  True differences of relative effects are not equal to 0
## - Estimation Method: Global Pseudo ranks
## - Type of Contrast : Tukey
## - Confidence Level: 95 %
## - Method = Fisher with 67 DF
##
## #----------------------------------------------------------------------------------------#
##
## #----Data Info-------------------------------------------------------------------------#
##   Sample Size    Effect     Lower     Upper
## 1     ASD  146 0.3758576 0.3462751 0.4063963
## 2 Control  247 0.4054382 0.3788527 0.4325899
## 3    ADHD   45 0.7187042 0.6847341 0.7503485
##
## #----Contrast--------------------------------------------------------------------------#
##        1  2 3
## 2 - 1 -1  1 0
## 3 - 1 -1  0 1
## 3 - 2  0 -1 1
##
## #----Analysis--------------------------------------------------------------------------#
##       Estimator  Lower Upper Statistic      p.Value
## 2 - 1     0.030 -0.038 0.097     1.042 5.515250e-01
## 3 - 1     0.343  0.257 0.423     9.105 4.662937e-14
## 3 - 2     0.313  0.236 0.387     9.259 1.079137e-13
##
## #----Overall---------------------------------------------------------------------------#
##   Quantile      p.Value
## 1 2.393184 4.662937e-14
##
## #----------------------------------------------------------------------------------------#
```

flash 0.398

```
summary(f4.b40)
```

```
##
##  #----------------Nonparametric Multiple Comparisons for relative effects---------------#
##
##  - Alternative Hypothesis:  True differences of relative effects are not equal to 0
##  - Estimation Method: Global Pseudo ranks
##  - Type of Contrast : Tukey
##  - Confidence Level: 95 %
##  - Method = Fisher with 84 DF
##
##  #---------------------------------------------------------------------------------------#
##
##  #----Data Info--------------------------------------------------------------------------#
##    Sample Size    Effect     Lower     Upper
## 1     ASD  153 0.3451820 0.3201228 0.3711319
## 2 Control  244 0.4126576 0.3877816 0.4379878
## 3    ADHD   48 0.7421604 0.7129858 0.7693289
##
##  #----Contrast---------------------------------------------------------------------------#
##        1  2 3
## 2 - 1 -1  1 0
## 3 - 1 -1  0 1
## 3 - 2  0 -1 1
##
##  #----Analysis---------------------------------------------------------------------------#
##       Estimator Lower Upper Statistic    p.Value
## 2 - 1     0.067 0.006 0.128     2.629 0.02717162
## 3 - 1     0.397 0.326 0.464    12.241 0.00000000
## 3 - 2     0.330 0.260 0.396    10.700 0.00000000
##
##  #----Overall----------------------------------------------------------------------------#
##   Quantile p.Value
## 1 2.384396       0
##
##  #---------------------------------------------------------------------------------------#
```

flash 0.602

```
summary(f6.b40)
```

```
##
##  #----------------Nonparametric Multiple Comparisons for relative effects---------------#
##
##  - Alternative Hypothesis:  True differences of relative effects are not equal to 0
##  - Estimation Method: Global Pseudo ranks
##  - Type of Contrast : Tukey
##  - Confidence Level: 95 %
```

```
##  - Method = Fisher with 94 DF
##
##  #------------------------------------------------------------------------------------#
##
##  #----Data Info-----------------------------------------------------------------------#
##    Sample Size    Effect     Lower     Upper
## 1     ASD  149 0.3370141 0.3123199 0.3626311
## 2 Control  301 0.4211621 0.3977053 0.4449803
## 3    ADHD   52 0.7418238 0.7148692 0.7670606
##
##  #----Contrast------------------------------------------------------------------------#
##        1  2 3
## 2 - 1 -1  1 0
## 3 - 1 -1  0 1
## 3 - 2  0 -1 1
##
##  #----Analysis------------------------------------------------------------------------#
##       Estimator Lower Upper Statistic     p.Value
## 2 - 1     0.084 0.024 0.144     3.336 0.003519335
## 3 - 1     0.405 0.337 0.469    12.976 0.000000000
## 3 - 2     0.321 0.257 0.381    11.408 0.000000000
##
##  #----Overall-------------------------------------------------------------------------#
##   Quantile p.Value
## 1 2.380141       0
##
##  #------------------------------------------------------------------------------------#
```

flash 0.949

```
summary(f8.b40)
```

```
##
##  #----------------Nonparametric Multiple Comparisons for relative effects---------------#
##
##  - Alternative Hypothesis:  True differences of relative effects are not equal to 0
##  - Estimation Method: Global Pseudo ranks
##  - Type of Contrast : Tukey
##  - Confidence Level: 95 %
##  - Method = Fisher with 107 DF
##
##  #------------------------------------------------------------------------------------#
##
##  #----Data Info-----------------------------------------------------------------------#
##    Sample Size    Effect     Lower     Upper
## 1     ASD  149 0.3577524 0.3324188 0.3839065
## 2 Control  252 0.3866123 0.3636850 0.4100535
## 3    ADHD   54 0.7556353 0.7302827 0.7793249
##
##  #----Contrast------------------------------------------------------------------------#
##        1  2 3
## 2 - 1 -1  1 0
```

```
## 3 - 1 -1  0 1
## 3 - 2  0 -1 1
##
##  #----Analysis--------------------------------------------------------------------------#
##       Estimator  Lower Upper Statistic   p.Value
## 2 - 1     0.029 -0.032 0.090     1.118 0.5048986
## 3 - 1     0.398  0.332 0.460    13.061 0.0000000
## 3 - 2     0.369  0.310 0.426    13.689 0.0000000
##
##  #----Overall---------------------------------------------------------------------------#
##   Quantile p.Value
## 1 2.375534       0
##
##  #--------------------------------------------------------------------------------------#
```

flash 1.204

```
summary(f10.b40)
```

```
##
##  #----------------Nonparametric Multiple Comparisons for relative effects---------------#
##
##  - Alternative Hypothesis:  True differences of relative effects are not equal to 0
##  - Estimation Method: Global Pseudo ranks
##  - Type of Contrast : Tukey
##  - Confidence Level: 95 %
##  - Method = Fisher with 75 DF
##
##  #--------------------------------------------------------------------------------------#
##
##  #----Data Info-------------------------------------------------------------------------#
##    Sample Size    Effect     Lower     Upper
## 1     ASD  158 0.3433153 0.3197979 0.3676274
## 2 Control  449 0.4136790 0.3912827 0.4364381
## 3    ADHD   53 0.7430058 0.7145051 0.7695788
##
##  #----Contrast--------------------------------------------------------------------------#
##        1  2 3
## 2 - 1 -1  1 0
## 3 - 1 -1  0 1
## 3 - 2  0 -1 1
##
##  #----Analysis--------------------------------------------------------------------------#
##       Estimator Lower Upper Statistic     p.Value
## 2 - 1     0.070 0.016 0.125     3.075 0.008047543
## 3 - 1     0.400 0.330 0.465    12.608 0.000000000
## 3 - 2     0.329 0.264 0.392    11.313 0.000000000
##
##  #----Overall---------------------------------------------------------------------------#
##   Quantile p.Value
## 1 2.386561       0
##
```

```
## #------------------------------------------------------------------------------------#
```

b_amp

```
# the tests...
f2.b_amp <- mctp(b_amp ~ group , info=F, data=flash2)
f4.b_amp <- mctp(b_amp ~ group , info=F, data=flash4)
f6.b_amp <- mctp(b_amp ~ group , info=F, data=flash6)
f8.b_amp <- mctp(b_amp ~ group , info=F, data=flash8)
f10.b_amp <- mctp(b_amp ~ group ,info=F,  data=flash10)
```

flash -0.119

```
summary(f2.b_amp)
```

```
##
## #----------------Nonparametric Multiple Comparisons for relative effects---------------#
##
## - Alternative Hypothesis:  True differences of relative effects are not equal to 0
## - Estimation Method: Global Pseudo ranks
## - Type of Contrast : Tukey
## - Confidence Level: 95 %
## - Method = Fisher with 54 DF
##
## #------------------------------------------------------------------------------------#
##
## #----Data Info-----------------------------------------------------------------------#
##   Sample Size    Effect     Lower     Upper
## 1     ASD  146 0.3739777 0.3424182 0.4066472
## 2 Control  247 0.4419272 0.4107642 0.4735543
## 3    ADHD   45 0.6840951 0.6392287 0.7257754
##
## #----Contrast------------------------------------------------------------------------#
##        1  2 3
## 2 - 1 -1  1 0
## 3 - 1 -1  0 1
## 3 - 2  0 -1 1
##
## #----Analysis------------------------------------------------------------------------#
##       Estimator Lower Upper Statistic      p.Value
## 2 - 1     0.068 0.000 0.135     2.395 5.035659e-02
## 3 - 1     0.310 0.206 0.408     6.853 2.442058e-08
## 3 - 2     0.242 0.140 0.339     5.579 1.635821e-06
##
## #----Overall-------------------------------------------------------------------------#
##   Quantile      p.Value
## 1 2.395192 2.442058e-08
##
## #------------------------------------------------------------------------------------#
```

flash 0.398

```
summary(f4.b_amp)
```

```
##
##  #----------------Nonparametric Multiple Comparisons for relative effects---------------#
##
##  - Alternative Hypothesis:  True differences of relative effects are not equal to 0
##  - Estimation Method: Global Pseudo ranks
##  - Type of Contrast : Tukey
##  - Confidence Level: 95 %
##  - Method = Fisher with 69 DF
##
##  #---------------------------------------------------------------------------------------#
##
##  #----Data Info--------------------------------------------------------------------------#
##    Sample Size    Effect     Lower     Upper
## 1     ASD  153 0.3385485 0.3120263 0.3661254
## 2 Control  244 0.4497828 0.4218653 0.4780202
## 3    ADHD   48 0.7116686 0.6750601 0.7457065
##
##  #----Contrast---------------------------------------------------------------------------#
##        1  2 3
## 2 - 1 -1  1 0
## 3 - 1 -1  0 1
## 3 - 2  0 -1 1
##
##  #----Analysis---------------------------------------------------------------------------#
##       Estimator Lower Upper Statistic      p.Value
## 2 - 1     0.111 0.049 0.172     4.283 1.932255e-04
## 3 - 1     0.373 0.289 0.452     9.848 1.110223e-15
## 3 - 2     0.262 0.176 0.344     7.099 1.223457e-09
##
##  #----Overall----------------------------------------------------------------------------#
##   Quantile      p.Value
## 1 2.387378 1.110223e-15
##
##  #---------------------------------------------------------------------------------------#
```

flash 0.602

```
summary(f6.b_amp)
```

```
##
##  #----------------Nonparametric Multiple Comparisons for relative effects---------------#
##
##  - Alternative Hypothesis:  True differences of relative effects are not equal to 0
##  - Estimation Method: Global Pseudo ranks
##  - Type of Contrast : Tukey
##  - Confidence Level: 95 %
```

```
##  - Method = Fisher with 89 DF
##
##  #--------------------------------------------------------------------------------------#
##
##  #----Data Info-------------------------------------------------------------------------#
##    Sample Size    Effect     Lower     Upper
## 1     ASD  149 0.3379786 0.3114450 0.3655724
## 2 Control  301 0.4641895 0.4382332 0.4903413
## 3    ADHD   52 0.6978319 0.6670701 0.7269150
##
##  #----Contrast--------------------------------------------------------------------------#
##        1  2 3
## 2 - 1 -1  1 0
## 3 - 1 -1  0 1
## 3 - 2  0 -1 1
##
##  #----Analysis--------------------------------------------------------------------------#
##       Estimator Lower Upper Statistic      p.Value
## 2 - 1     0.126 0.062 0.189     4.675 2.525727e-05
## 3 - 1     0.360 0.284 0.431    10.617 0.000000e+00
## 3 - 2     0.234 0.161 0.304     7.524 1.989425e-10
##
##  #----Overall---------------------------------------------------------------------------#
##   Quantile p.Value
## 1  2.38144       0
##
##  #--------------------------------------------------------------------------------------#
```

flash 0.949

```
summary(f8.b_amp)
```

```
##
##  #----------------Nonparametric Multiple Comparisons for relative effects---------------#
##
##  - Alternative Hypothesis:  True differences of relative effects are not equal to 0
##  - Estimation Method: Global Pseudo ranks
##  - Type of Contrast : Tukey
##  - Confidence Level: 95 %
##  - Method = Fisher with 86 DF
##
##  #--------------------------------------------------------------------------------------#
##
##  #----Data Info-------------------------------------------------------------------------#
##    Sample Size   Effect     Lower     Upper
## 1     ASD  149 0.367426 0.3391076 0.3966897
## 2 Control  252 0.433109 0.4060097 0.4606144
## 3    ADHD   54 0.699465 0.6648557 0.7319413
##
##  #----Contrast--------------------------------------------------------------------------#
##        1  2 3
## 2 - 1 -1  1 0
```

```
## 3 - 1 -1  0 1
## 3 - 2  0 -1 1
##
##  #----Analysis--------------------------------------------------------------------------#
##       Estimator Lower Upper Statistic      p.Value
## 2 - 1     0.066 0.000 0.130     2.393 4.849338e-02
## 3 - 1     0.332 0.248 0.411     8.971 2.065015e-14
## 3 - 2     0.266 0.187 0.343     7.721 4.120637e-11
##
##  #----Overall---------------------------------------------------------------------------#
##   Quantile      p.Value
## 1 2.381265 2.065015e-14
##
##  #--------------------------------------------------------------------------------------#
```

flash 1.204

```
summary(f10.b_amp)
```

```
##
##  #----------------Nonparametric Multiple Comparisons for relative effects---------------#
##
##  - Alternative Hypothesis:  True differences of relative effects are not equal to 0
##  - Estimation Method: Global Pseudo ranks
##  - Type of Contrast : Tukey
##  - Confidence Level: 95 %
##  - Method = Fisher with 64 DF
##
##  #--------------------------------------------------------------------------------------#
##
##  #----Data Info-------------------------------------------------------------------------#
##    Sample Size    Effect     Lower     Upper
## 1     ASD  158 0.3314163 0.3050579 0.3588760
## 2 Control  449 0.4893535 0.4622791 0.5164905
## 3    ADHD   53 0.6792302 0.6411804 0.7150384
##
##  #----Contrast--------------------------------------------------------------------------#
##        1  2 3
## 2 - 1 -1  1 0
## 3 - 1 -1  0 1
## 3 - 2  0 -1 1
##
##  #----Analysis--------------------------------------------------------------------------#
##       Estimator Lower Upper Statistic      p.Value
## 2 - 1     0.158 0.100 0.215     6.478 1.348023e-06
## 3 - 1     0.348 0.260 0.430     8.935 1.418199e-12
## 3 - 2     0.190 0.103 0.274     5.164 6.275322e-06
##
##  #----Overall---------------------------------------------------------------------------#
##   Quantile      p.Value
## 1 2.385454 1.418199e-12
##
```

```
##  #----------------------------------------------------------------------------------------#
```

OP_ratio

```r
# the tests...
f2.perc_op_ratio <- mctp(perc_op_ratio ~ group , info=F, data=flash2)
f4.perc_op_ratio <- mctp(perc_op_ratio ~ group , info=F, data=flash4)
f6.perc_op_ratio <- mctp(perc_op_ratio ~ group , info=F, data=flash6)
f8.perc_op_ratio <- mctp(perc_op_ratio ~ group , info=F, data=flash8)
f10.perc_op_ratio <- mctp(perc_op_ratio ~ group ,info=F,  data=flash10)
```

flash -0.119

```r
summary(f2.perc_op_ratio)
```

```
##
##  #----------------Nonparametric Multiple Comparisons for relative effects---------------#
##
##  - Alternative Hypothesis:  True differences of relative effects are not equal to 0
##  - Estimation Method: Global Pseudo ranks
##  - Type of Contrast : Tukey
##  - Confidence Level: 95 %
##  - Method = Fisher with 62 DF
##
##  #----------------------------------------------------------------------------------------#
##
##  #----Data Info---------------------------------------------------------------------------#
##    Sample Size    Effect     Lower     Upper
## 1     ASD  146 0.4636476 0.4272367 0.5004503
## 2 Control  247 0.5015385 0.4679475 0.5351156
## 3    ADHD   45 0.5348139 0.4853801 0.5835731
##
##  #----Contrast----------------------------------------------------------------------------#
##        1  2 3
## 2 - 1 -1  1 0
## 3 - 1 -1  0 1
## 3 - 2  0 -1 1
##
##  #----Analysis----------------------------------------------------------------------------#
##       Estimator  Lower Upper Statistic   p.Value
## 2 - 1     0.038 -0.035 0.110     1.239 0.4274539
## 3 - 1     0.071 -0.045 0.186     1.457 0.3112697
## 3 - 2     0.033 -0.077 0.142     0.720 0.7475260
##
##  #----Overall-----------------------------------------------------------------------------#
##   Quantile   p.Value
## 1 2.383216 0.3112697
##
##  #----------------------------------------------------------------------------------------#
```

flash 0.398

```
summary(f4.perc_op_ratio)
```

```
##
##  #----------------Nonparametric Multiple Comparisons for relative effects---------------#
##
##  - Alternative Hypothesis:  True differences of relative effects are not equal to 0
##  - Estimation Method: Global Pseudo ranks
##  - Type of Contrast : Tukey
##  - Confidence Level: 95 %
##  - Method = Fisher with 79 DF
##
##  #---------------------------------------------------------------------------------------#
##
##  #----Data Info--------------------------------------------------------------------------#
##    Sample Size    Effect     Lower     Upper
## 1     ASD  153 0.4183639 0.3846856 0.4528209
## 2 Control  244 0.5406817 0.5090034 0.5720346
## 3    ADHD   48 0.5409544 0.4990535 0.5822841
##
##  #----Contrast---------------------------------------------------------------------------#
##        1  2 3
## 2 - 1 -1  1 0
## 3 - 1 -1  0 1
## 3 - 2  0 -1 1
##
##  #----Analysis---------------------------------------------------------------------------#
##       Estimator  Lower Upper Statistic      p.Value
## 2 - 1     0.122  0.048 0.195     3.921 0.0005290172
## 3 - 1     0.123  0.021 0.221     2.878 0.0138619039
## 3 - 2     0.000 -0.094 0.095     0.007 0.9999739038
##
##  #----Overall----------------------------------------------------------------------------#
##   Quantile      p.Value
## 1 2.379762 0.0005290172
##
##  #---------------------------------------------------------------------------------------#
```

flash 0.602

```
summary(f6.perc_op_ratio)
```

```
##
##  #----------------Nonparametric Multiple Comparisons for relative effects---------------#
##
##  - Alternative Hypothesis:  True differences of relative effects are not equal to 0
##  - Estimation Method: Global Pseudo ranks
##  - Type of Contrast : Tukey
##  - Confidence Level: 95 %
```

```
##  - Method = Fisher with 78 DF
##
##  #--------------------------------------------------------------------------------------#
##
##  #----Data Info-------------------------------------------------------------------------#
##    Sample Size    Effect     Lower     Upper
## 1     ASD  149 0.4559388 0.4211423 0.4911707
## 2 Control  301 0.5610261 0.5308994 0.5907097
## 3    ADHD   52 0.4830351 0.4418186 0.5244837
##
##  #----Contrast--------------------------------------------------------------------------#
##        1  2 3
## 2 - 1 -1  1 0
## 3 - 1 -1  0 1
## 3 - 2  0 -1 1
##
##  #----Analysis--------------------------------------------------------------------------#
##       Estimator  Lower Upper Statistic     p.Value
## 2 - 1     0.105  0.032 0.177     3.407 0.002858369
## 3 - 1     0.027 -0.075 0.129     0.630 0.800982117
## 3 - 2    -0.078 -0.168 0.014    -2.021 0.111237472
##
##  #----Overall---------------------------------------------------------------------------#
##   Quantile     p.Value
## 1 2.377525 0.002858369
##
##  #--------------------------------------------------------------------------------------#
```

flash 0.949

```
summary(f8.perc_op_ratio)
```

```
##
##  #----------------Nonparametric Multiple Comparisons for relative effects---------------#
##
##  - Alternative Hypothesis:  True differences of relative effects are not equal to 0
##  - Estimation Method: Global Pseudo ranks
##  - Type of Contrast : Tukey
##  - Confidence Level: 95 %
##  - Method = Fisher with 70 DF
##
##  #--------------------------------------------------------------------------------------#
##
##  #----Data Info-------------------------------------------------------------------------#
##    Sample Size    Effect     Lower     Upper
## 1     ASD  149 0.4469361 0.4116550 0.4827598
## 2 Control  252 0.5424890 0.5094284 0.5751795
## 3    ADHD   54 0.5105749 0.4622300 0.5587229
##
##  #----Contrast--------------------------------------------------------------------------#
##        1  2 3
## 2 - 1 -1  1 0
```

```
## 3 - 1 -1  0 1
## 3 - 2  0 -1 1
##
##  #----Analysis--------------------------------------------------------------------------#
##       Estimator  Lower Upper Statistic     p.Value
## 2 - 1     0.096  0.025 0.165     3.214 0.005499333
## 3 - 1     0.064 -0.050 0.176     1.333 0.374282148
## 3 - 2    -0.032 -0.139 0.076    -0.702 0.758014528
##
##  #----Overall---------------------------------------------------------------------------#
##   Quantile     p.Value
## 1 2.377406 0.005499333
##
##  #--------------------------------------------------------------------------------------#
```

flash 1.204

```
summary(f10.perc_op_ratio)
```

```
##
##  #----------------Nonparametric Multiple Comparisons for relative effects---------------#
##
##  - Alternative Hypothesis:  True differences of relative effects are not equal to 0
##  - Estimation Method: Global Pseudo ranks
##  - Type of Contrast : Tukey
##  - Confidence Level: 95 %
##  - Method = Fisher with 69 DF
##
##  #--------------------------------------------------------------------------------------#
##
##  #----Data Info-------------------------------------------------------------------------#
##    Sample Size    Effect     Lower     Upper
## 1     ASD  158 0.4330771 0.4006804 0.4660562
## 2 Control  449 0.6107010 0.5841747 0.6365878
## 3    ADHD   53 0.4562219 0.4142922 0.4987814
##
##  #----Contrast--------------------------------------------------------------------------#
##        1  2 3
## 2 - 1 -1  1 0
## 3 - 1 -1  0 1
## 3 - 2  0 -1 1
##
##  #----Analysis--------------------------------------------------------------------------#
##       Estimator  Lower  Upper Statistic      p.Value
## 2 - 1     0.178  0.117  0.237     6.884 2.401853e-09
## 3 - 1     0.023 -0.079  0.125     0.536 8.486959e-01
## 3 - 2    -0.154 -0.243 -0.064    -4.009 4.025498e-04
##
##  #----Overall---------------------------------------------------------------------------#
##   Quantile      p.Value
## 1 2.369946 2.401853e-09
##
```

```
## #------------------------------------------------------------------------------------#
```

Correlations between groups at different flash strengths and voltages

12 correlation plots for the pairs ADHD-cont, ADHD-ASD, ASD-cont when their microvolots happened at b20-flash=.602, b20-flash=1.20, b40-flash=.602, and b40-flash=1.20.

The idea behind this approach is to identify which is the best option to run with the classification model.

First create some subsets of data…

```
b20_flash_.602 <- subset(data.fixed,
             strength==0.602,
            select=c(b20,group,strength))

b20_flash_1.20 <- subset(data.fixed,
             strength==1.204,
            select=c(b20,group,strength))

b40_flash_.602 <- subset(data.fixed,
             strength==0.602,
            select=c(b40,group,strength))

b40_flash_1.20 <- subset(data.fixed,
             strength==1.204,
            select=c(b40,group,strength))
```

Note that because these aren't paired samples (as needed for traditional correlation), there needs to be some resampling.

The lengths of the vectors of data for ADHD, ASD and control need to be checked first. If one of them is shorter than the other, then samples of the size of the smallest group need to be taken from the largest group. This procedure is then repeated several times and correlation values will be estimated for each resulting bivariate data set (of equal sample size). Below is a simplified example.

```
set.seed(2021) # to get the same result always

# two groups of unequal sample size
group1.large<-rnorm(100)
group2.small<-rnorm(10)

# say i want to see their pearson correlation:
# random samples of size 10 are taken
# from 'group1.large' and are correlated with
# 'group2.small' (this group is thus 'fixed' always)
# this procedure is repeated a few times

number.of.times<-1e3 # usually thousand or more

results<-replicate(number.of.times,
{cor<-cor.test(group2.small,
        sample(group1.large,length(group2.small)))
```

```r
list(cor$estimate, cor$p.value)
})

results <- as.data.frame(t(results))
colnames(results) <-c('correlation', 'p_value')

list(median.p.value=median(as.numeric(results$p_value)),
  median.correlation=median(as.numeric(results$correlation)))
```

```
## $median.p.value
## [1] 0.4998847
##
## $median.correlation
## [1] 0.02425302
```

Correlations are examined via the robust percentage bend correlation method. See Wilcox, R. (2012; *Introduction to Robust Estimation and Hypothesis Testing (3rd ed.)*. Elsevier.) for details.

Groups' sample sizes

First, the lenght of all groups needs to be checked.

```r
# length of the groups
list(group_b20_flash_.602=sort(table(b20_flash_.602$group)),

     group_b20_flash_1.20=sort(table(b20_flash_1.20$group)),

    group_b40_flash_.602=sort(table(b40_flash_.602$group)),

     group_b40_flash_1.20=sort(table(b40_flash_1.20$group))

     )
```

```
## $group_b20_flash_.602
##
##    ADHD     ASD Control
##      52     149     301
##
## $group_b20_flash_1.20
##
##    ADHD     ASD Control
##      53     158     449
##
## $group_b40_flash_.602
##
##    ADHD     ASD Control
##      52     149     301
##
## $group_b40_flash_1.20
##
##    ADHD     ASD Control
##      53     158     449
```

Thus, ADHD will be always the smallest and, therefore, fixed group.

ADHD vs control at flash .602 and b20

```
library(WRS2) # for the robust correlation
```

```
## Warning: package 'WRS2' was built under R version 4.1.1
```

```
set.seed(2021) # to obtain the same results

results1<-replicate(1e3,
             {
adhd<-b20_flash_.602$b20[b20_flash_.602$group=='ADHD']
control<-b20_flash_.602$b20[b20_flash_.602$group=='Control']

cor<-pbcor(adhd, sample(control, length(adhd)),
     nboot = 1e3)

list(cor$p.value, cor$cor)

}
)

results1 <- as.data.frame(t(results1))
colnames(results1) <-c('p_value','correlation')

list(
median.p.value=median(as.numeric(results1$p_value)),
median.pb.correlation=median(as.numeric(results1$correlation))
)
```

```
## $median.p.value
## [1] 0.490927
##
## $median.pb.correlation
## [1] -0.005480626
```

ADHD vs control at flash 1.20 and b20

```
library(WRS2) # for the robust correlation

set.seed(2021) # to obtain the same results

results2<-replicate(1e3,
             {
adhd<-b20_flash_1.20$b20[b20_flash_1.20$group=='ADHD']
control<-b20_flash_1.20$b20[b20_flash_1.20$group=='Control']

cor<-pbcor(adhd, sample(control, length(adhd)),
```

```r
     nboot = 1e3)

list(cor$p.value, cor$cor)

}
)

results2 <- as.data.frame(t(results2))
colnames(results2) <-c('p_value','correlation')

list(
median.p.value=median(as.numeric(results2$p_value)),
median.pb.correlation=median(as.numeric(results2$correlation))
)
```

```
## $median.p.value
## [1] 0.471069
##
## $median.pb.correlation
## [1] -0.0027171
```

ADHD vs control at flash .602 and b40

```r
library(WRS2) # for the robust correlation

set.seed(2021) # to obtain the same results

results3<-replicate(1e3,
             {
adhd<-b40_flash_.602$b40[b40_flash_.602$group=='ADHD']
control<-b40_flash_.602$b40[b40_flash_.602$group=='Control']

cor<-pbcor(adhd, sample(control, length(adhd)),
     nboot = 1e3)

list(cor$p.value, cor$cor)

}
)

results3 <- as.data.frame(t(results3))
colnames(results3) <-c('p_value','correlation')

list(
median.p.value=median(as.numeric(results3$p_value)),
median.pb.correlation=median(as.numeric(results3$correlation))
)
```

```
## $median.p.value
## [1] 0.480317
##
```

```
## $median.pb.correlation
## [1] 0.01306128
```

ADHD vs control at flash 1.20 and b40

```r
library(WRS2) # for the robust correlation

set.seed(2021) # to obtain the same results

results4<-replicate(1e3,
             {
adhd<-b40_flash_1.20$b40[b40_flash_1.20$group=='ADHD']
control<-b40_flash_1.20$b40[b40_flash_1.20$group=='Control']

cor<-pbcor(adhd, sample(control, length(adhd)),
     nboot = 1e3)

list(cor$p.value, cor$cor)

}
)

results4 <- as.data.frame(t(results4))
colnames(results4) <-c('p_value','correlation')

list(
median.p.value=median(as.numeric(results4$p_value)),
median.pb.correlation=median(as.numeric(results4$correlation))
)
```

```
## $median.p.value
## [1] 0.4803136
##
## $median.pb.correlation
## [1] -0.002202119
```

ASD vs control at flash .602 and b20

```r
library(WRS2) # for the robust correlation

set.seed(2021) # to obtain the same results

results5<-replicate(1e3,
             {
asd<-b20_flash_.602$b20[b20_flash_.602$group=='ASD']
control<-b20_flash_.602$b20[b20_flash_.602$group=='Control']

cor<-pbcor(asd, sample(control, length(asd)),
     nboot = 1e3)
```

```
list(cor$p.value, cor$cor)

}
)

results5 <- as.data.frame(t(results5))
colnames(results5) <-c('p_value','correlation')

list(
median.p.value=median(as.numeric(results5$p_value)),
median.pb.correlation=median(as.numeric(results5$correlation))
)
```

```
## $median.p.value
## [1] 0.5264402
##
## $median.pb.correlation
## [1] 0.002676836
```

ASD vs control at flash 1.20 and b20

```
library(WRS2) # for the robust correlation

set.seed(2021) # to obtain the same results

results6<-replicate(1e3,
             {
asd<-b20_flash_1.20$b20[b20_flash_1.20$group=='ASD']
control<-b20_flash_1.20$b20[b20_flash_1.20$group=='Control']

cor<-pbcor(asd, sample(control, length(asd)),
     nboot = 1e3)

list(cor$p.value, cor$cor)

}
)

results6 <- as.data.frame(t(results6))
colnames(results6) <-c('p_value','correlation')

list(
median.p.value=median(as.numeric(results6$p_value)),
median.pb.correlation=median(as.numeric(results6$correlation))
)
```

```
## $median.p.value
## [1] 0.5138768
##
## $median.pb.correlation
## [1] -0.001041595
```

ASD vs control at flash .602 and b40

```
library(WRS2) # for the robust correlation

set.seed(2021) # to obtain the same results

results7<-replicate(1e3,
             {
asd<-b40_flash_.602$b40[b40_flash_.602$group=='ASD']
control<-b40_flash_.602$b40[b40_flash_.602$group=='Control']

cor<-pbcor(asd, sample(control, length(asd)),
     nboot = 1e3)

list(cor$p.value, cor$cor)

}
)

results7 <- as.data.frame(t(results7))
colnames(results7) <-c('p_value','correlation')

list(
median.p.value=median(as.numeric(results7$p_value)),
median.pb.correlation=median(as.numeric(results7$correlation))
)
```

```
## $median.p.value
## [1] 0.5083853
##
## $median.pb.correlation
## [1] 0.002662544
```

ASD vs control at flash 1.20 and b40

```
library(WRS2) # for the robust correlation

set.seed(2021) # to obtain the same results

results8<-replicate(1e3,
             {
asd<-b40_flash_1.20$b40[b40_flash_1.20$group=='ASD']
control<-b40_flash_1.20$b40[b40_flash_1.20$group=='Control']

cor<-pbcor(asd, sample(control, length(asd)),
     nboot = 1e3)

list(cor$p.value, cor$cor)

}
)
```

```r
results8 <- as.data.frame(t(results8))
colnames(results8) <-c('p_value','correlation')

list(
median.p.value=median(as.numeric(results8$p_value)),
median.pb.correlation=median(as.numeric(results8$correlation))
)
```

```
## $median.p.value
## [1] 0.5207586
##
## $median.pb.correlation
## [1] 0.00122891
```

ADHD vs ASD at flash .602 and b20

```r
library(WRS2) # for the robust correlation

set.seed(2021) # to obtain the same results

results9<-replicate(1e3,
             {
adhd<-b20_flash_.602$b20[b20_flash_.602$group=='ADHD']
asd<-b20_flash_.602$b20[b20_flash_.602$group=='ASD']


cor<-pbcor(adhd, sample(asd, length(adhd)),
     nboot = 1e3)

list(cor$p.value, cor$cor)

}
)

results9 <- as.data.frame(t(results9))
colnames(results9) <-c('p_value','correlation')

list(
median.p.value=median(as.numeric(results9$p_value)),
median.pb.correlation=median(as.numeric(results9$correlation))
)
```

```
## $median.p.value
## [1] 0.49324
##
## $median.pb.correlation
## [1] 0.001778793
```

ADHD vs ASD at flash 1.20 and b20

```
library(WRS2) # for the robust correlation

set.seed(2021) # to obtain the same results

results10<-replicate(1e3,
             {
adhd<-b20_flash_1.20$b20[b20_flash_1.20$group=='ADHD']
asd<-b20_flash_1.20$b20[b20_flash_1.20$group=='ASD']


cor<-pbcor(adhd, sample(asd, length(adhd)),
     nboot = 1e3)

list(cor$p.value, cor$cor)

}
)

results10 <- as.data.frame(t(results10))
colnames(results10) <-c('p_value','correlation')

list(
median.p.value=median(as.numeric(results10$p_value)),
median.pb.correlation=median(as.numeric(results10$correlation))
)
```

```
## $median.p.value
## [1] 0.5212486
##
## $median.pb.correlation
## [1] -0.001459142
```

ADHD vs ASD at flash .602 and b40

```
library(WRS2) # for the robust correlation

set.seed(2021) # to obtain the same results

results11<-replicate(1e3,
             {
adhd<-b40_flash_.602$b40[b40_flash_.602$group=='ADHD']
asd<-b40_flash_.602$b40[b40_flash_.602$group=='ASD']


cor<-pbcor(adhd, sample(asd, length(adhd)),
     nboot = 1e3)

list(cor$p.value, cor$cor)
```

```r
}
)

results11 <- as.data.frame(t(results11))
colnames(results11) <-c('p_value','correlation')

list(
median.p.value=median(as.numeric(results11$p_value)),
median.pb.correlation=median(as.numeric(results11$correlation))
)
```

```
## $median.p.value
## [1] 0.5017813
##
## $median.pb.correlation
## [1] 0.00409274
```

ADHD vs ASD at flash 1.20 and b40

```r
library(WRS2) # for the robust correlation

set.seed(2021) # to obtain the same results

results12<-replicate(1e3,
             {
adhd<-b40_flash_1.20$b40[b40_flash_1.20$group=='ADHD']
asd<-b40_flash_1.20$b40[b40_flash_1.20$group=='ASD']


cor<-pbcor(adhd, sample(asd, length(adhd)),
     nboot = 1e3)

list(cor$p.value, cor$cor)

}
)

results12 <- as.data.frame(t(results12))
colnames(results12) <-c('p_value','correlation')

list(
median.p.value=median(as.numeric(results12$p_value)),
median.pb.correlation=median(as.numeric(results12$correlation))
)
```

```
## $median.p.value
## [1] 0.4891338
##
## $median.pb.correlation
## [1] -0.01085292
```
